# Bottom-Up Synthesis of Multiply Fused Pd^II^ Anthriporphyrinoids

**DOI:** 10.1021/acscentsci.2c01218

**Published:** 2022-12-08

**Authors:** Xinrun Ge, Yutao Rao, Ling Xu, Mingbo Zhou, Ryo Kurosaki, Naoki Aratani, Atsuhiro Osuka, Jianxin Song

**Affiliations:** † Key Laboratory of Chemical Biology and Traditional Chinese Medicine, Ministry of Educational of China, Key Laboratory of the Assembly and Application of Organic Functional Molecules of Hunan Province, Hunan Normal University, Changsha 410081, China; ‡ Division of Materials Science, Nara Institute of Science and Technology (NAIST) 8916-5 Takayama-cho, Ikoma 630-0192, Japan

## Abstract

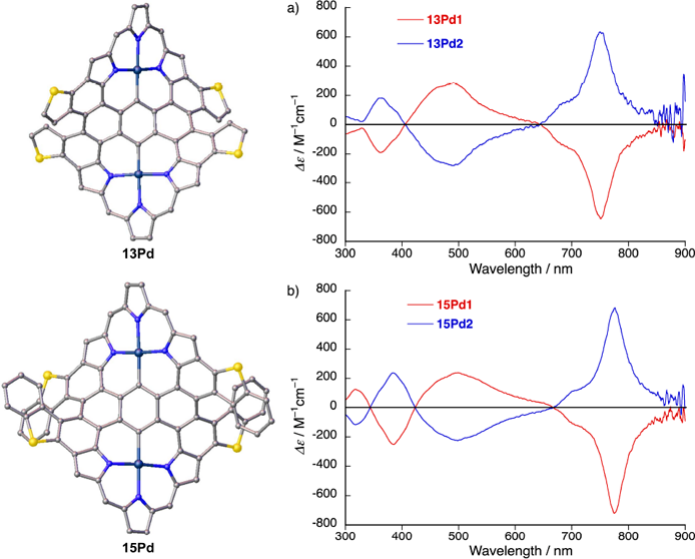

Anthriporphyrinoid and its dimeric homologues were synthesized by Suzuki–Miyaura coupling and subsequent oxidation. Both porphyrinoids were smoothly converted to their Pd^II^ complexes and were further decorated by Suzuki–Miyaura coupling with thiophene derivatives and subsequent oxidative fusion reaction to provide multiply fused compounds. Most Pd^II^ anthriporphyrinoids have been structurally well characterized to be planar for monomeric and helically twisted for dimeric species. The dimeric anthriporphyrinoids show paratropic ring currents due to their global antiaromatic networks, the extent of which increases with an increase of conjugated network. Multiply fused dimeric anthriporphyrinoids show helical structures, fully reversible six redox potentials, small HOMO–LUMO gaps, and absorption tails reaching in the near-infrared region, suggesting the high potential of this approach to explore molecular graphene. Optical separations of the dimeric helical species were accomplished, and racemization barrier heights were determined.

## Abstract 2

Multiply thiophene- and benzothiophene-fused Pd^II^ anthriporphyrinoids were synthesized. While the monomeric complexes are nonaromatic, the dimeric complexes show paratropic ring currents owing to the global 32π- and 36π-electronic circuits. Multiple fusions of the dimeric complexes allowed six fully reversible redox potentials, small HOMO−LUMO gaps, and broad absorption tails reaching at NIR, and further, caused helical conformations. The optical separations were accomplished and their racemization barrier heights were determined.

## 1 Introduction

**Scheme 1:**
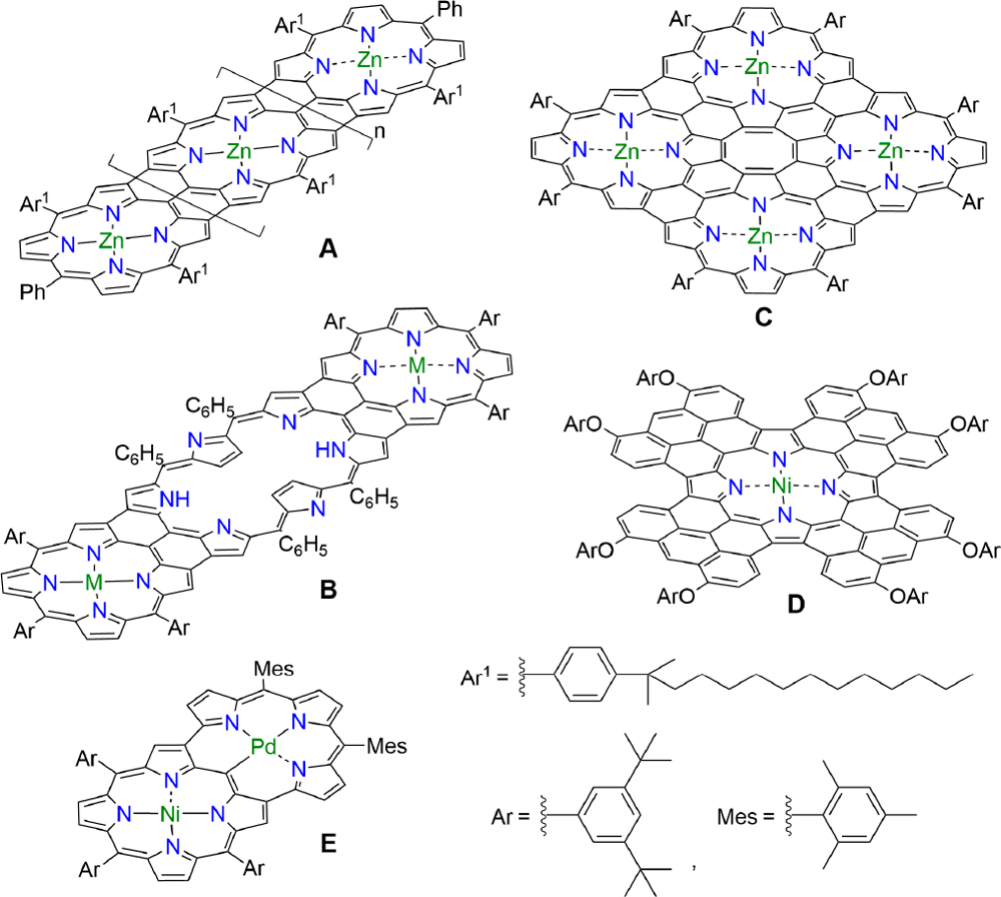
Multiply Fused Porphyrinoids (A, B, C, and D) and Earring Porphyrin E

“Molecular-graphenes” represent a class of discrete 2-dimensional and highly conjugated molecular sheets, which is widely regarded as the molecular model of graphene.^1,2^ These species are useful in understanding the structure–property relationships of graphenes and stimulating the organic synthesis of graphene materials with a discrete structure. Porphyrinoids can be an important structural motif of molecular graphenes. Representative examples include porphyrin tapes **A** that exhibit the remarkably strong absorption reaching in the infrared region,^3^ [26]hexaphyrin doubly fused with porphyrins **B**,^4^ a porphyrin sheet, **C**, that has a strong paratropic ring current just at the central planar cyclooctatetraene,^5^ and a porphyrin fused with four anthracene units, **D**.^6−9^ These compounds constitute a subgroup of “porphyrinoid molecular graphenes”, which are promising in light of the remarkable optical and electronic properties of porphyrins (Scheme 1).

**Scheme 2:**
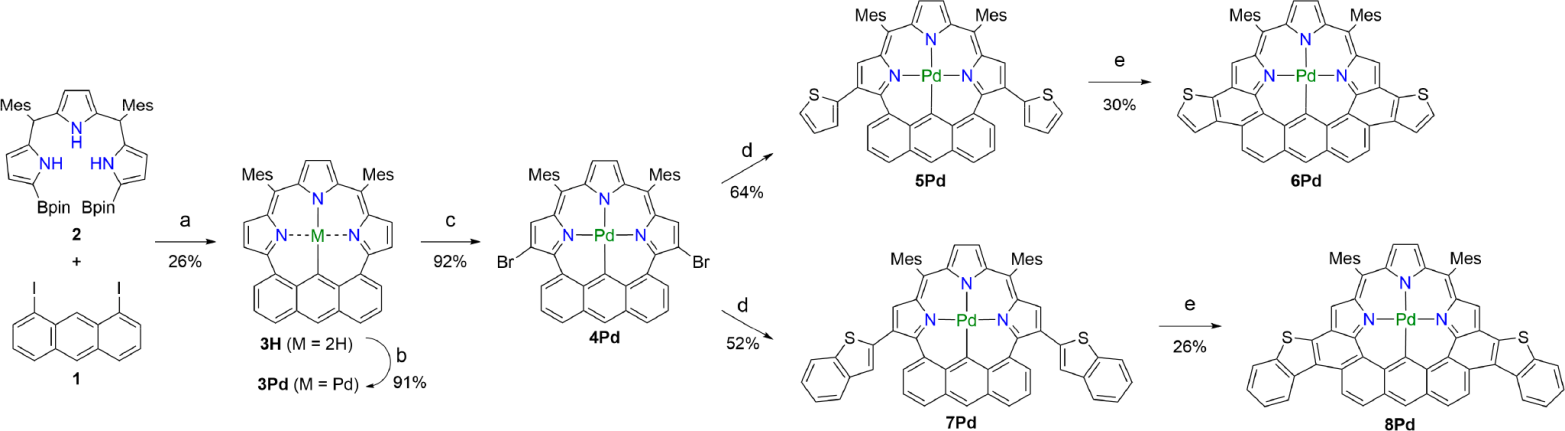
Synthesis of Anthriporphyrinoids 6Pd and 18Pd Conditions and reagents: (a) Pd_2_(dba)_3_, Sphos, Cs_2_CO_3_, CsF, Toluene, DMF, 115 °C; (b) Pd(OAc)_2_, NaOAc, CH_2_Cl_2_, MeOH; (c) NBS, CH_2_Cl_2_, 25 °C; (d) 2-thienylboronic acid, Pd_2_(dba)_3_, PPh_3_, Cs_2_CO_3_, CsF, toluene, DMF, 115 °C (for 5**Pd**); 2-benzothienylboronic acid, Pd_2_(dba)_3_, PPh_3_, Cs_2_CO_3_, CsF, toluene, DMF, 115 °C (for **7Pd**); (e) DDQ, TfOH, CH_2_Cl_2_, 0 °C. Mes = 2,4,6-trimethylphenyl, Bpin = pinacolatoboryl.

**Scheme 3:**
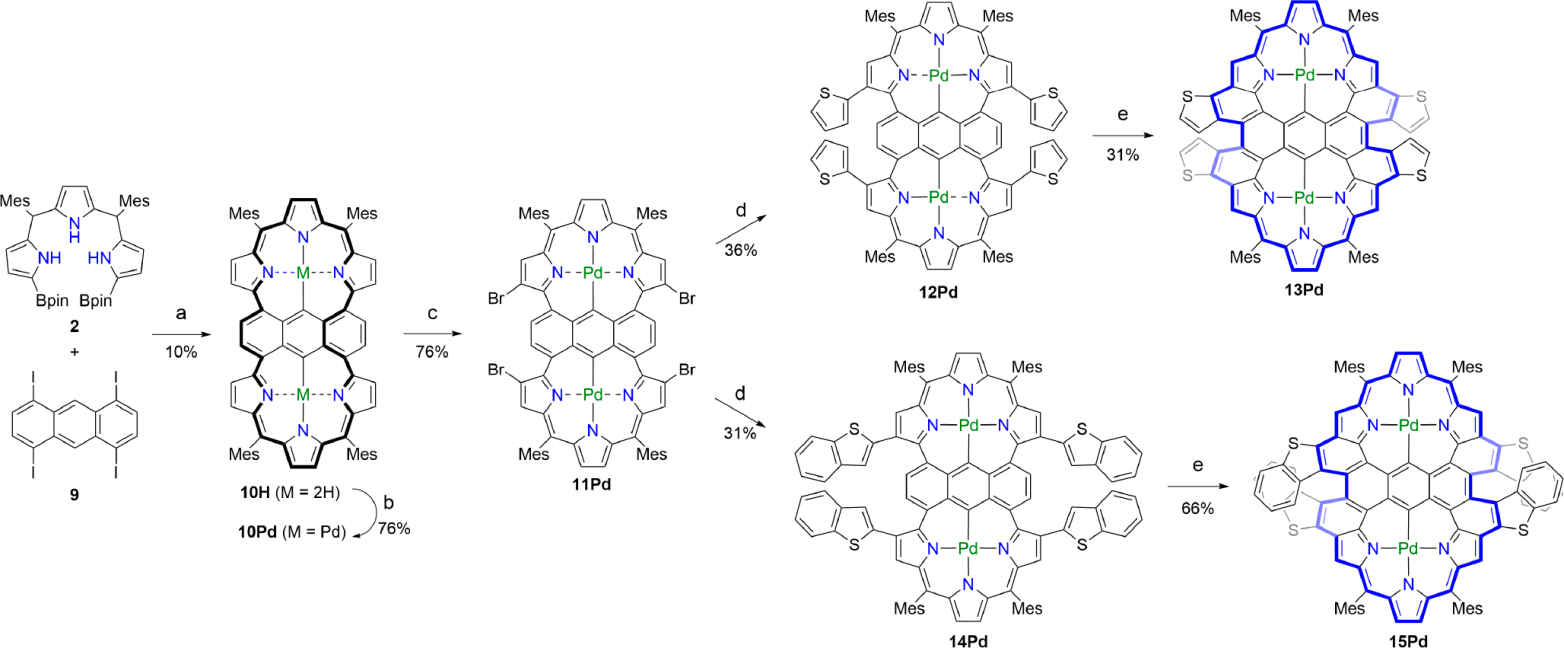
Synthesis of Anthracene-Sharing Dimeric Porphyrinoids 13Pd and 15Pd Conditions and reagents: (a) Pd_2_(dba)_3_, Sphos, Cs_2_CO_3_, CsF, toluene, DMF, 115 °C; (b) Pd(OAc)_2_, NaOAc, CH_2_Cl_2_, MeOH; (c) NBS, CH_2_Cl_2_, 25 °C; (d) 2-thienylboronic acid, Pd_2_(dba)_3_, PPh_3_, Cs_2_CO_3_, CsF, toluene, DMF, 115 °C (for **12Pd**); 2-benzothienylboronic acid, Pd_2_(dba)_3_, PPh_3_, Cs_2_CO_3_, CsF, toluene, DMF, 115 °C (for **14Pd**); (e) DDQ, TfOH, CH_2_Cl_2_, 0 °C. Mes = 2,4,6-trimethylphenyl, Bpin = pinacolatoboryl.

In 2016, we reported “earring porphyrin” **E**, in which the tripyrrin moiety is directly linked at the 3,7-positions of a Ni^II^ porphyrin, forming a cavity that can serve as a coordination ligand toward transition metal.^10^ Further fusion of heterocycles on the periphery of earring porphyrins led to multiply fused porphyrinoids with absorption bands reaching at ca. 2200 nm.^11^ These compounds can be regarded as “porphyrinoid molecular graphenes”. As an extension of these studies, we designed and synthesized anthracene-based earring porphyrinoids, anthriporphyrinids such as **3H** and **10H**, as new porphyrinic molecular graphenes, where a tripyrrin segment is directly linked at 1,8-positions or 1,4,5,8-positions of anthracene to form a cavity serving as an effective ligand toward Pd^II^ ion (Schemes 2 and 3).

## 2 Results and Discussion

### 2.1 Synthesis

**Figure 1:**
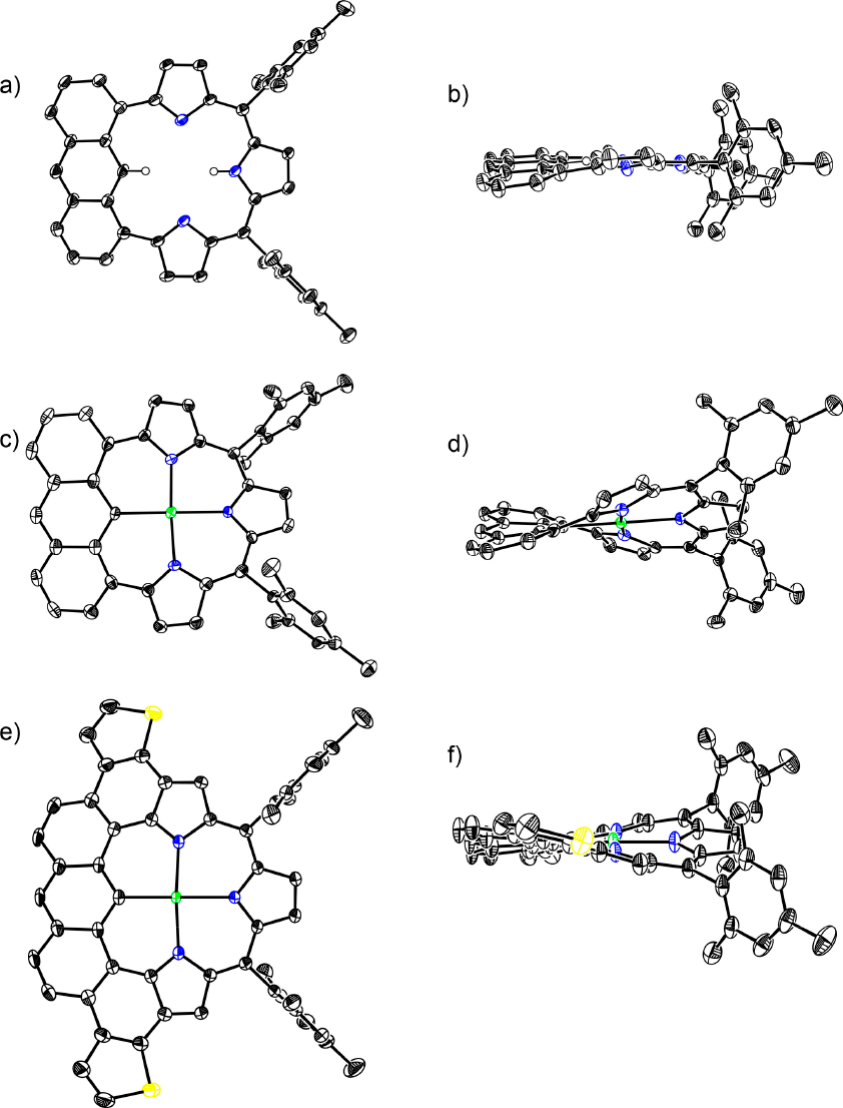
X-ray structures: (a) top view and (b) side view of **3H** (structure of one conformers); (c) top view and (d) side view of **3Pd**; (e) top view and (f) side view of **6Pd**. The thermal ellipsoids were scaled to 50% probability for **3H**, **3Pd**, and **6Pd** (solvent molecules and hydrogen atoms on carbon were omitted for clarity).

Synthesis of anthriporphyrinoids **3H** is shown in Scheme 2. Suzuki–Miyaura cross-coupling reaction of 1,8-diiodoanthracene^12^**1** with α,α′-diboryltripyrrane **2**(10) and subsequent oxidation with DDQ gave 1,8-anthriporphyrin **3H** in 26% yield. In line with the structure, high-resolution matrix-assisted laser desorption ionization time-of-flight (HR-MALDI-TOF) mass measurements showed the parent ion peak of **3H** at *m*/*z* = 631.2870, calcd for (C_46_H_37_N_3_)^+^ = 631.2982 ([M]^+^). The structure of **3H** was confirmed by X-ray diffraction analysis, which showed that there were two different conformations (Figure 1a,b and Supporting Information).

Treatment of **3H** with Pd(OAc)_2_ in the presence of NaOAc in CH_2_Cl_2_/MeOH afforded **3Pd** in 91%. The structure of **3Pd** has been unambiguously determined by X-ray diffraction analysis. **3Pd** exhibits a roughly planar structure with a larger mean-plane-deviation (MPD) of 0.605(4) Å. Pd^II^ ion resides at the center of the macrocycle with C–Pd bond of 2.047(3) Å and three N–Pd bonds of 2.013(3), 2.089(3), and 2.015(3) Å. The diagonal C–N and N–N distances are 4.136(4) Å and 4.025(4) Å, respectively. The ^1^H NMR spectrum of **3Pd** displays four signals at 9.10, 9.01, 8.64, and 7.89 ppm due to the anthracene protons and three signals at 7.79, 7.20, and 6.78 ppm due to the pyrrolic β-protons, suggesting nonaromatic character for the anthriporphyrin part. The nonaromatic nature was also indicated by small negative values (−0.97 to −0.58 ppm) of nucleus-independent chemical shift NICS(0) values (Figure S73).

Gratifyingly, dibrominated anthriporphyrinoid **4Pd** was obtained in a high yield of 92% by bromination with NBS in CH_2_Cl_2_, similarly to the earring porphyrin **E**.^11^ Cross-coupling of **4Pd** with 2-thiopheneboronic acid afforded doubly thiophene-appended **5Pd** and subsequent Scholl reaction with DDQ and TfOH in CH_2_Cl_2_ (1:100) at 0 °C for 5 min furnished doubly thiophene-fused complex **6Pd** in a two-step yield of 19% yield (Scheme 2). X-ray diffraction analysis revealed a *C*_*2v*_-symmetric structure with a MPD value of 0.498(4) Å for **6Pd** (Figure 3). The ^1^H NMR spectrum exhibits a singlet at 7.82 ppm and two doublets at 7.67 and 7.53 ppm due to the anthracene protons, two doublets at 7.24 and 7.03 ppm due to the thiophene protons, and two singlets at 6.18 and 5.93 ppm for the pyrrolic β-protons. The NICS(0) values of **6Pd** at the center of the macrocycle were calculated be to +3.78 to +7.56 ppm (Figure S74).

Similarly, **4Pd** was coupled with 2-benzothienylboronic acid to give **7Pd** in 52%, which was oxidized with DDQ/TfOH in CH_2_Cl_2_ to afford doubly benzothiophene-fused complexes **8Pd** in 26%. The ^1^H NMR spectrum of **8Pd** displays a singlet at 7.77 ppm and two doublets at 8.26 and 8.10 ppm due to the anthracene protons, three signals at 7.68, 7.38, and 7.28 ppm due to the benzothiophene protons and two singlets at 6.21 and 5.93 ppm for the pyrrolic β-protons. The NICS(0) values of **8Pd** at the center of the macrocycle were calculated be to +3.44 to +7.56 ppm (Figure S74).

**Figure 2:**
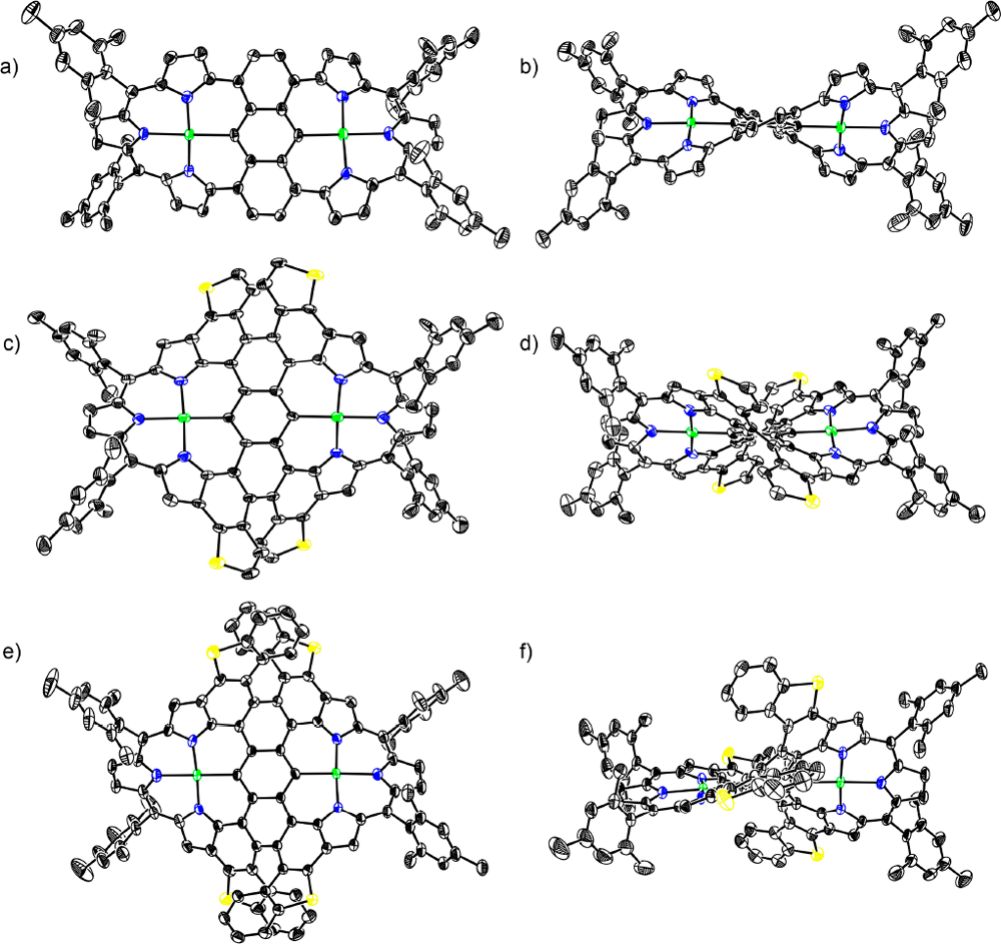
X-ray structures: (a) top view and (b) side view of **10Pd** (structure of one conformer); (c) top view and (d) side view of **13Pd**; (e) top view and (f) side view of **15Pd**. The thermal ellipsoids were scaled to 50% probability for **10Pd**, **13Pd**, and **15Pd** (solvent molecules and hydrogen atoms on carbon were omitted for clarity).

Synthesis of anthracene-sharing dimeric porphyrinoid **10H** is shown in Scheme 3. Reduction of 1,4,5,8-tetrachloroanthraquinone with NaBH_4_ in isopropanol gave 1,4,5,8-tetrachloroanthracene,^13^ which was converted to 1,4,5,8-tetraiodoanthracene (**9**) via borylation^14,15^ and subsequent iodination.^10,16−22^ Under similar conditions, reaction of **9** with **2** gave dimer **10H** in 10% yield. **10Pd** was readily obtained in 76% yield upon treatment of **10H** with Pd(OAc)_2_ under similar conditions. The structure of **10Pd** was unambiguously determined by X-ray diffraction analysis. Two different conformations were found in the crystal of **10Pd**. Both conformations are helically twisted with large twist angles of 67.54(6)° and 67.54(5)° for the two tripyrrin units. The anthracene unit of **10Pd** is also highly distorted with twist angles of 47.8(2)° and 46.9(3)° (Figure 2).

The ^1^H NMR spectrum of **10Pd** is symmetric, showing a singlet at 6.95 ppm due to the anthracene protons and three signals at 6.21, 6.07, and 5.58 ppm due to the pyrrolic β-protons, which are distinctly high-field shifted as compared with those of **3Pd**, suggesting a weak paratropic ring current for **10Pd**. Actually, the NICS(0) values of **10Pd** were calculated to be +8.51 to +8.81 ppm (Figure S73), and anisotropy of induced current density (AICD) plots of **10Pd** showed a counterclockwise circuit. This may be ascribed to the 32π-electronic global network as indicated by the bold black line in Scheme 3.

Bromination of **10Pd** with NBS in CH_2_Cl_2_ gave tetrabrominated Pd^II^ complexes **11Pd** in 42%. Under the same reaction conditions, **11Pd** was converted to quadruply thiophene-appended dimer **12Pd** in 36% yield, which was oxidized with DDQ and TfOH to give **13Pd** in 31% yield. The structure of **13Pd** was also confirmed by X-ray analysis. Two similar but different conformations were found in the crystal of **13Pd**. Both structures display a twisted conformation consisting of two Pd^II^ coordinated porphyrinoids with MPDs of 0.643(13) and 0.610(14) Å and of 0.632(13) and 0.624(13) Å. The dihedral angles of the two porphyrinoids were 43.69(6)° and 43.21(6)°, and the anthracene unit of **13Pd** is also highly distorted with twist angles of 53.4(5)° and 51.6(4)° (Figure 2). Intriguingly, **13Pd** contains two dithia[5]helicene segments. The ^1^H NMR spectrum of **13Pd** displays two doublets at 6.54 and 6.18 ppm due to the thiophene protons and two singlets at 4.47 and 4.45 ppm due to the pyrrolic β-protons. It is apparent that the chemical shifts of the pyrrolic β-protons of **13Pd** are high-field shifted as compared with **10Pd**, indicating a larger paratropic ring current. In line with this, the NICS(0) values in the center of macrocycle were calculated to be +13.75 to +16.58 ppm (Figure S74). Further, the AICD plot of **13Pd** displayed a moderate global paratropic ring current. This may be ascribed to the presence of an additional global 36π-electronic network in **13Pd**, as indicated in blue in Scheme 3, besides the 32π-electronic network.

Finally, **11Pd** were coupled with 2-benzothienylboronic acid to give benzothiophene-appended Pd^II^ complexes **14Pd** in 31% yield, which was oxidized with DDQ and TfOH in CH_2_Cl_2_ to afford quadruply benzothiophene-fused complexes **15Pd** in 66% yield.

The structure of **15Pd** has been revealed by X-ray diffraction analysis to be considerably twisted owing to the multiply fused structures. Actually, **15Pd** possesses two dithia[7]helicene segments at the periphery. The two roughly planar Pd^II^ porphyrinoids segments show MPD of 0.578(4) and 0.545(4) Å, and the dihedral angle of these units is 40.84(2)°, and the anthracene unit of **15Pd** is also highly distorted with twist angles of 52.93(14)° (Figure 2). The ^1^H NMR spectrum of **15Pd** shows signals at 6.78–6.74 and 6.51–6.47 ppm due to the benzothiophene protons and two singlets at 4.43 and 4.42 ppm for the pyrrolic β-protons. These data suggest an enhanced paratropic ring current of **15Pd** as compared with **13Pd**. The NICS(0) values were calculated to be +14.11 to +17.09 ppm (Figure S74), and the AICD plot showed a clear global paratropic ring current.

### 2.2 UV/vis/NIR Absorption Spectra

**Figure 3:**
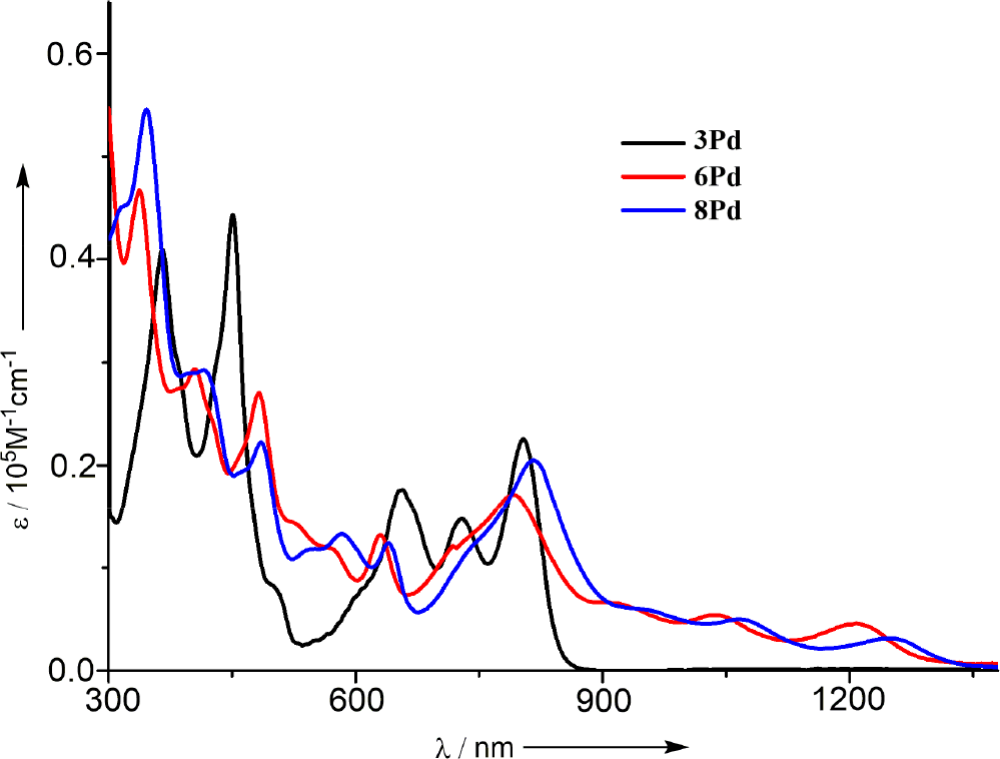
UV/vis/NIR absorption spectra of **3Pd**, **6Pd**, and **8Pd** in CH_2_Cl_2_.

The UV/vis/NIR absorption spectra of **3Pd**, **6Pd**, and **8Pd** are shown in Figure 3. The absorption spectrum of **3Pd** shows structured bands in the range of 600–880 nm, which are distinctly red-shifted as compared with that of **3H** (SI). Intriguingly, the absorption spectra of fused anthriporphyrinoids **6Pd** and **8Pd** display broad and red-shifted absorption bands up to 1350 nm as a rare case of monomeric porphyrinoid, indicating that the peripheral fusion is really effective to reduce the HOMO–LUMO gaps.

**Figure 4:**
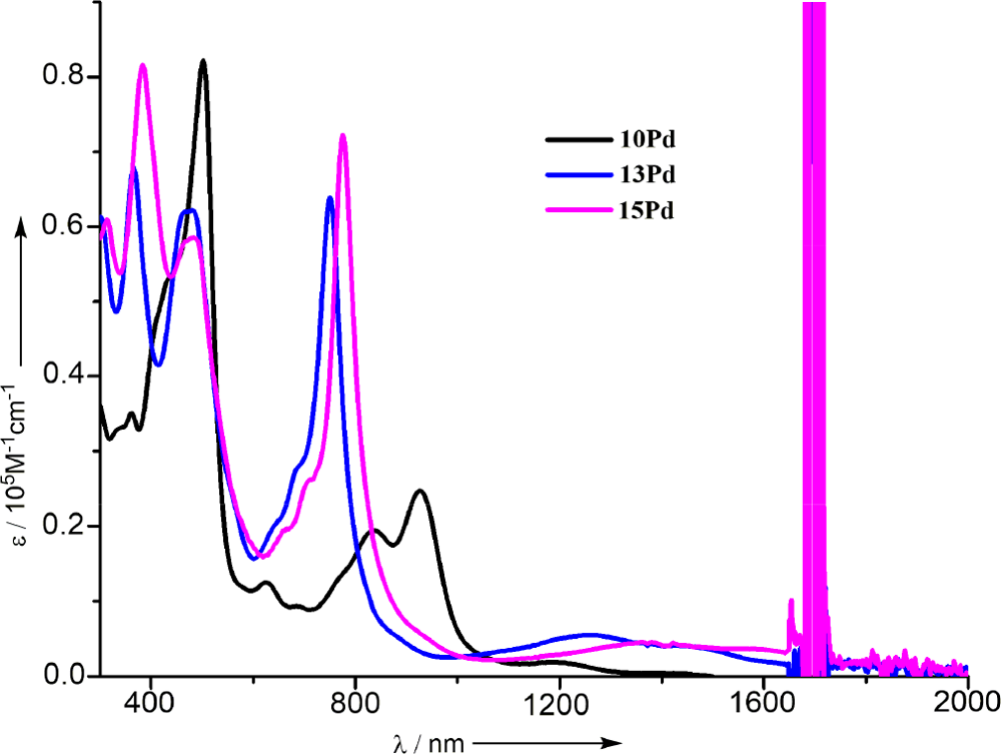
UV/vis/NIR absorption spectra of **10Pd**, **13Pd**, and **15Pd** in CH_2_Cl_2_.

The absorption spectra of the dimeric Pd^II^ complexes **10Pd**, **13Pd**, and **15Pd** are shown in Figure 4. It is notable that these complexes show strong absorption bands at 400, 450, and 800 nm, along with a weak absorption tail reaching at 1300, 1700, and 1900 nm for **10Pd**, **13Pd**, and **15Pd**, respectively. These spectral features are consistent with their antiaromatic character. On the basis of these results, their optical HOMO–LUMO gaps have been estimated to be 0.95, 0.73, and 0.65 eV, respectively, indicating that the peripheral fusion is effective to lower the HOMO–LUMO gap.

### 2.3 Electrochemical Properties

The electrochemical properties were investigated by cyclic voltammetry and differential pulse voltammetry. On the basis of the data in Table 1, the HOMO–LUMO gaps of **3Pd** and **10Pd** were estimated to be respectively smaller than those of **3H** and **10H**, and the HOMO–LUMO gaps of the dimeric complexes (**10Pd**, **13Pd**, and **15Pd**) are smaller than those of the monomeric complexes (**3Pd**, **6Pd**, and **8Pd**). The HOMO–LUMO gaps decreased in the order of **3Pd** (1.60 eV) > **6Pd** (1.23 eV) > **8Pd** (1.20 eV) > **10Pd** (0.97 eV) > **13Pd** (0.81 eV) > **15Pd** (0.78 eV). Remarkably, dimeric complex **10Pd** showed two oxidation waves and four reduction waves. While the fourth reduction wave was quasi reversible, the other waves were reversible. Fused complexes **13Pd** and **15Pd** showed two reversible oxidation waves and four reversible reduction waves, all being at considerably higher potentials as compared with **10Pd**. Naturally, the HOMO–LUMO gaps of **13Pd** and **15Pd** are quite small, being 0.81 and 0.78 eV, respectively. These properties encourage the potential use of these fused anthriporphyrinoids as an electron reservoir. These electrochemical data are roughly in accord with the absorption spectra and TD-DFT calculation (Figures S75–82).

**Table 1:** Electrochemical Potentials in CH_2_Cl_2_^a^

	*E*_red.4_	*E*_red.3_	*E*_red.2_	*E*_red.1_	*E*_ox.1_	*E*_ox.2_	Δ*E*_HL_^b^
**3H**	—	—	–1.68	–1.33	0.56^c^	—	1.89
**3Pd**	—	—	–1.76	–1.15	0.45	—	1.60
**6Pd**^d^	—	—	–1.34	–0.72	0.51^c^	—	1.23
**8Pd**^d^	—	—	–1.22	–0.65	0.55^c^	—	1.20
**10H**	—	—	–1.24	–1.11	0.42	0.57	1.53
**10Pd**	–2.48^e^	–2.19	–1.03	–0.75	0.22	0.52	0.97
**13Pd**	–1.77	–1.55	–0.79	–0.44	0.37	0.70	0.81
**15Pd**	–1.81	–1.56	–0.74	–0.36	0.42	0.74	0.78

a Potentials [V] vs ferrocene/ferrocenium ion. Scan rate 0.05 V s^–1^; glassy carbon working electrode, Pt wire counter electrode, Ag/AgNO_3_ reference electrode, supporting electrolyte 0.1 M nBu_4_NPF_6_ in CH_2_Cl_2_.

b Electrochemical HOMO–LUMO gaps (Δ*E*_HL_ = *E*_ox.1_ – *E*_red.1_ [eV]).

c Irreversible peaks.

d Solubilities are very poor.

e Quasi reversible.

### 2.4 Circular Dichroism and Racemization Barrier

**Figure 5:**
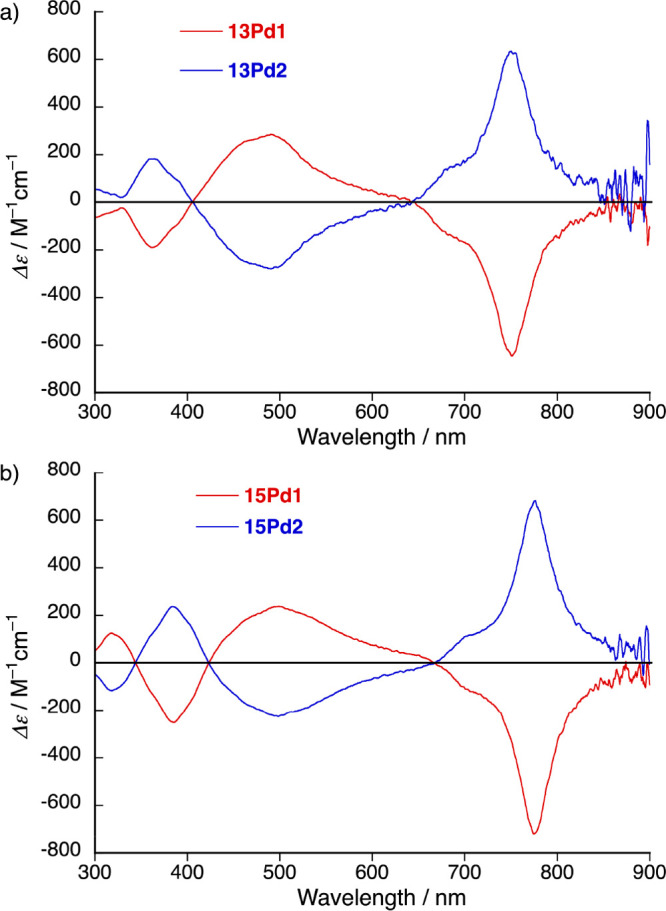
CD spectra of (a) **13Pd** and (b) **15Pd** in the range from 300 to 900 nm in hexane/CH_2_Cl_2_ (1:1).

Subsequently, the largely twisted **13Pd** and **15Pd** were subjected to enantiomeric resolution using chiral column chromatography (Figure S91). The chromatograms of **13Pd** and **15Pd** racemates in hexane/CH_2_Cl_2_ (1:1) did show two well-separated peaks. The isolated first and second peaks clearly provided mirror-signed circular dichroism (CD) spectral profiles, indicating that enantiomeric resolution was achieved (Figure 5). Both CD spectra (i.e., for **13Pd** and **15Pd**) were simulated using the SpecDis software package^23−25^ with high similarity factors (0.91 for **13Pd** and 0.86 for **15Pd**, respectively) (Figures S92 and S93), and thus, the absolute structure of the second eluent **13Pd2** was determined with high accuracy as (*M*,*M*), and the first eluent **13Pd1** was determined as (*P*,*P*). The absolute structure of the second eluent **15Pd2** was determined as (*M*,*M*) as well, while the first eluent **15Pd1** was determined to be (*P*,*P*).

The activation barrier of the twist inversion processes (Δ*G*^‡^_298_) of **13Pd** in toluene was experimentally determined as 113 kJ mol^–1^ at 298 K (Figure S94). The Eyring plot provides the enthalpy (Δ*H*^‡^ = 53.1 kJ mol^–1^) and the entropy (Δ*S*^‡^ = −0.20 kJ mol^–1^ K^–1^) for the racemization process. No racemization of **15Pd** between the (*P,P*)- and (*M,M*)-isomers was detected in toluene at 100 °C for 15 h, suggesting the high configurational stability of **15Pd**.

## 3 Conclusions

In summary, Pd^II^ complexes of anthriporphyrinoid and its dimeric homologues were synthesized and further decorated to multiply thiophene- and benzothiophene-fused compounds. While the monomeric Pd^II^ complexes are nonaromatic, the dimeric complexes show paratropic ring currents owing to the global 32π- and 36π-electronic circuits. Multiple fusions of the dimeric complexes allowed fully reversible six redox potentials, small HOMO–LUMO gaps, and broad absorption tails reaching at NIR, and further, caused helical conformations. Finally, their optical separations were accomplished and their racemization barrier heights were determined.

## Supporting Information Available

This material is available free of charge via the Internet at http://pubs.acs. The Supporting Information is available free of charge at https://pubs.acs.org/doi/10.1021/acscentsci.2c01218.

Experimental procedures and characterization data for all products, including ^1^H NMR, UV–vis, X-ray single crystal data and computational work (PDF)
