# Supplementary material for: Bottom-Up Synthesis of Multiply Fused PdII Anthriporphyrinoids
Source: ACS Cent Sci. 2022 Dec 8;8(12):1627–32. doi: 10.1021/acscentsci.2c01218 (PMC9801503; doi:10.1021/acscentsci.2c01218)
Supplement: Supplementary file 1 — oc2c01218_si_001.pdf [file oc2c01218_si_001.pdf]

Supporting Information

Bottom-Up Synthesis of Multiply Fused Pd^{II} Anthriporphyrinoids

Xinrun Ge,[1] Yutao Rao,[*,1] Ling Xu,[1] Mingbo Zhou,[1] Ryo Kurosaki,[2] Naoki Aratani,[2] Atsuhiro Osuka,[*,1] and Jianxin Song[*,1]

[1]Key Laboratory of Chemical Biology and Traditional Chinese Medicine, Ministry of Educational of China, Key Laboratory of the Assembly and Application of Organic Functional Molecules of Hunan Province, Hunan Normal University, Changsha 410081, China

[2]Division of Materials Science, Nara Institute of Science and Technology (NAIST) 8916-5 Takayama-cho, Ikoma 630-0192, Japan

[*]E-mail: yutaorao2020@hunnu.edu.cn, atsuhiroosuka@hunnu.edu.cn, jxsong@hunnu.edu.cn

Table of Contents

Instruments and Materials ………………………………………………………………. S3

Experimental Procedures ………………………………………………………………. S4

Spectra of Compounds ……………………………………………….…………… S16

Electrochemical Data………………………………………………………………… S46

X-Ray Crystal Data…………………………………………………………………...S51

DFT/Calculations ……………………………………………………………………...S65

Chiral Resolution and Racemization Experiments …………………………………...S79

Instruments and Materials

^{1}H NMR (500 MHz) spectra were taken on a Bruker AVANCE-500 spectrometer, and chemical shifts were reported as the delta scale in ppm relative to $CHCl_3$ as the internal reference for ^{1}H NMR (δ = 7.260 ppm). UV/Vis absorption spectra were recorded on a Shimadzu UV-3600 spectrometer. Fluorescence spectra were recorded on a Hitachi F-7100 spectrometer. IR spectra were recorded on a Nicolet 670 FTIR spectrometer. Mass spectra were obtained with a Bruker ultrafleXtreme MALDI-TOF/TOF spectrometer and Thermo Scientific Q Exactive APCI spectrometer. X-ray data were taken on an Agilent Supernova X-Ray diffractometer equipped with a large area CCD detector. Redox potentials were measured by cyclic voltammetry on a CHI900 scanning electrochemical microscope. Chiral resolutions were performed using ϕ10 × 250 mm SUMICHIRAL OA-3100 (Sumika Chemical Analysis Ltd.) fitted to a preparative HPLC system, which was constructed using a JASCO UV-2075 Plus detector and a JASCO PU-2086 Plus pump. Eluent: hexane/CH_2Cl_2 = 1/1 (*v*/*v*), flow rate: 1.0 mL/min, and detection: UV absorption at 420 nm. CD spectra were recorded using a JASCO J-820 spectropolarimeter. Unless otherwise noted, materials obtained from commercial suppliers were used without further purification.

Experimental Procedures

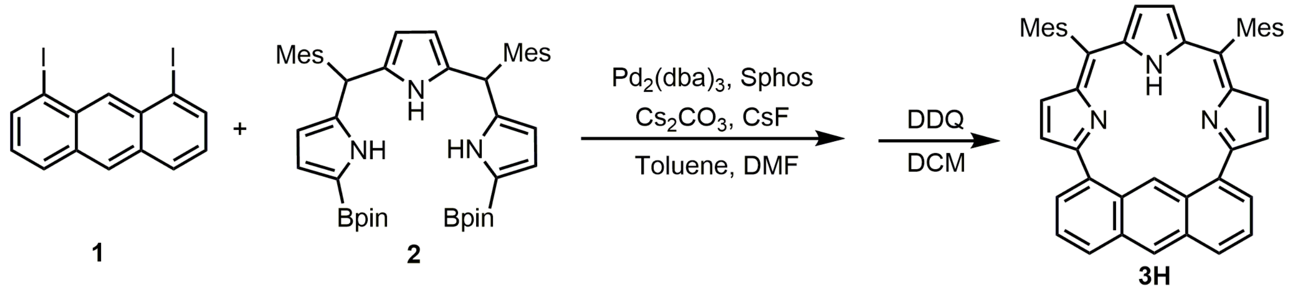

Synthesis of 3H: A solution of 1,8-diiodoanthracene **1** (129 mg, 0.3 mmol), diboryltripyrrane **2** (285 mg, 0.4 mmol), $Pd_2(dba)_3$ (27.5 mg, 0.03 mmol), Sphos (49.3 mg, 0.12 mmol), Cs_2CO_3 (195.5 mg, 0.6 mmol), and CsF (91.1 mg, 0.6 mmol) in a mixture of toluene–DMF (6 mL/3 mL) was degassed through three freeze-pump-thaw cycles, and the reaction flask was purged with argon. The resulting mixture was heated at 115 °C for 48 h. The reaction mixture was diluted with $CHCl_3$, washed with water, and dried over anhydrous sodium sulfate. After the solvent was evaporated in vacuo, the residue was dissolved in CH_2Cl_2 (20 mL), and oxidized with DDQ (136 mg, 0.6 mmol). After being stirred for 10 min, the mixture was passed through a short silica-gel and Al_2O_3 column (CH_2Cl_2 as an eluent). The product was separated by column chromatography on silica-gel with CH_2Cl_2/*n*-hexane as an eluent. Recrystallization from CH_2Cl_2/MeOH gave **3H** (50 mg, 0.079 mmol, 26% yield) as purple solids.

3H: ^{1}H NMR (500 MHz, $CDCl_3$) δ = 11.59 (s, 1H, N-H), 10.01 (s, 1H, An-H), 8.94 (s, 1H, An-H), 8.79 (d, J = 7.0 Hz, 2H, An-H), 8.42 (d, J = 8.0 Hz, 2H, An-H), 7.78 (m, 2H, An-H), 7.59 (d, J = 4.5 Hz, 2H, Py-H), 7.00-6.98 (m, 6H, Ar-H and Py-H), 6.37 (d, J = 2.0 Hz, 2H, Py-H), 2.41 (s, 6H, Me-H), and 2.14 (s, 12H, Me-H) ppm. ^{13}C NMR (126 MHz, CDCl3) δ = 170.3, 153.5, 138.3, 137.6, 137.4, 135.2, 134.6, 134.0, 132.7, 132.6, 131.2, 130.5, 130.1, 129.0, 127.9, 127.1, 124.6, 124.5, 121.3, 21.2, and 20.3 ppm. λ_{max} (ε [$M^{-1}cm^{-1}$]) = 339 (40000), 392 (31000), 413 (31000), 570 (13000), 607 (15000), and 654 (13000) nm. IR (KBr disk): ν = 3435, 2924, 2854, 1583, 1369, 1306, 1257, 1184, 1014, 937, 827, 800, 739 cm^{-1}. Fluorescence (CH_2Cl_2, λ_{ex} = 418 nm): λ_{max} = 684 nm, ϕ_F = 0.3%. HR-MS (MALDI-TOF-MS): m/z = 631.2870, calcd for $(C_{46}H_{37}N_3)^+$ = 631.2982 ($[M]^+$).

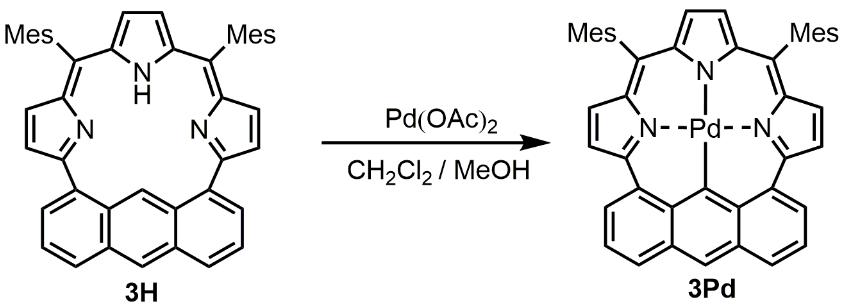

Synthesis of 3Pd: To a solution of **3H** (20 mg, 0.032 mmol) in CH_2Cl_2 (20 mL)/MeOH (4 mL) were added $Pd(OAc)_2$ (43 mg, 0.19 mmol) and NaOAc (19 mg, 0.23 mmol). This mixture was stirred at 25 °C for 2 h. After the solvent was evaporated in vacuo, the mixture was passed through a short silica-gel column with CH_2Cl_2 as an eluent. After the solvent was evaporated in vacuo, recrystallization from CH_2Cl_2/*n*-hexane gave **3Pd** (21 mg, 0.029 mmol, 91% yield) as green solids.

3Pd: ^{1}H NMR (500 MHz, $CDCl_3$) δ = 9.10 (d, *J* = 6.0 Hz, 2H, An-H), 9.01 (s, 1H, An-H), 8.64 (d, *J* = 7.5 Hz, 2H, An-H), 7.89 (m, 2H, An-H), 7.79 (d, *J* = 5.0 Hz, 2H, Py-H), 7.20 (d, *J* = 5.0 Hz, 2H, Py-H), 7.02 (s, 4H, Ar-H), 6.78 (s, 2H, Py-H), 2.43 (s, 6H, Me-H), and 2.13 (s, 12H, Me-H) ppm. ^{13}C NMR (126 MHz, $CDCl_3$) δ = 156.1, 142.8, 140.5, 138.4, 137.5, 137.3, 135.7, 135.3, 134.3, 130.9, 130.7, 130.1, 129.4, 127.8, 126.0, 123.8, 123.0, 122.8, 21.2, and 20.5 ppm. λ_{max} (ε [$M^{-1}cm^{-1}$]) = 364 (41000), 450 (44000), 656 (18000), 728 (15000), and 803 (23000) nm. HR-MS (MALDI-TOF-MS): *m/z* = 735.1450, calcd for $(C_{46}H_{35}N_3Pd)^+$ = 735.1877 ($[M]^+$).

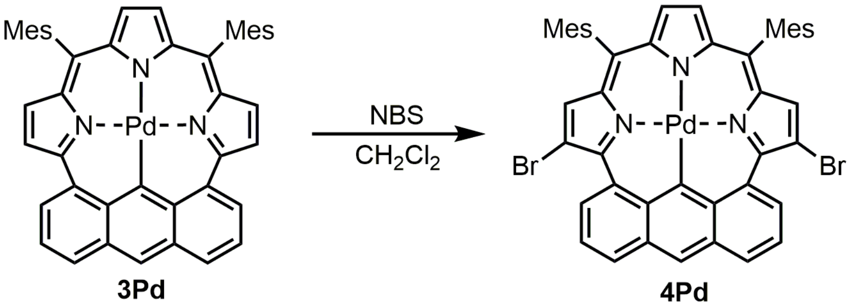

Synthesis of 4Pd: To a solution of **4Pd** (85 mg, 0.12 mmol) in CH_2Cl_2 (30 mL) was added NBS (85.4 mg, 0.24 mmol) at 25 °C. The resulting mixture was stirred at 25 °C for 30 min. After the consumption of **3Pd** was confirmed by TLC, the reaction mixture was poured to water and the products were extracted with $CHCl_3$. The organic extracts were combined, washed with water, and dried over anhydrous sodium sulfate. Separation by silica-gel chromatography (CH_2Cl_2/*n*-hexane as an eluent) and recrystallization from CH_2Cl_2/MeOH gave **4Pd** (97 mg, 0.11 mmol, 92% yield) as yellow-green solids.

4Pd: ^{1}H NMR (500 MHz, $CDCl_3$) δ = 9.43 (d, J = 7.0 Hz, 2H, An-H), 8.83 (s, 1H, An-H), 8.48 (d, J = 8.0 Hz, 2H, An-H), 7.75 (m, 2H, An-H), 7.22 (s, 2H, Py-H), 7.01 (s, 4H, Ar-H), 6.69 (s, 2H, Py-H), 2.42 (s, 6H, Me-H), and 2.14 (s, 12H, Me-H) ppm. ^{13}C NMR (126 MHz, $CDCl_3$) δ = 152.2, 143.6, 140.0, 139.5, 137.9, 137.7, 137.1, 135.2, 134.3, 131.2, 130.4, 129.2, 128.0, 127.9, 127.0, 122.9, 121.1, 111.0, 21.2, and 20.5 ppm. IR (KBr disk): ν = 3427, 2922, 2854, 1578, 1552, 1444, 1311, 1250, 1066, 995, 833, 725 cm^{-1}. HR-MS (MALDI-TOF-MS): m/z = 890.9596, calcd for $(C_{46}H_{33}Br_2N_3Pd)^+$ = 891.0076 ($[M]^+$).

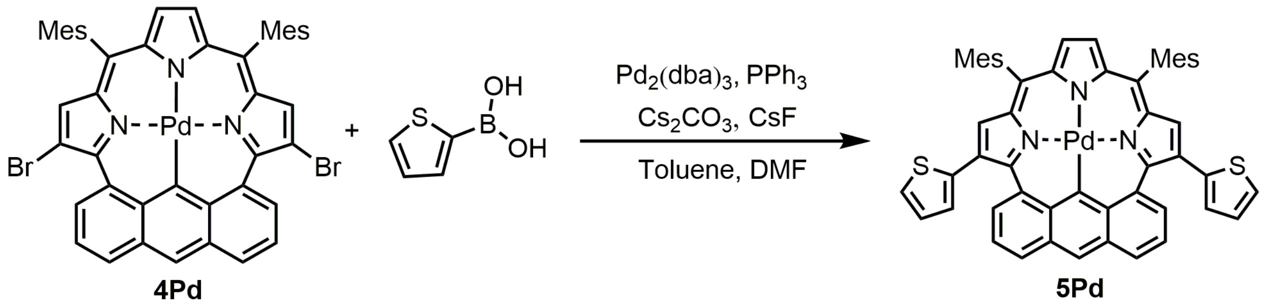

Synthesis of 5Pd: A solution of **4Pd** (45.0 mg, 0.05 mmol), 2-thiopheneboronic acid (32.0 mg, 0.25 mmol), $Pd_2(dba)_3$ (4.6 mg, 0.005 mmol), PPh_3 (5.2 mg, 0.02 mmol), Cs_2CO_3 (32.6 mg, 0.1 mmol) and CsF (15.2 mg, 0.1 mmol) in a mixture of toluene–DMF solution (4 mL/2 mL) was degassed through three freeze-pump-thaw cycles, and the reaction flask was purged with argon. The resulting mixture was heated at 115 °C for 48 h. The reaction mixture was poured to water and the products were extracted with $CHCl_3$. The organic extracts were combined, washed with water, and dried over anhydrous sodium sulfate. The solvent was evaporated in vacuo. The product was purified by column chromatography on silica-gel (CH_2Cl_2/*n*-hexane as an eluent) and recrystallization from CH_2Cl_2/MeOH gave **5Pd** (29 mg, 0.032 mmol, 64% yield) as yellow-green solids.

5Pd: ^{1}H NMR (500 MHz, $CDCl_3$) δ = 8.88 (s, 1H, An-H), 8.54 (dd, J = 7.0 Hz, 1.0 Hz, 2H, An-H), 8.47 (d, J = 7.5 Hz, 2H, An-H), 7.47 (m, 2H, An-H), 7.32 (dd, J = 5.0 Hz, 1.0 Hz, 2H, thiophene-H), 7.12 (s, 2H, Py-H), 7.03 (dd, J = 5.0Hz, 3.5 Hz, 2H, thiophene-H), 7.00 (s, 4H, Ar-H), 6.76 (dd, J = 3.5 Hz, 1.0 Hz, 2H, thiophene-H), 6.71 (s, 2H, Py-H), 2.40 (s, 6H, Me-H), and 2.17 (s, 12H, Me-H) ppm. ^{13}C NMR (126 MHz, $CDCl_3$) δ = 155.3, 143.7, 139.2, 139.1, 138.6, 138.3, 137.6, 137.2, 135.6, 134.9, 134.7, 132.1, 131.2, 129.8, 129.2, 128.3, 127.9, 127.8, 127.3, 125.8, 122.5, 121.9, 100.0, 21.2, and 20.6 ppm. HR-MS (MALDI-TOF-MS): m/z = 899.1508, calcd for $(C_{54}H_{39}N_3PdS_2)^+$ = 899.1632 ($[M]^+$).

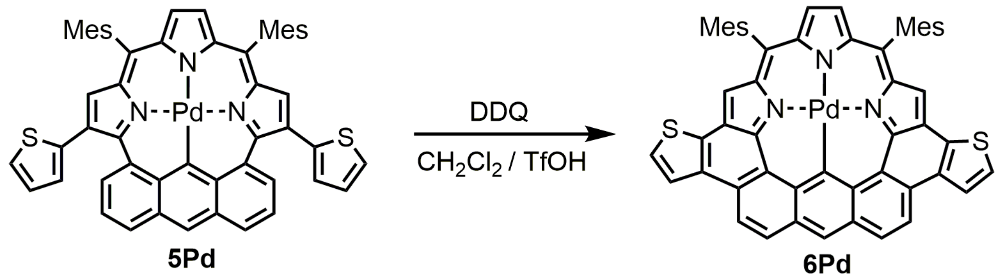

Synthesis of 6Pd: To a solution of **5Pd** (10.0 mg, 0.011 mmol) in dry degassed dichloromethane (3 mL), were added triflic acid (0.03 mL) and DDQ (7.5 mg, 0.033 mmol) dissolved in dry degassed dichloromethane (1 mL). The resulting mixture was stirred for 5 min at 0 °C. The reaction was quenched by addition of a saturated $NaHCO_3$ solution and the resulting mixture was extracted with CH_2Cl_2 and the organic layer was dried over anhydrous Na_2SO_4. The solvent was evaporated and the product was separated by column chromatography on silica gel (CH_2Cl_2/*n*-hexane as an eluent) and recrystallization from CH_2Cl_2/MeOH gave **6Pd** (3.0 mg, 0.003 mmol, 30% yield) as brown solids.

6Pd: ^{1}H NMR (500 MHz, $CDCl_3$) δ = 7.82 (s, 1H, An-H), 7.67 (d, J = 8.5 Hz, 2H, An-H), 7.53 (d, J = 8.5 Hz, 2H, An-H), 7.24 (d, J = 5.0 Hz, 2H, thiophene-H), 7.03 (d, J = 5.0 Hz, 2H, thiophene-H), 6.92 (s, 4H, Ar-H), 6.18 (s, 2H, Py-H), 5.93 (s, 2H, Py-H), 2.36 (s, 6H, Me-H), and 2.23 (s, 12H, Me-H) ppm. λ_{max} (ε [$M^{-1}cm^{-1}$]) = 338 (47000), 405 (290000), 483 (27000), 630 (13000), 718 (12000), 791(17000), 907(6600), 1039(5400), and 1209(4600) nm. IR (KBr disk): ν = 3433, 2924, 2852, 1585, 1529, 1464, 1352, 1300, 1236, 993, 957, 851, 808, 713 cm^{-1}. HR-MS (MALDI-TOF-MS): *m/z* = 896.1476, calcd for $(C_{54}H_{36}N_3PdS_2)^+$ = 896.1397 ($[M+H]^+$).

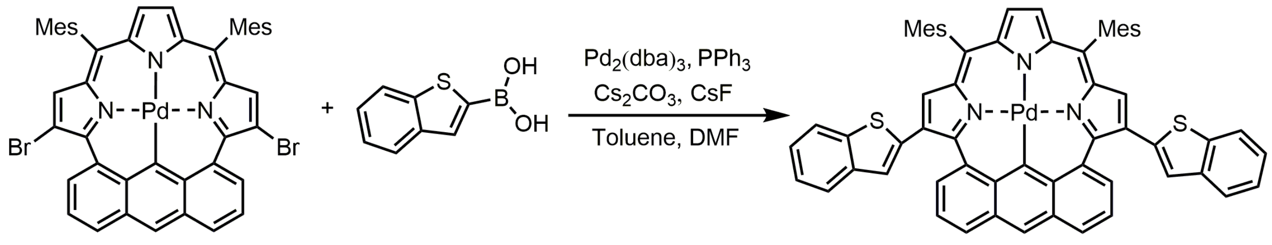

Synthesis of 7Pd: A solution of **4Pd** (60.0 mg, 0.067 mmol), benzothien-2-ylboronic acid (60.5mg, 0.34 mmol), $Pd_2(dba)_3$ (6.1 mg, 0.0067 mmol), PPh_3 (7.1 mg, 0.027 mmol), Cs_2CO_3 (44 mg, 0.13 mmol) and CsF (20.4 mg, 0.13 mmol) in a mixture of toluene–DMF solution (6 mL/3 mL) was degassed through three freeze-pump-thaw cycles, and the reaction flask was purged with argon. The resulting mixture was heated at 115 °C for 48 h. The reaction mixture was poured to water and the products were extracted with $CHCl_3$. The

organic extracts were combined, washed with water, and dried over anhydrous sodium sulfate. The solvent was evaporated in vacuo. The product was separated by column chromatography on silica-gel (CH_2Cl_2/*n*-hexane as an eluent) and recrystallization from CH_2Cl_2/MeOH gave **7Pd** (35 mg, 0.035 mmol, 52% yield) as yellow-green solids.

7Pd: ^{1}H NMR (500 MHz, $CDCl_3$) δ = 8.93 (s, 1H, An-H), 8.71 (dd, *J* = 7.0Hz, 1.0 Hz, 2H, An-H), 8.51 (d, *J* = 7.5 Hz, 2H, An-H), 7.79 (d, *J* = 8.0 Hz, 2H, Benzothiophene-H), 7.73 (d, *J* = 7.5 Hz, 2H, Benzothiophene-H), 7.47 (m, 2H, An-H), 7.38~7.31 (m, 4H, Benzothiophene-H), 7.22 (s, 2H, Benzothiophene-H), 7.05 (s, 2H, Py-H), 7.01 (s, 4H, Ar-H), 6.74 (s, 2H, Py-H), 2.40 (s, 6H, Me-H), and 2.18 (s, 12H, Me-H) ppm. ^{13}C NMR (126 MHz, $CDCl_3$) δ = 155.0, 143.9, 140.5, 140.0, 139.8, 139.7, 139.2, 138.4, 137.8, 137.2, 136.1, 135.0, 134.8, 132.4, 131.3, 130.0, 129.2, 128.1, 127.9, 126.7, 124.4, 124.2, 123.6, 123.4, 122.7, 122.0, 121.9, 21.2, and 20.6 ppm. HR-MS (MALDI-TOF-MS): *m/z* = 999.1914, calcd for $(C_{62}H_{43}N_3PdS_2)^+$ = 999.1947 ($[M]^+$).

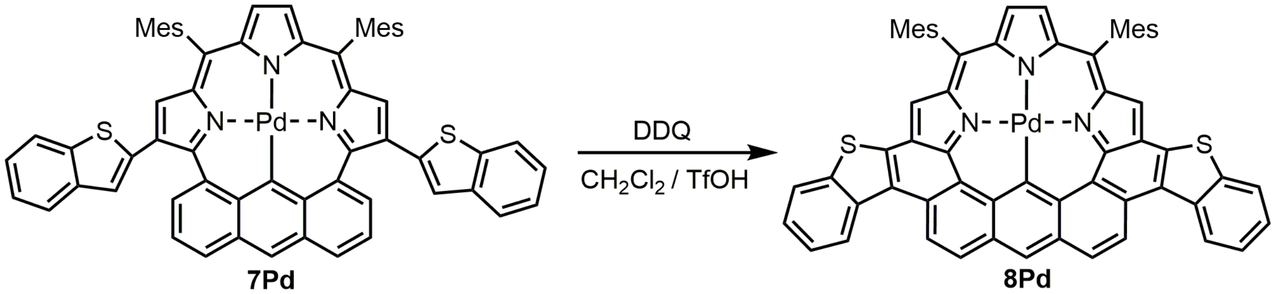

Synthesis of 8Pd: To a solution of **7Pd** (19 mg, 0.0191 mmol) in dry degassed dichloromethane (10 mL), triflic acid (0.1 mL) and DDQ (12.9 mg, 0.057 mmol) dissolved in dry degassed dichloromethane (2 mL) were added. The resulting mixture was stirred for 5 min at 0°C. The reaction was quenched by addition of a saturated $NaHCO_3$ solution and the resulting mixture was extracted with CH_2Cl_2 and the organic layer was dried over anhydrous Na_2SO_4. The solvent was evaporated and the product was isolated by column chromatography on silica gel (CH_2Cl_2/*n*-hexane as an eluent) and recrystallization from CH_2Cl_2/MeOH gave **8Pd** (5.0 mg, 0.005 mmol, 26% yield) as brown solids.

8Pd: ^{1}H NMR (500 MHz, $CDCl_3$) δ = 8.26 (d, *J* = 8.0 Hz, 2H, An-H), 8.10 (d, *J* = 8.5 Hz, 2H, An-H), 7.77 (s, 1H, An-H), 7.68 (m, 4H, Benzothiophene-H), 7.38 (m, 2H, Benzothiophene-H), 7.28 (m, 2H, Benzothiophene-H), 6.92 (s, 4H, Ar-H), 6.20 (s, 2H, Py-H), 5.93 (s, 2H, Py-H), 2.37 (s, 6H, Me-H), and 2.24 (s, 12H, Me-H) ppm. λ_{max} (ε [$M^{-1}cm^{-1}$]) = 346 (55000), 416 (29000), 485 (22000), 551 (12000), 583 (13000),

640(12000), 816(20000), 1067(5000), and 1249(3100) nm. IR (KBr disk): ν = 3427, 2920, 2846, 1516, 1466, 1352, 1232, 997, 960, 847, 806 cm^{-1}. HR-MS (MALDI-TOF-MS): *m/z* = 996.1682, calcd for $(C_{62}H_{40}N_3PdS_2)^+$ = 996.1712 ($[M+H]^+$).

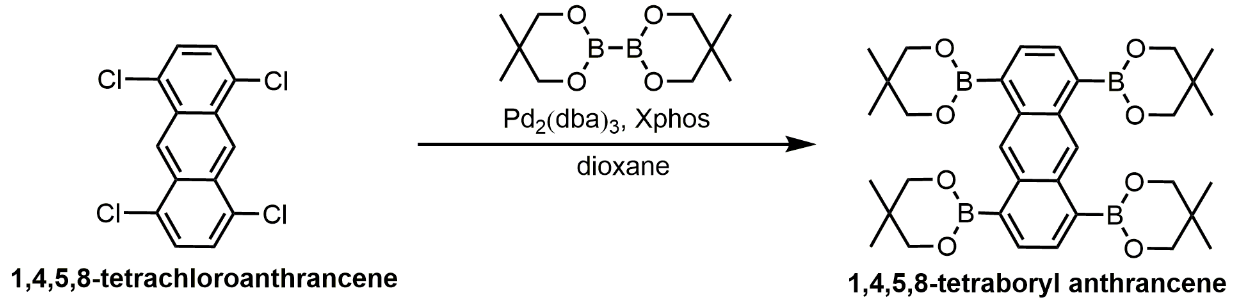

Synthesis of 1,4,5,8-tetraborylanthracene: A solution of 1,4,5,8-tetrachloroanthracene (316 mg, 1.0 mmol), bis(neopentylglycolato)diboron (1.13 g, 5.0 mmol), $Pd_2(dba)_3$ (55.0 mg, 0.06 mmol), Xphos (114.4 mg, 0.24 mmol), NaOAc (2.05 g, 25.0 mmol) in dioxane (20 mL) was degassed through three freeze-pump-thaw cycles, and the reaction flask was purged with argon. The resulting mixture was heated at 90 °C for 48 h. The reaction mixture was poured to water and the products were extracted with $CHCl_3$. The organic layer was washed with brine, dried over anhydrous Na_2SO_4 and evaporated in vacuo. The residue was recrystallized with hexane to give 1,4,5,8-tetraboryl anthracene (576 mg, 0.92 mmol, 92% yield) as white solids.

1,4,5,8-tetraboryl anthracene: ^1H NMR (500 MHz, $CDCl_3$) δ = 10.11 (s, 2H, An-H), 7.97 (s, 4H, An-H), 3.95 (s, 16H, $-CH_2-$), and 1.15 (s, 24H, Me-H). ^{13}C NMR (126 MHz, $CDCl_3$) δ = 133.9, 133.2, 127.9, 72.6, 31.9, 22.2, and 22.1 ppm. HR-MS (MALDI-TOF-MS): *m/z* = 626.3550, calcd for $(C_{34}H_{46}B_4O_8)^+$ = 626.3581 ($[M]^+$).

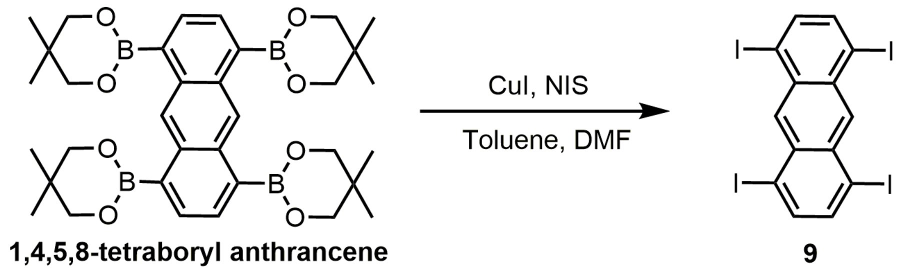

Synthesis of 9: A solution of 1,4,5,8-tetraborylanthracene (680 mg, 1.08 mmol), CuI (1.60 g, 8.64 mmol), NIS (1.94 g, 8.64 mmol) in DMF/toluene solution (140 / 70 mL) was heated at 80 °C for 12 h. The reaction mixture was poured to water and the products were extracted with $CHCl_3$. The organic layer was washed with brine, dried over anhydrous Na_2SO_4 and evaporated in vacuo. The residue was purification by column

chromatography (CH_2Cl_2 as eluent) and recrystallization with hexane gave **9** (184.1 mg, 0.27 mmol, 25% yield) as white solids.

9: ^{1}H NMR (500 MHz, $CDCl_3$) δ = 8.96 (s, 2H, An-H) and 7.87 (s, 4H, An-H). HR-MS (APCI-MS): *m/z* = 681.6658, calcd for $(C_{14}H_6I_4)^-$ = 681.6643 ($[M]^-$).

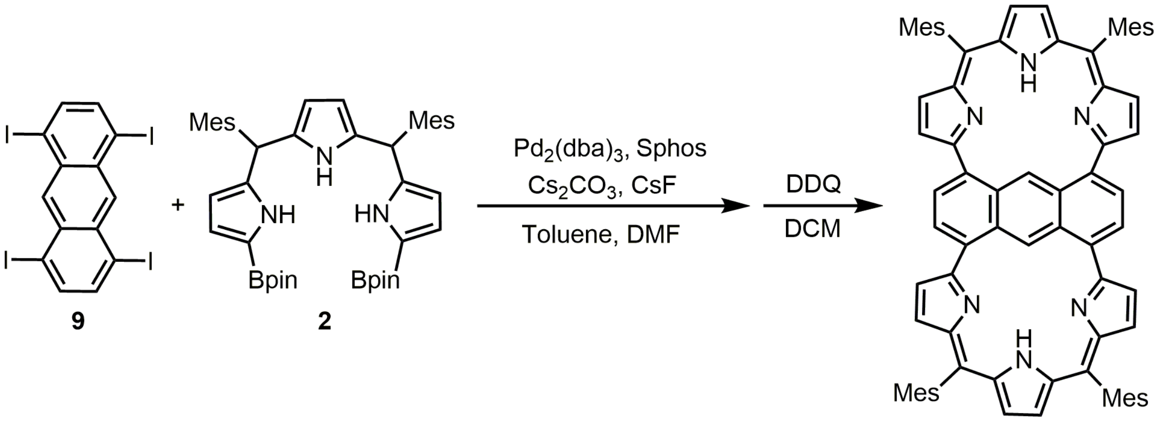

Synthesis of 10H: A solution of 1,4,5,8-tetraiodoanthracene **9** (177.3 mg, 0.26 mmol), diboryltripyrrane **2** (556.6 mg, 0.78 mmol), $Pd_2(dba)_3$ (47.6 mg, 0.052 mmol), Sphos (85.3 mg, 0.208 mmol), Cs_2CO_3 (338.0 mg, 1.04 mmol), and CsF (157.0 mg, 1.04 mmol) in a mixture of toluene–DMF (16 mL/8 mL) was degassed through three freeze-pump- thaw cycles, and the reaction flask was purged with argon. The resulting mixture was heated at 115 °C for 48 h. The reaction mixture was poured to water and the product was extracted with $CHCl_3$. The organic extracts were combined, washed with water, and dried over anhydrous sodium sulfate. The solvent was evaporated in vacuo. To the resulting mixture was added a solution of DDQ (236.1 mg, 1.04 mmol) in CH_2Cl_2 (20 mL). After being stirred for 10 min, the mixture was passed through a short silica-gel and an Al_2O_3 column (CH_2Cl_2 as an eluent). Evaporation of the solvent followed by silica-gel column chromatography (CH_2Cl_2/*n*-hexane as an eluent) and recrystallization from CH_2Cl_2/MeOH gave **10H** (29.0 mg, 0.026 mmol, 10% yield) as green solids.

10H: ^{1}H NMR (500 MHz, $CDCl_3$) δ = 14.61 (s, 2H, N-H), 12.78 (s, 2H, An-H), 7.91 (s, 4H, An-H), 6.95~6.94 (m, 12H, Py-H and Ar-H), 6.64 (d, J = 4.5 Hz, 4H, Py-H), 5.92 (d, J = 2.0 Hz, 4H, Py-H), 2.36 (s, 12H, Me-H), and 2.20 (s, 24H, Me-H) ppm. ^{13}C NMR (126 MHz, $CDCl_3$) δ = 171.1, 154.5, 139.5, 137.5, 137.1, 136.8, 134.6, 134.5, 134.1, 129.4, 129.3, 127.9, 126.5, 125.9, 121.0, 21.1, and 20.2 ppm. λ_{max} (ε [$M^{-1}cm^{-1}$]) = 326 (44000), 358 (48000), 431 (79000), 598 (32000), and 644 (28000) nm. IR (KBr disk): ν = 3433, 3300, 2922, 2854, 1595, 1570, 1385, 1260, 1182, 1050, 910, 802, 781 cm^{-1}. Fluorescence (CH_2Cl_2, λ_{ex} = 418

nm): λ_{max} = 652 nm, ϕ_F = 0.1%. HR-MS (MALDI-TOF-MS): *m/z* = 1084.5249, calcd for $(C_{78}H_{64}N_6)^+$ = 1084.5187 ($[M]^+$).

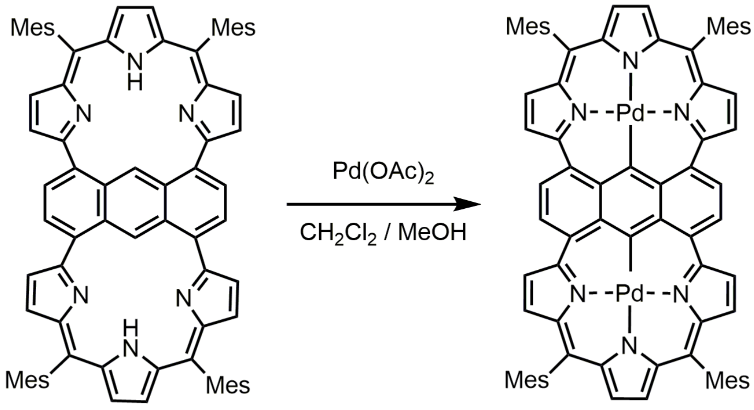

10H **10Pd**

Synthesis of 10Pd: To a solution of **10H** (31.9 mg, 0.029 mmol) in CH_2Cl_2 (20 mL)/MeOH (4 mL) were added $Pd(OAc)_2$ (78.6 mg, 0.35 mmol) and NaOAc (33.7 mg, 0.41 mmol). The resulting solution was stirred at 25 °C for 2 h. After the solvent was evaporated in vacuo, the mixture was passed through a short silica-gel column (CH_2Cl_2 as an eluent). The solvent was evaporated in vacuo. The product was recrystallized from CH_2Cl_2/*n*-hexane to give **10Pd** (29 mg, 0.022 mmol, 76% yield) as brown solids.

10Pd: ^{1}H NMR (500 MHz, $CDCl_3$) δ = 6.95 (s, 4H, An-H), 6.77 (s, 4H, Ar-H), 6.74 (s, 4H, Ar-H), 6.21 (d, *J* = 4.5 Hz, 4H, Py-H), 6.07 (d, *J* = 5.0 Hz, 4H, Py-H), 5.58 (s, 4H, Py-H), 2.78 (s, 12H, Me-H), 2.29 (s, 12H, Me-H), and 1.59 (s, 12H, Me-H) ppm. ^{13}C NMR (126 MHz, $CDCl_3$) δ = 160.7, 147.4, 145.5, 144.2, 143.9, 142.1, 137.4, 136.8, 136.3, 134.7, 133.6, 127.8, 126.0, 124.8, 124.3, 121.6, 21.1, 20.6, and 19.3 ppm. λ_{max} (ε [$M^{-1}cm^{-1}$]) = 362 (35000), 502 (82000), 625 (13000), 837 (19000), and 926 (25000) nm. IR (KBr disk): ν = 3427, 2922, 2854, 1591, 1556, 1352, 1300, 1254, 999, 831, 727 cm^{-1}. HR-MS (MALDI-TOF-MS): *m/z* = 1292.2980, calcd for $(C_{78}H_{60}N_6Pd_2)^+$ = 1292.2978 ($[M]^+$).

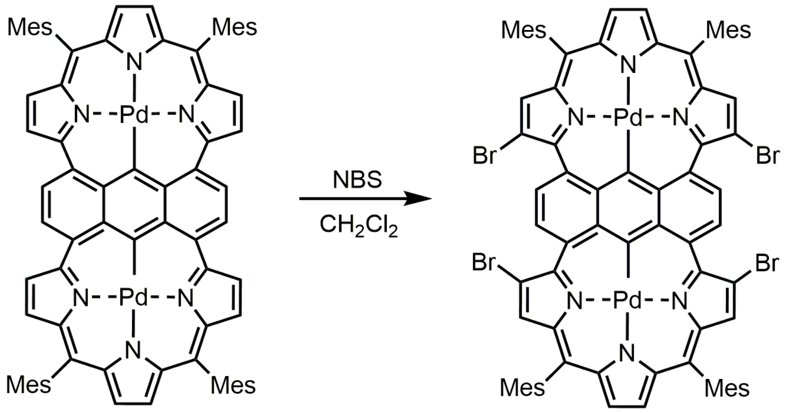

10Pd **11Pd**

Synthesis of 11Pd: To a solution of **10Pd** (30.0 mg, 0.023 mmol) in CH_2Cl_2 (20 mL) was added NBS (16 mg, 0.092 mmol) at 25 °C. The resulting mixture was stirred at 25 °C for ca. 30 min. After the consumption of **11Pd** was confirmed by TLC, the reaction mixture was poured to water and the product was extracted with $CHCl_3$. The organic extracts were combined, washed with water, and dried over anhydrous sodium sulfate. Evaporation of the solvent followed by silica-gel chromatography (CH_2Cl_2/*n*-hexane as an eluent) and recrystallization from CH_2Cl_2/MeOH gave **11Pd** (15 mg, 0.0096 mmol, 42% yield) as brown solids.

11Pd: ^{1}H NMR (500 MHz, $CDCl_3$) δ = 7.46 (s, 4H, An-H), 6.96 (s, 4H, Ar-H), 6.78 (s, 4H, Ar-H), 6.39 (s, 4H, Py-H), 5.74 (s, 4H, Py-H), 2.69 (s, 12H, Me-H), 2.31 (s, 12H, Me-H), and 1.66 (s, 12H, Me-H) ppm. ^{13}C NMR (126 MHz, $CDCl_3$) δ = 156.4, 145.7, 144.8, 143.9, 140.8, 140.2, 137.91, 137.86, 136.7, 136.3, 133.0, 128.03, 127.98, 127.5, 125.6, 123.1, 111.2, 21.1, 20.6, and 19.5 ppm. HR-MS (MALDI-TOF-MS): *m/z* = 1603.9386, calcd for $(C_{78}H_{56}Br_4N_6Pd_2)^+$ = 1603.9377 ($[M]^+$).

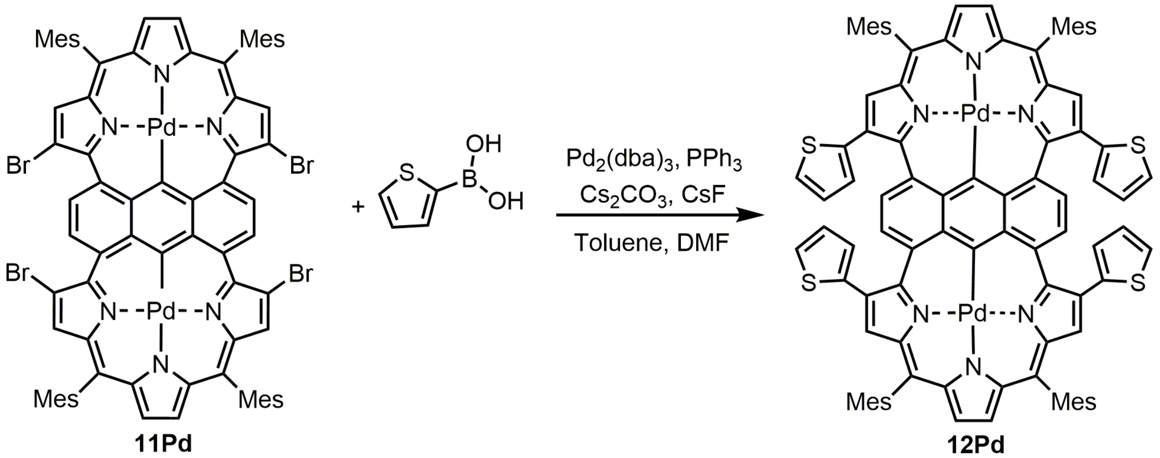

Synthesis of 12Pd: A solution of **11Pd** (20.0 mg, 0.012 mmol), 2-thiopheneboronic acid (15.4 mg, 0.12 mmol), $Pd_2(dba)_3$ (2.2 mg, 2.4 μmmol), PPh_3 (2.5 mg, 9.6 μmmol), Cs_2CO_3 (15.6 mg, 0.048 mmol) and CsF (7.2 mg, 0.048 mmol) in a mixture of toluene–DMF (4 mL/2 mL) was degassed through three freeze-pump-thaw cycles, and the reaction flask was purged with argon. The resulting mixture was heated at 115 °C for 48 h. The reaction mixture was poured to water and the product was extracted with $CHCl_3$. The organic extracts were combined, washed with water, and dried over anhydrous sodium sulfate. The solvent was evaporated in vacuo. The product was separated by column chromatography on silica-gel (CH_2Cl_2/*n*-hexane as an eluent). Recrystallization from CH_2Cl_2/MeOH gave **12Pd** (7.2 mg, 0.0043 mmol, 36% yield) as brown solids.

12Pd: ^{1}H NMR (500 MHz, $CDCl_3$) δ = 7.12 (d, J = 5.0 Hz, 4H, thiophene-H), 6.95 (s, 4H, Ar-H), 6.86~6.83 (m, 4H, thiophene-H), 6.81 (s, 4H, Ar-H), 6.74 (d, J = 3.3 Hz, 4H, thiophene-H), 6.32 (s, 4H, An-H), 6.27 (s, 4H, Py-H), 5.74 (s, 4H, Py-H), 2.73 (s, 12H, Me-H), 2.31 (s, 12H, Me-H), and 1.73 (s, 12H, Me-H) ppm. ^{13}C NMR (126 MHz, $CDCl_3$) δ = 159.5, 146.0, 144.7, 143.3, 141.2, 140.9, 137.6, 136.9, 136.7, 136.4, 134.4, 133.6, 131.0, 128.0, 127.93, 127.87, 127.2, 127.1, 125.1, 125.0, 123.7, 21.1, 20.6, and 19.6 ppm. HR-MS (MALDI-TOF-MS): m/z = 1620.2491, calcd for $(C_{94}H_{68}N_6Pd_2S_4)^+$ = 1620.2488 ($[M]^+$).

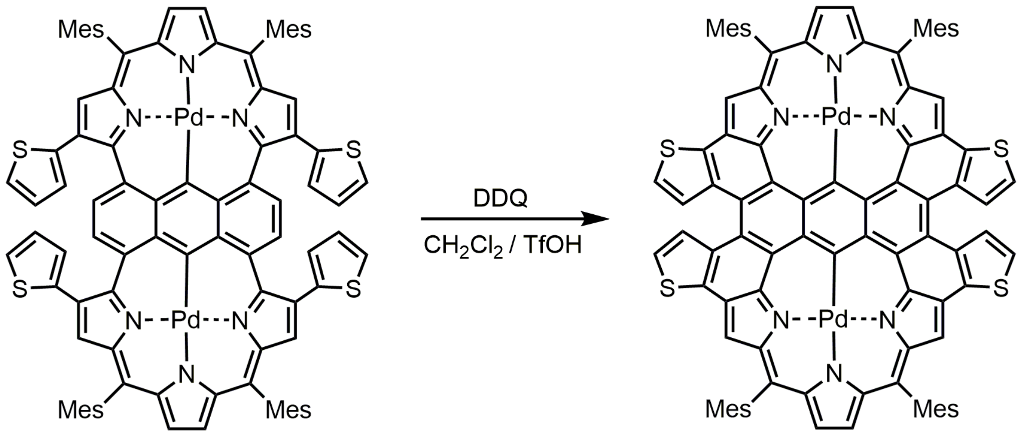

12Pd **13Pd**

Synthesis of 13Pd: To a solution of **12Pd** (10 mg, 6.2 μmol) in dry degassed dichloromethane (5 mL) were added triflic acid (0.05 mL) and a solution of DDQ (5.6 mg, 0.025 mmol) in dry degassed dichloromethane (1 mL). The resulting mixture was stirred for 5 min at 0 °C. The reaction was quenched by addition of a saturated $NaHCO_3$ solution and the resulting mixture was extracted with CH_2Cl_2 and the organic layer was dried over anhydrous Na_2SO_4. The solvent was evaporated and the product was separated by column chromatography on silica gel (CH_2Cl_2/n-hexane as an eluent) and recrystallization from CH_2Cl_2/MeOH gave **13Pd** (3.1 mg, 1.9 μmol, 31% yield) as brown solids.

13Pd: ^{1}H NMR (500 MHz, $CDCl_3$) δ = 6.91 (s, 4H, Ar-H), 6.54 (d, J = 5.0 Hz, 4H, thiophene-H), 6.46 (s, 4H, Ar-H), 6.16 (d, J = 5.0 Hz, 4H, thiophene-H), 4.46 (s, 4H, Py-H), 4.43 (s, 4H, Py-H), 3.30 (s, 12H, Me-H), 2.18 (s,12H, Me-H), and 1.13 (s, 12H, Me-H) ppm. ^{13}C NMR (126 MHz, $CDCl_3$) δ = 154.4, 151.8, 151.1, 150.5, 149.6, 147.5, 137.5, 136.5, 135.3, 134.0, 133.9, 133.5, 133.0, 131.9, 129.2, 127.8, 127.0, 125.2, 123.7, 122.2, 20.9, and 20.6 ppm. λ_{max} (ε [$M^{-1}cm^{-1}$]) = 365 (68000), 481 (62000), 750 (64000), and 1258 (5500) nm. IR (KBr disk): ν = 3435, 2920, 2850, 1550, 1344, 1250, 982, 964, 839,723 cm^{-1}. HR-MS (MALDI-TOF-MS): m/z = 1612.1828, calcd for $(C_{94}H_{60}N_6Pd_2S_4)^+$ = 1612.1861 ($[M]^+$).

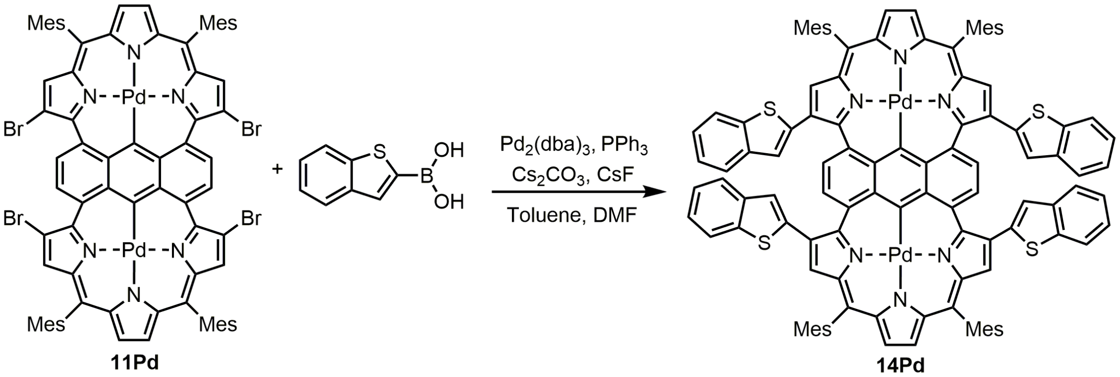

Synthesis of 14Pd: A solution of **11Pd** (58 mg, 0.036 mmol), benzothien-2-ylboronic acid (64.1mg, 0.36 mmol), $Pd_2(dba)_3$ (6.6 mg, 7.2 μmol), PPh_3 (7.6 mg, 28.8 μmol), Cs_2CO_3 (47 mg, 0.14 mmol) and CsF (22 mg, 0.14 mmol) in a mixture of toluene–DMF (6 mL/3 mL) was degassed through three freeze-pump-thaw cycles, and the reaction flask was purged with argon. The resulting mixture was stirred at 115 °C for 48 h. The reaction mixture was poured to water and the products were extracted with $CHCl_3$. The organic extracts were combined, washed with water, and dried over anhydrous sodium sulfate. The solvent was evaporated in vacuo. The product was separated by column chromatography on silica-gel (CH_2Cl_2/*n*-hexane as an eluent). Recrystallization from CH_2Cl_2/MeOH gave **14Pd** (20 mg, 0.011 mmol, 31% yield) as maroon solids.

14Pd: ^{1}H NMR (500 MHz, $CDCl_3$) δ = 7.64~7.62 (m, 8H, benzothiophene-H), 7.24~7.17 (m, 8H, benzothiophene-H), 6.96 (s, 4H, Ar-H), 6.94 (s, 4H, Ar-H), 6.84 (s, 4H, benzothiophene-H), 6.42 (s, 4H, An-H), 6.38 (s, 4H, Py-H), 5.80 (s, 4H, Py-H), 2.78 (s, 12H, Me-H), 2.33 (s, 12H, Me-H), and 1.76 (s, 12H, Me-H) ppm. ^{13}C NMR (126 MHz, $CDCl_3$) δ = 159.4, 146.3, 144.9, 143.8, 141.3, 140.9, 139.9, 139.7, 137.7, 136.92, 136.88, 136.4, 135.3, 133.4, 131.0, 128.2, 128.1, 128.0, 125.4, 124.3, 124.2, 123.8, 123.5, 123.3, 121.8, 21.1, 20.7, and 19.6 ppm. HR-MS (MALDI-TOF-MS): *m/z* =1820.3235, calcd for $(C_{110}H_{76}N_6Pd_2S_4)^+$ = 1820.3118 ($[M]^+$).

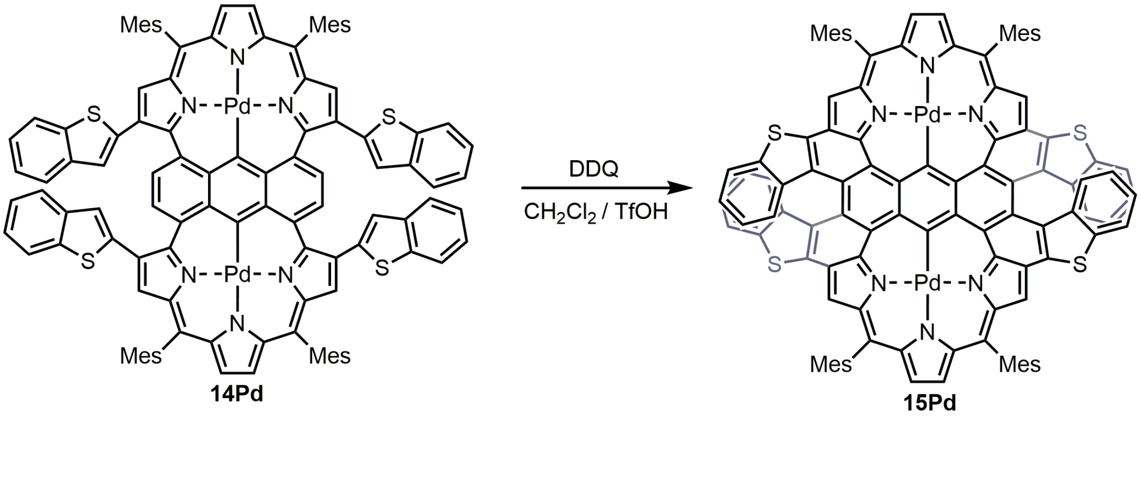

Synthesis of 15Pd: To a solution of **14Pd** (20 mg, 0.01 mmol) in dry degassed dichloromethane (10 mL) were added triflic acid (0.1 mL) and a solution of DDQ (13.6 mg, 0.06 mmol) in dry degassed dichloromethane (2 mL). The resulting mixture was stirred for 5 min at 0 °C. The reaction was quenched by addition of a saturated $NaHCO_3$ solution and the products were extracted with CH_2Cl_2 and the organic layer was dried over anhydrous Na_2SO_4. The solvent was evaporated and the product was separated by column chromatography on silica gel (CH_2Cl_2/*n*-hexane as an eluent) and recrystallization from CH_2Cl_2/MeOH gave **15Pd** (12 mg, 6.6 μmol, 66% yield) as brown solids.

15Pd: ^{1}H NMR (500 MHz, $CDCl_3$) δ = 7.20 (d, J = 8.0 Hz, 4H, Ar-H), 6.94 (s, 4H, Ar-H), 6.78~6.74 (m, 8H, benzothiophene-H), 6.51~6.47 (m, 8H, benzothiophene-H), 4.43 (s, 4H, β-H), 4.42 (s, 4H, β-H), 3.40 (s, 12H, Me-H), 2.20 (s, 12H, Me-H), and 1.24 (s, 12H, Me-H) ppm. ^{13}C NMR (126 MHz, $CDCl_3$) δ = 154.2, 153.5, 152.9, 152.4, 151.4, 150.8, 138.0, 137.7, 136.5, 136.1, 135.5, 135.4, 134.4, 131.7, 129.6, 128.9, 128.0, 127.9, 125.6, 124.9, 124.2, 124.1, 123.0, 121.8, 20.9, 20.7 and 19.1 ppm. λ_{max} (ε [$M^{-1}cm^{-1}$]) = 314 (66000), 383 (85000), 483 (60000), 781 (72000), and 1378 (5100) nm. IR (KBr disk): ν = 3419, 2918, 2852, 1544, 1506, 1342, 1246, 976, 849, 727 cm^{-1}. HR-MS (MALDI-TOF-MS): *m/z* = 1812.2484, calcd for $(C_{110}H_{68}N_6Pd_2S_4)^+$ = 1812.2492 ($[M]^+$).

Spectra of Compounds

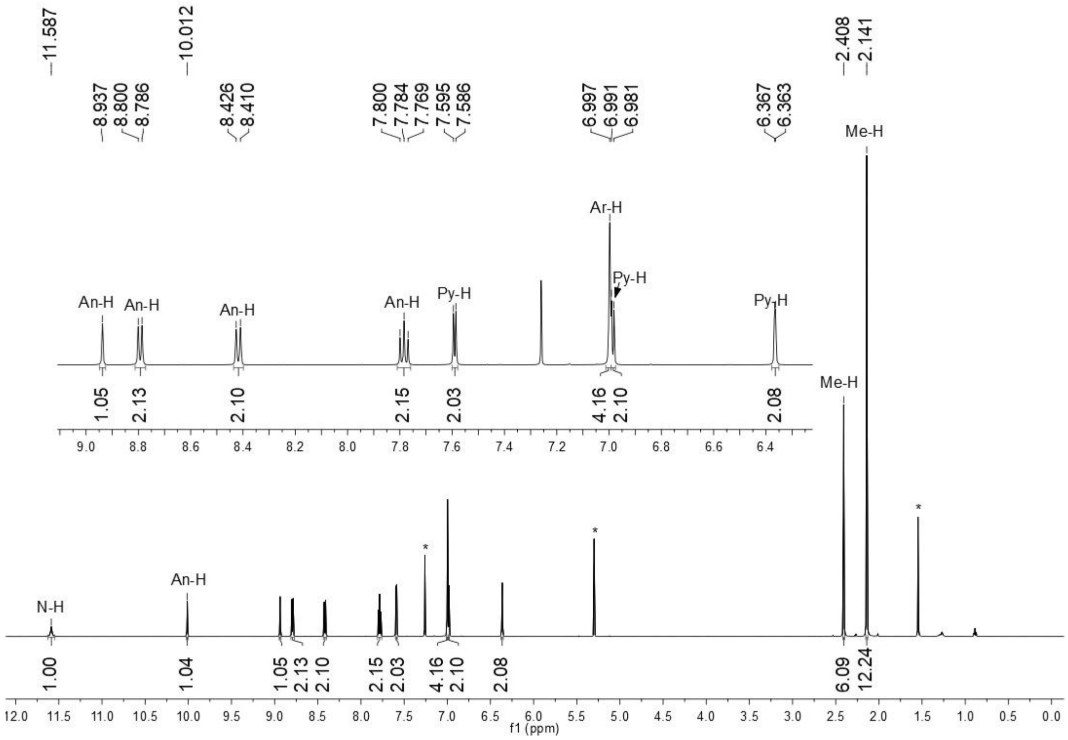

Figure S1. ^{1}H NMR spectrum of **3H** in $CDCl_3$. * Solvent or impurities

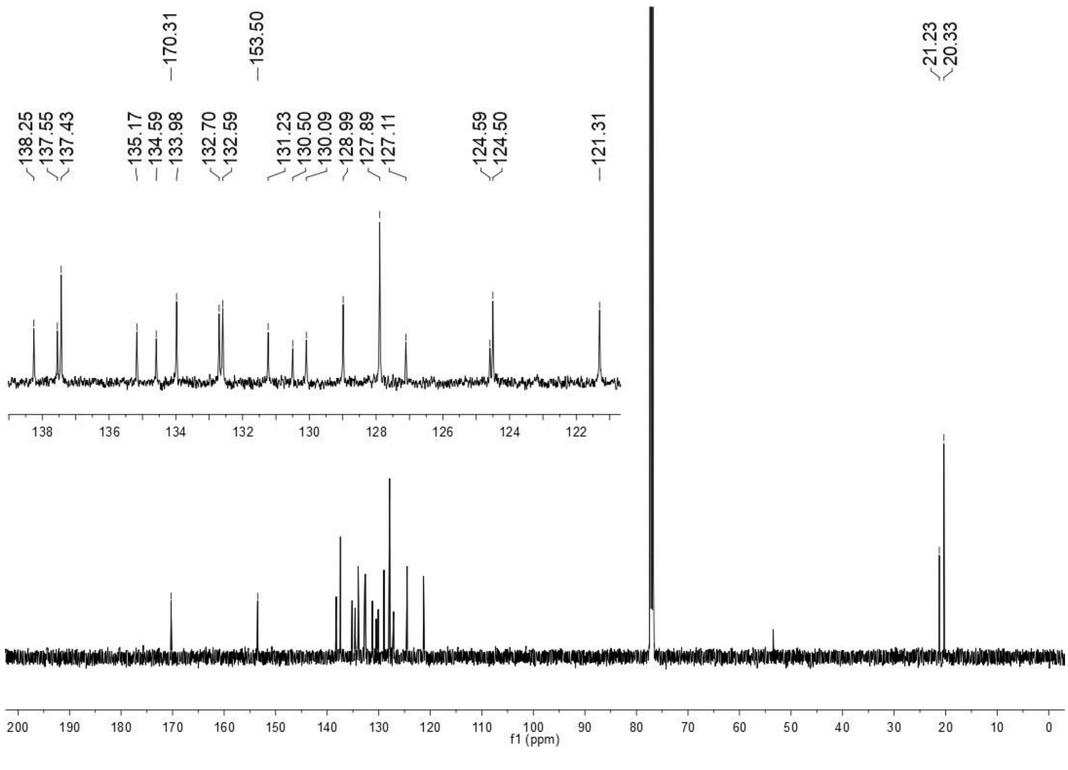

Figure S2. ^{1}C NMR spectrum of **3H** in $CDCl_3$.

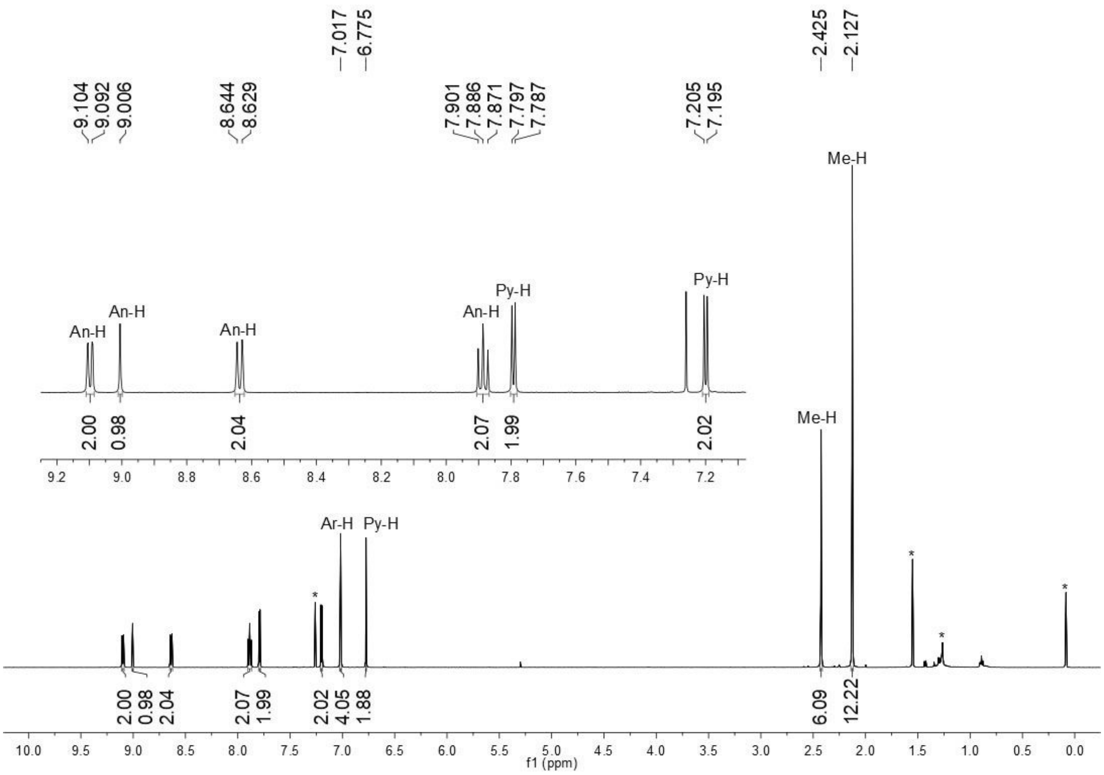

Figure S3. ^{1}H NMR spectrum of **3Pd** in $CDCl_3$. * Solvent or impurities

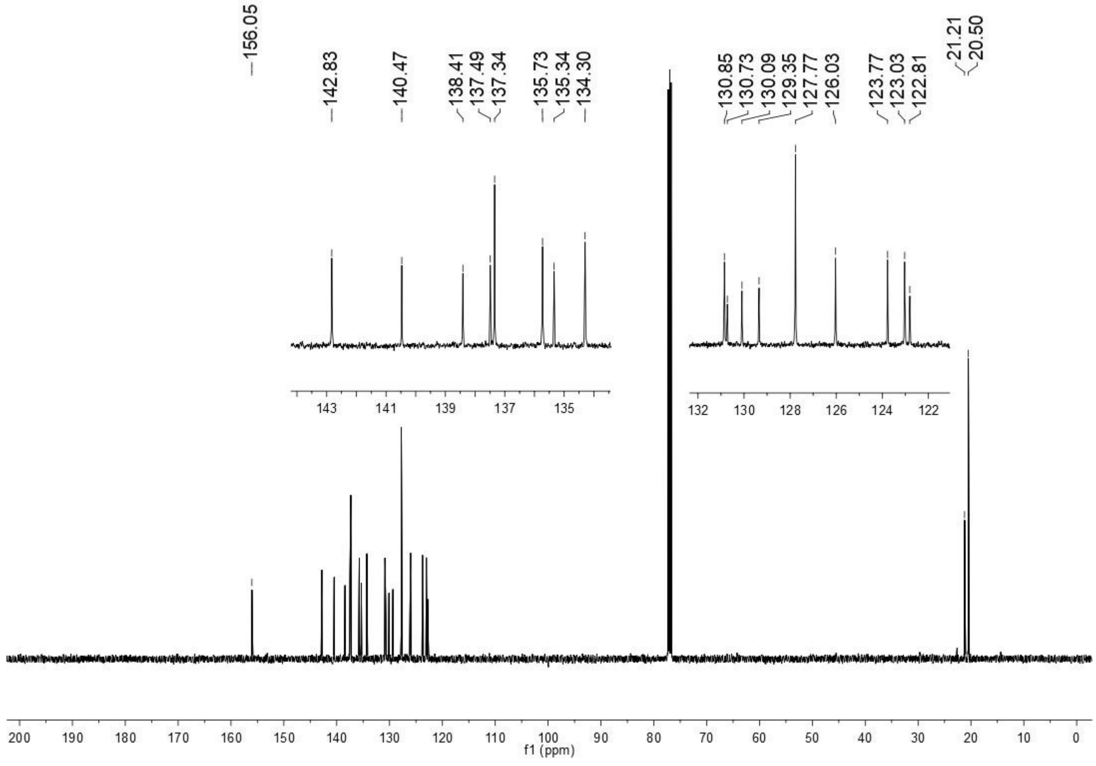

Figure S4. ^{13}C NMR spectrum of **3Pd** in $CDCl_3$.

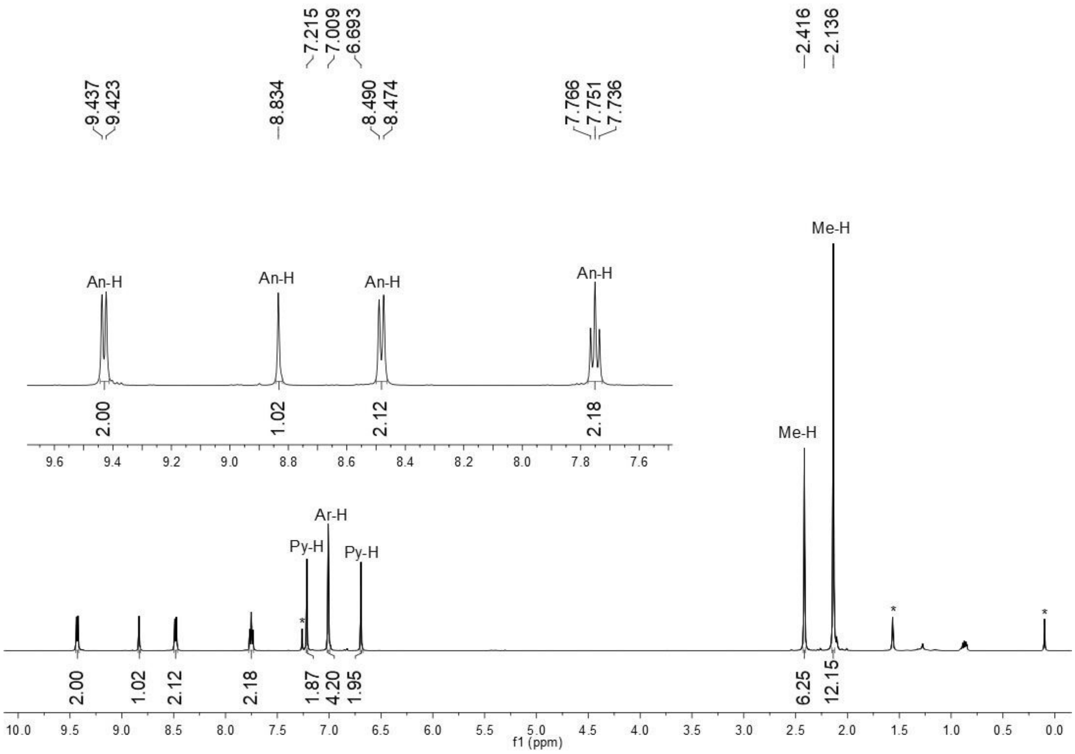

Figure S5. ^{1}H NMR spectrum of **4Pd** in $CDCl_3$. * Solvent or impurities

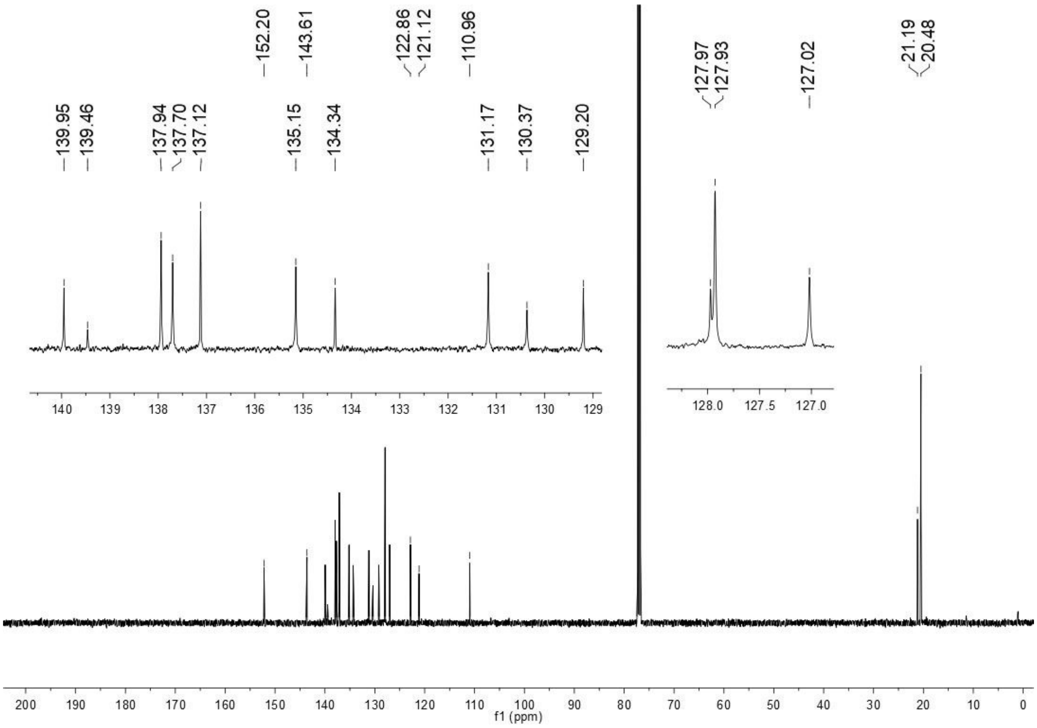

Figure S6. ^{1}C NMR spectrum of **4Pd** in $CDCl_3$.

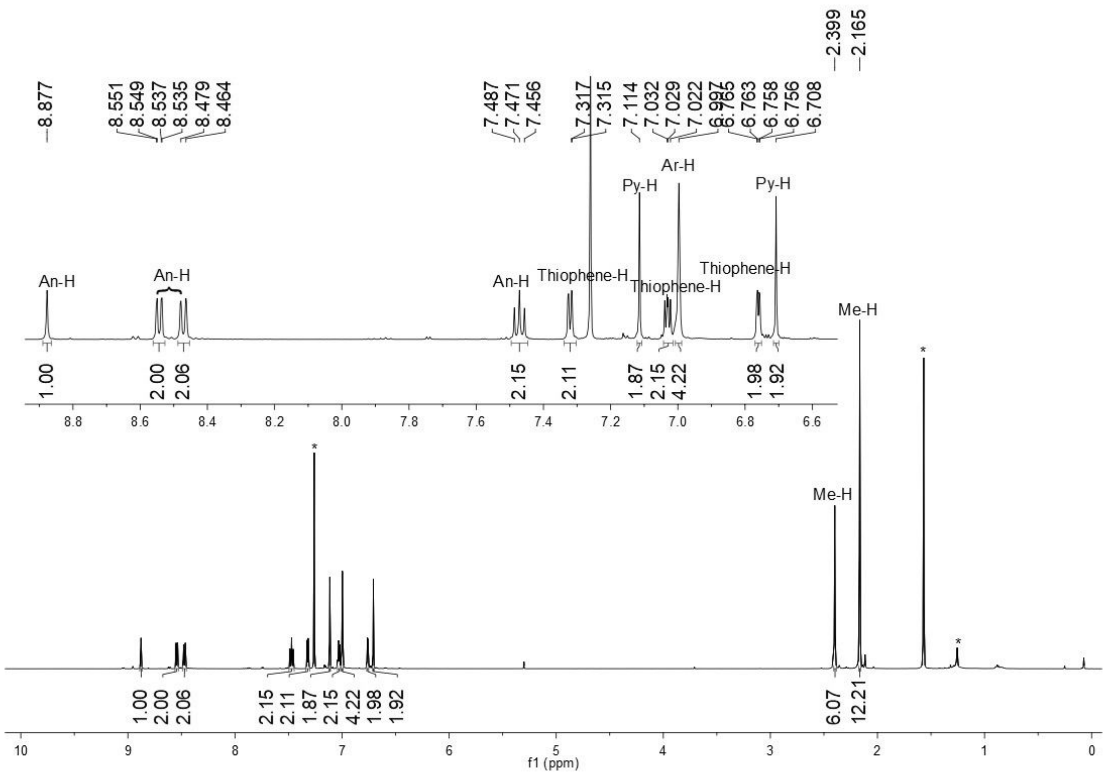

Figure S7. ^{1}H NMR spectrum of **5Pd** in $CDCl_3$. * Solvent or impurities

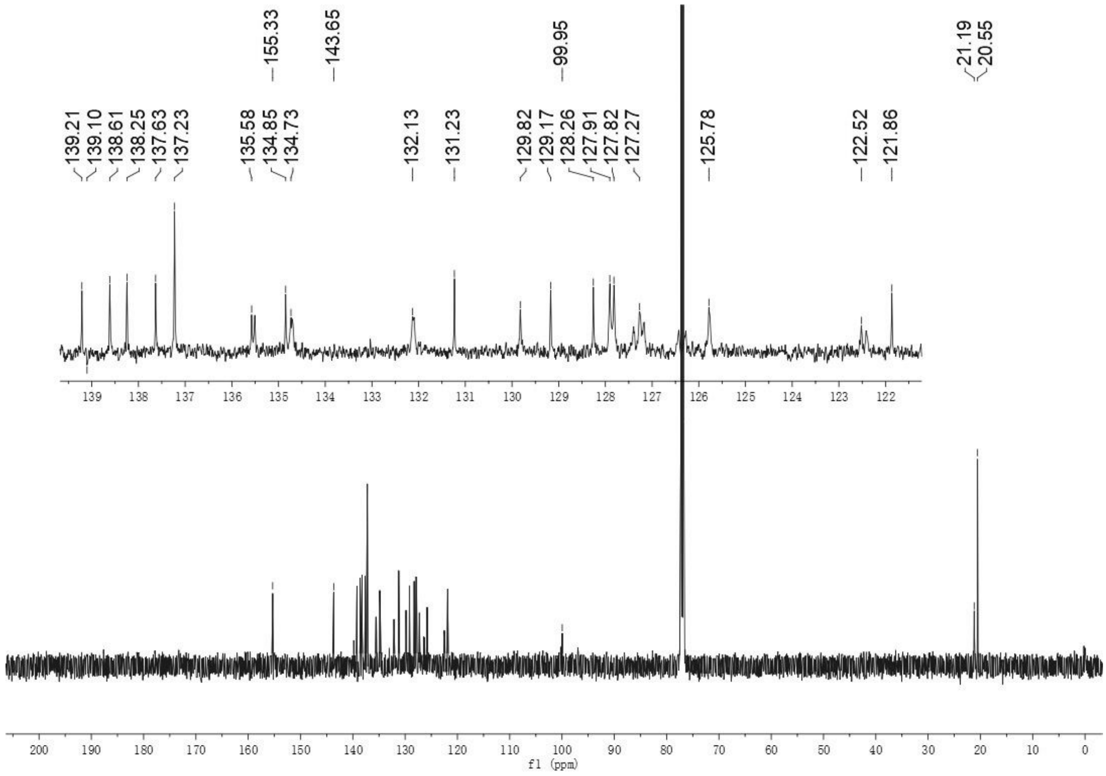

Figure S8. ^{13}C NMR spectrum of **5Pd** in $CDCl_3$.

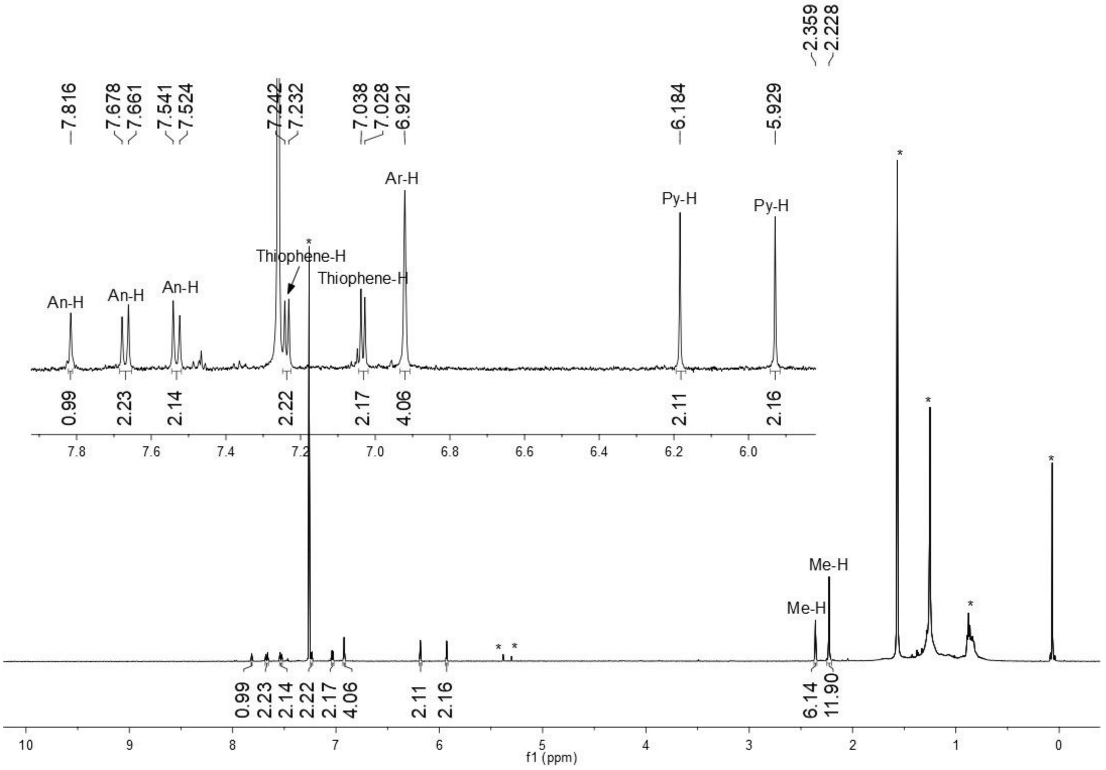

Figure S9. ^{1}H NMR spectrum of **6Pd** in $CDCl_3$. * Solvent or impurities

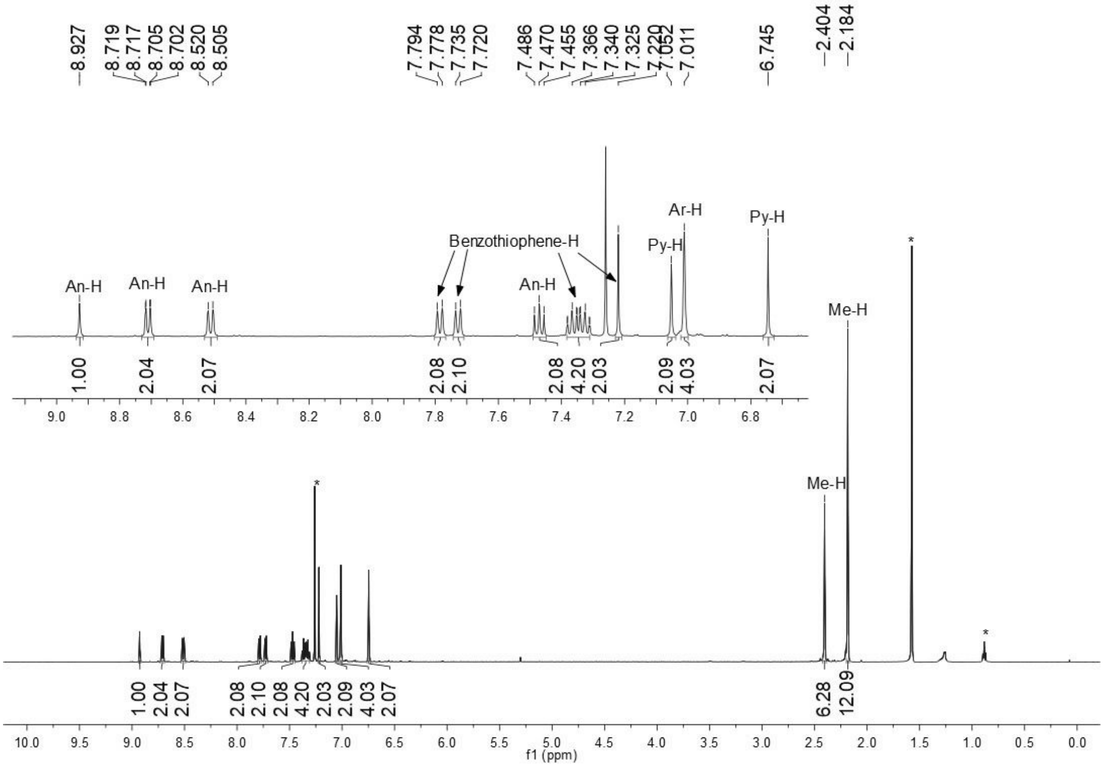

Figure S10. ^{1}H NMR spectrum of **7Pd** in $CDCl_3$. * Solvent or impurities

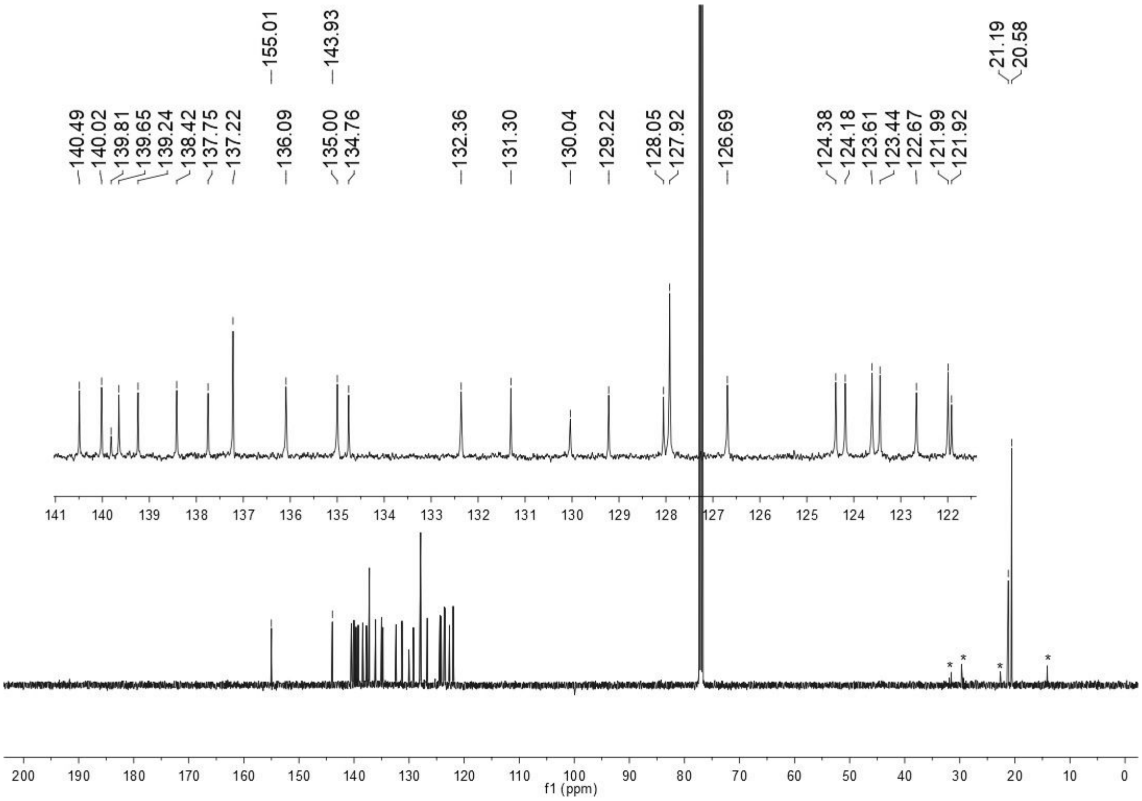

Figure S11. ¹C NMR spectrum of **7Pd** in $CDCl_3$. * Solvent or impurities

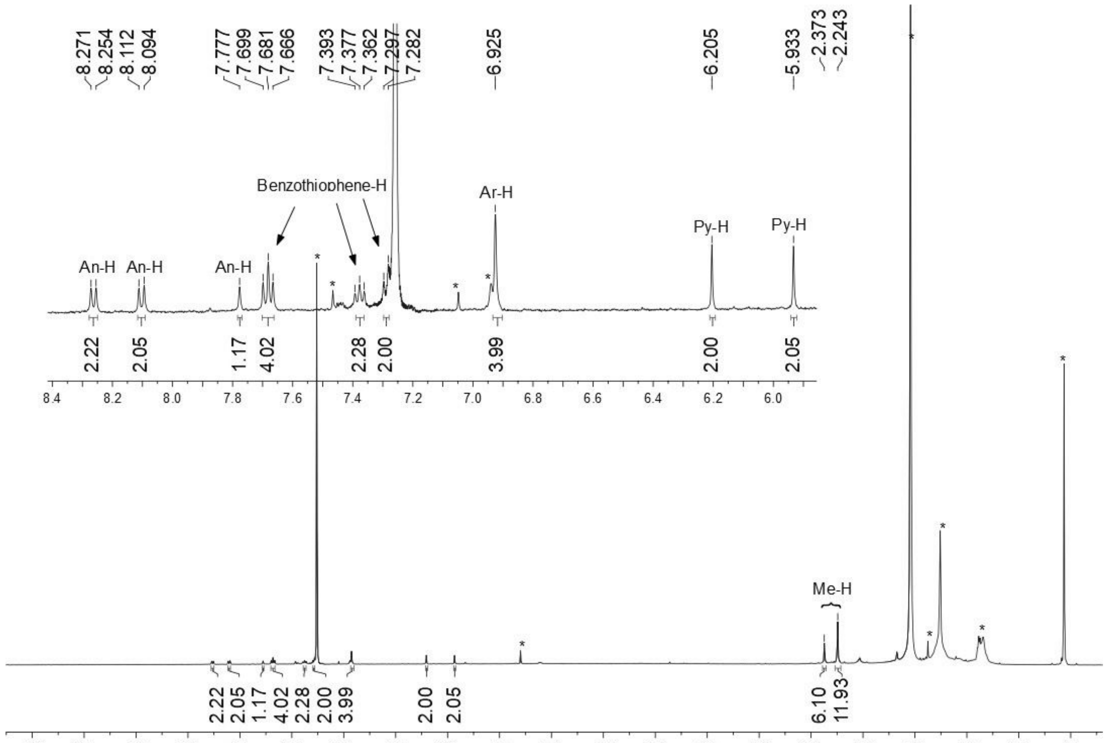

Figure S12. ¹H NMR spectrum of **8Pd** in $CDCl_3$. * Solvent or impurities

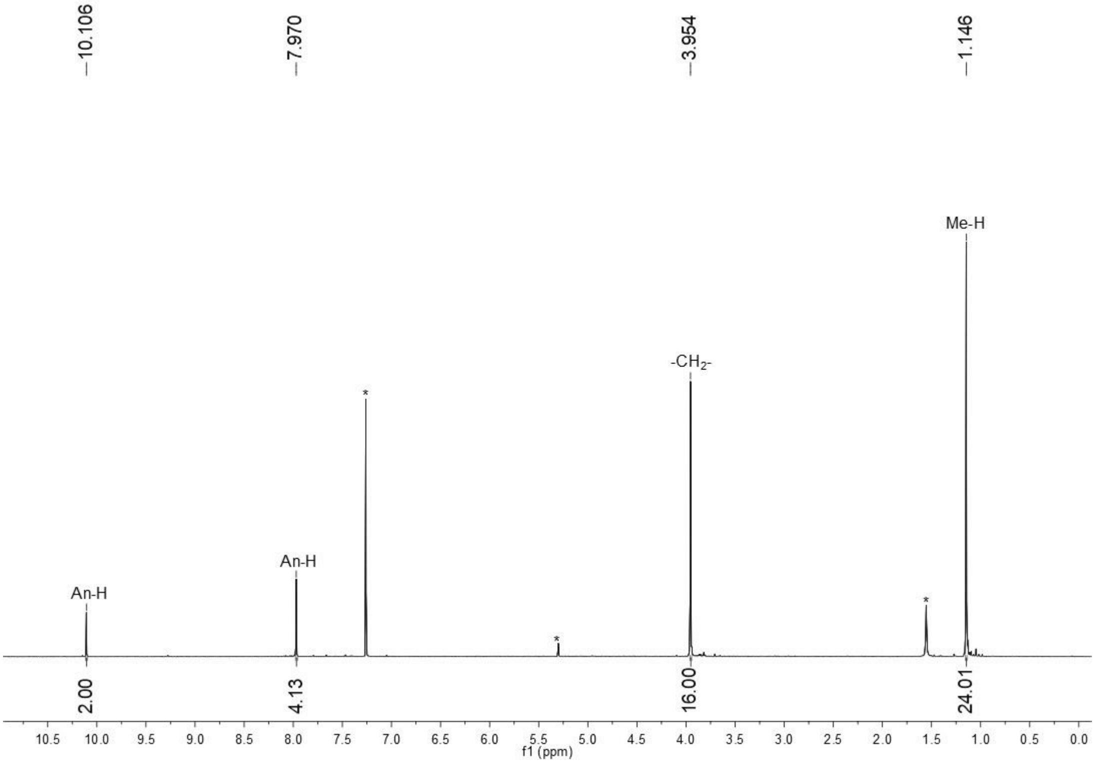

Figure S13. ^{1}H NMR spectrum of **1,4,5,8-tetraboryl anthracene** in $CDCl_3$. * Solvent or impurities

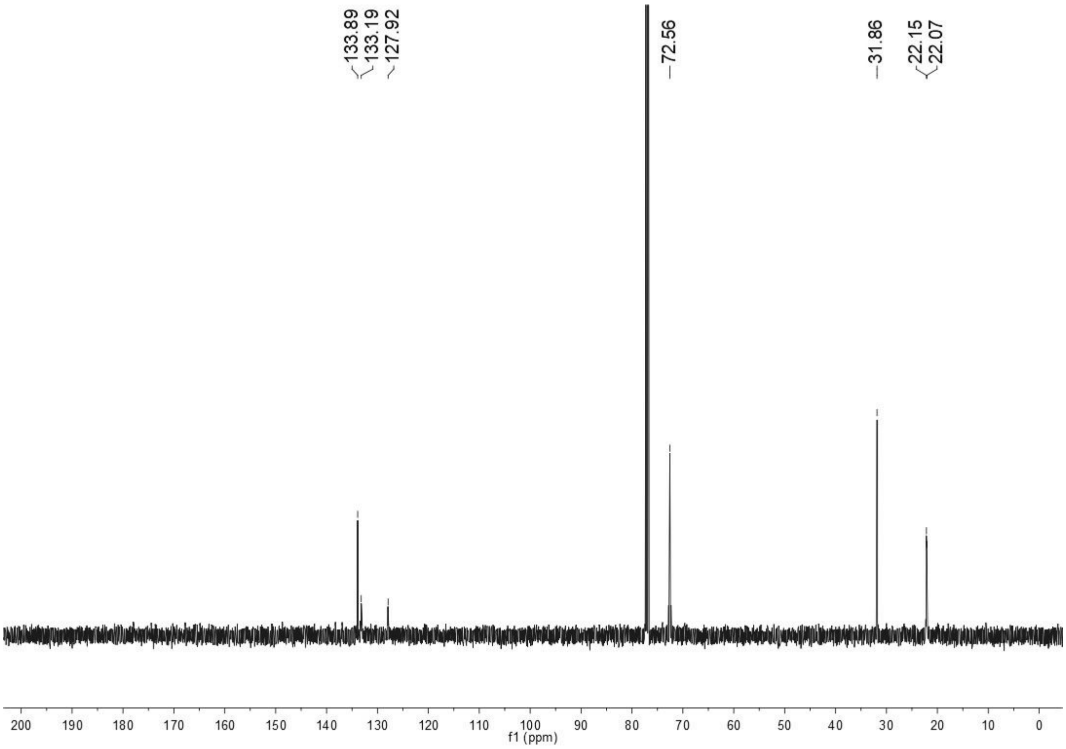

Figure S14. ^{1}C NMR spectrum of **1,4,5,8-tetraboryl anthracene** in $CDCl_3$.

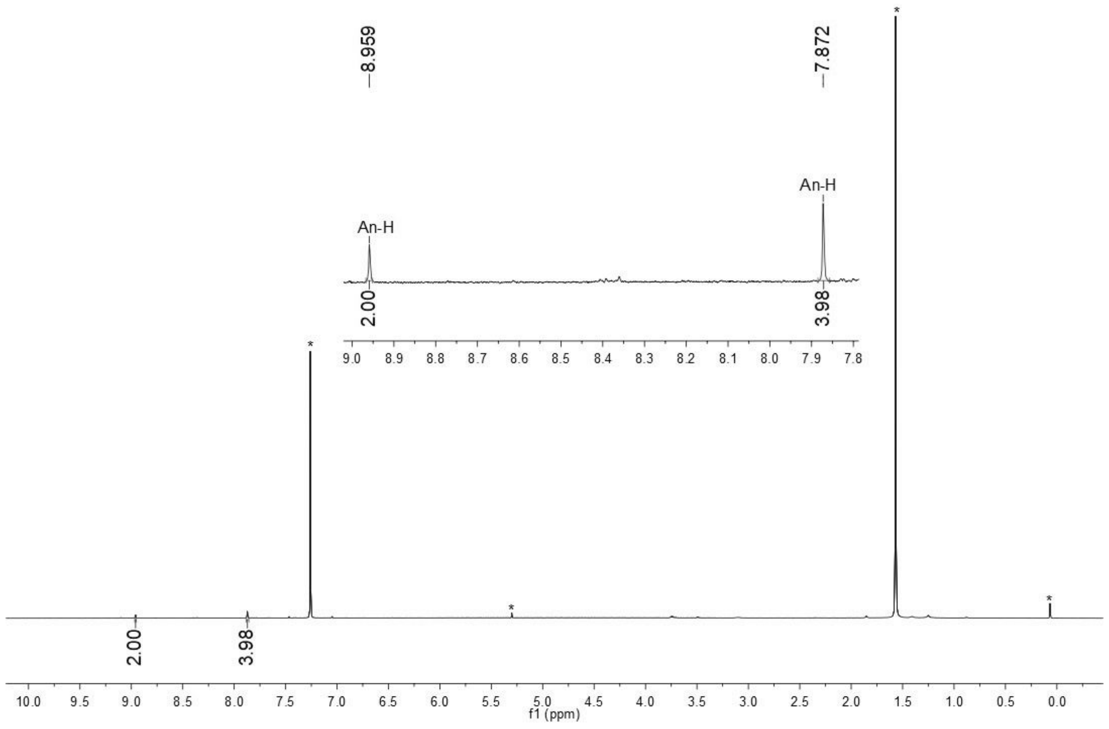

Figure S15. ^{1}H NMR spectrum of **9** in $CDCl_3$. * Solvent or impurities

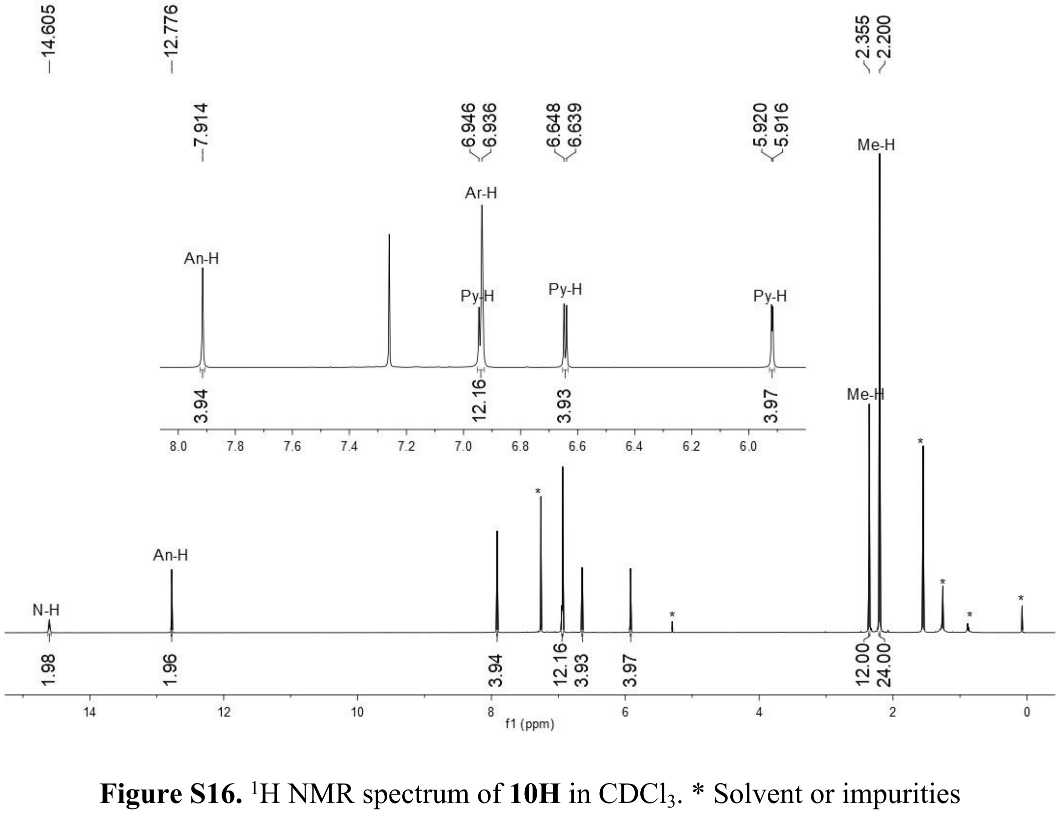

Figure S16. ^{1}H NMR spectrum of **10H** in $CDCl_3$. * Solvent or impurities

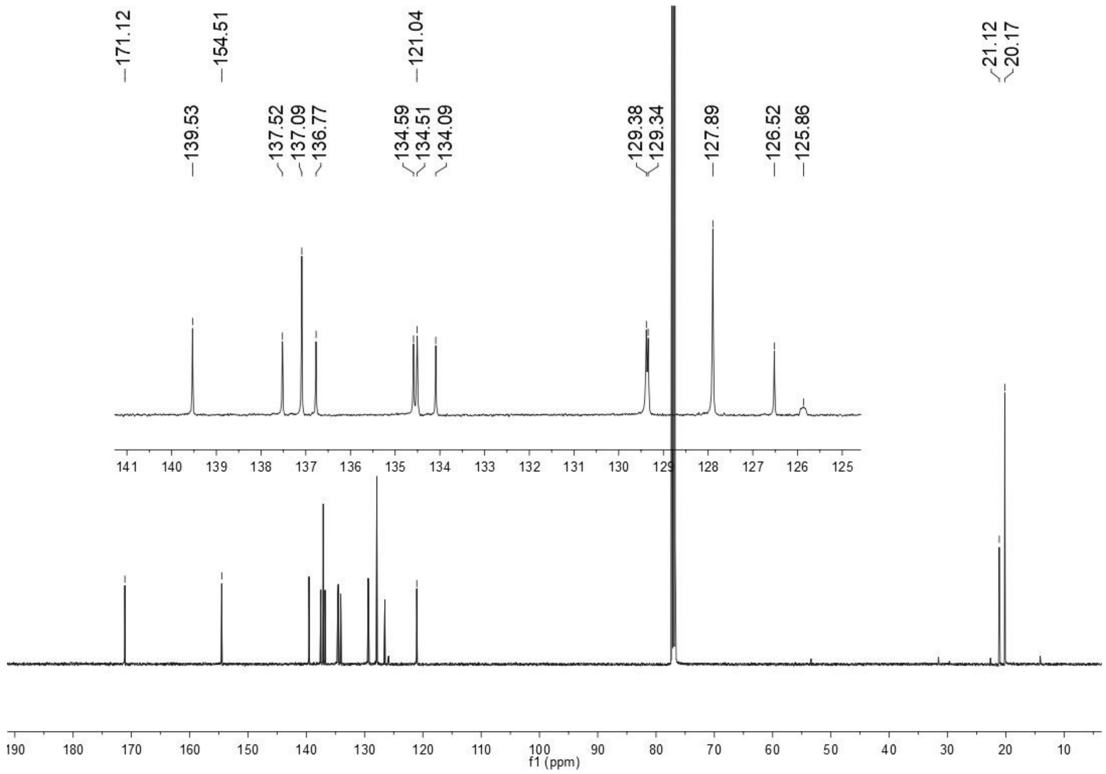

Figure S17. ^{13}C NMR spectrum of **10H** in $CDCl_3$.

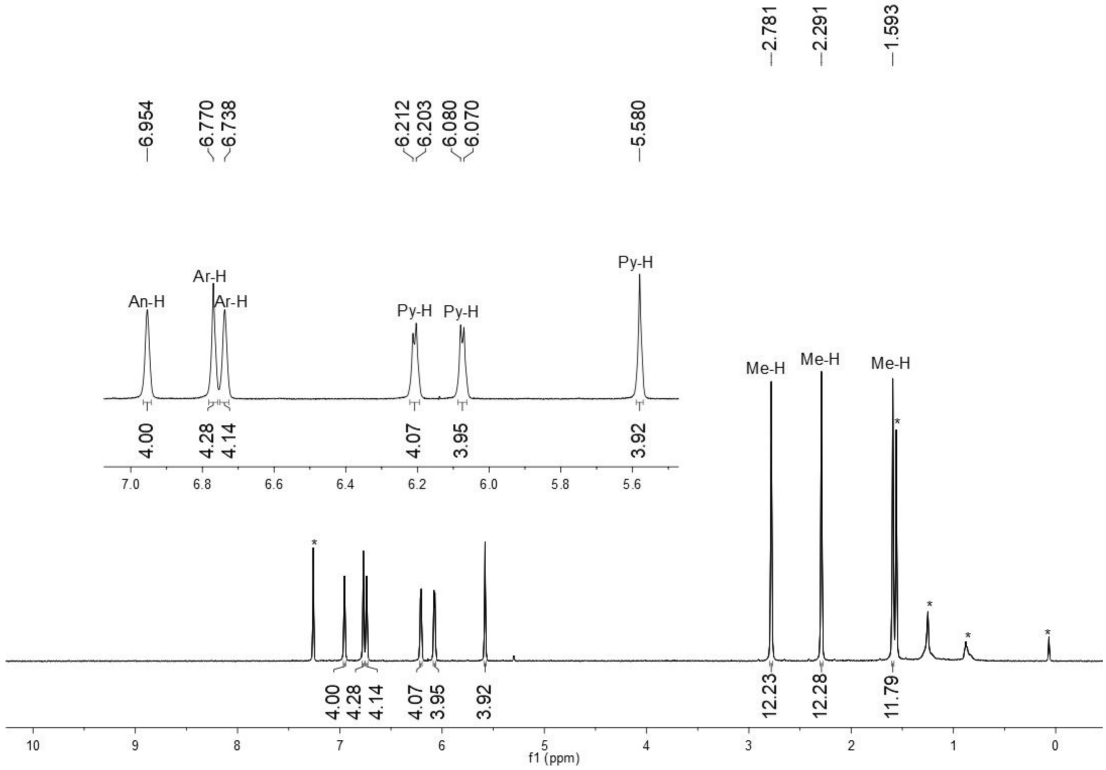

Figure S18. ^{1}H NMR spectrum of **10Pd** in $CDCl_3$. * Solvent or impurities

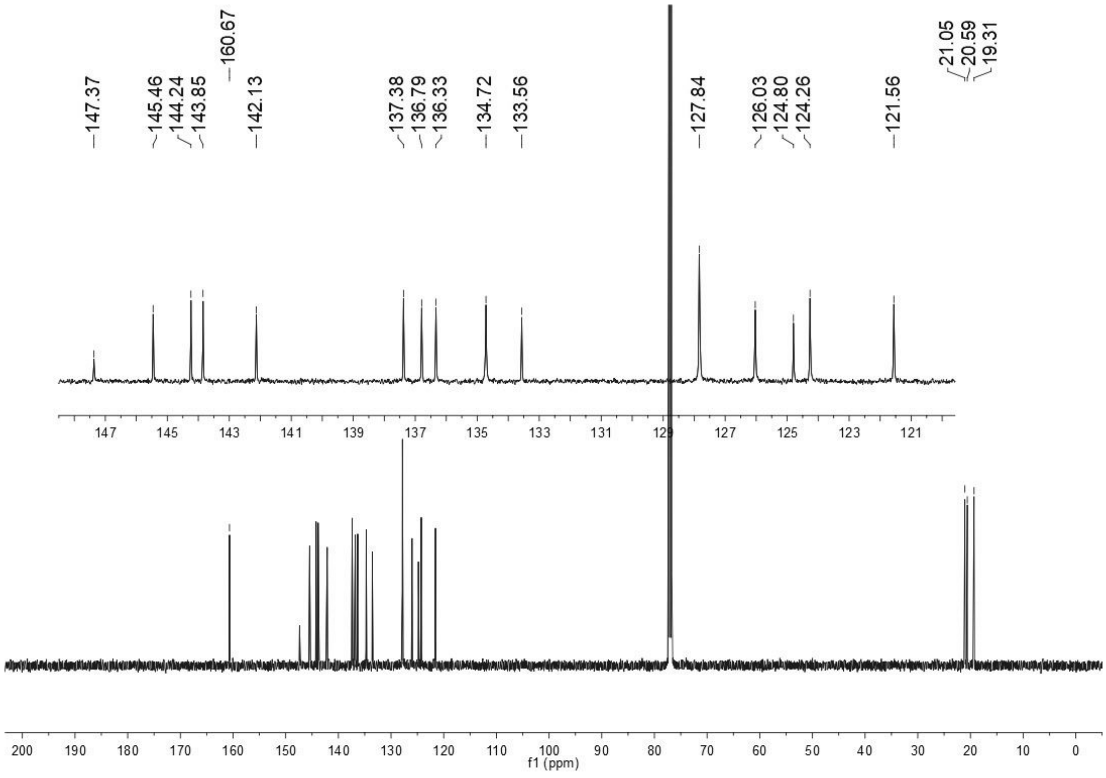

Figure S19. ^{13}C NMR spectrum of **10Pd** in $CDCl_3$.

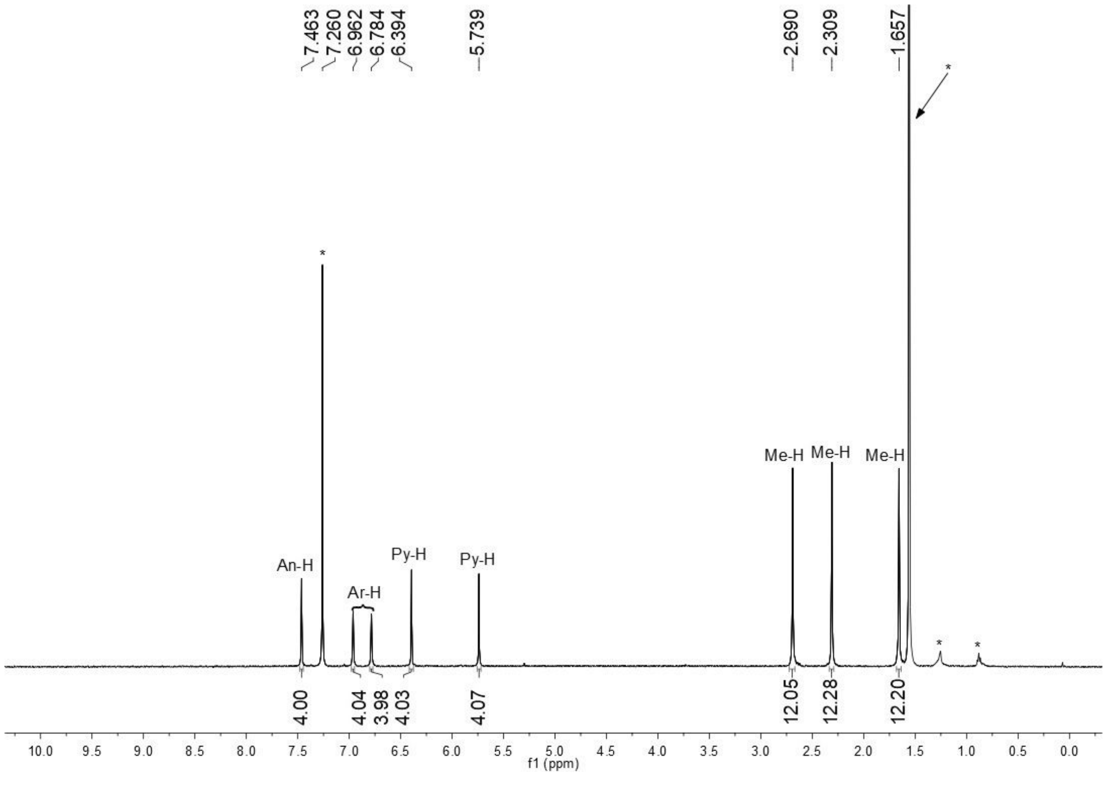

Figure S20. ^{1}H NMR spectrum of **11Pd** in $CDCl_3$. * Solvent or impurities

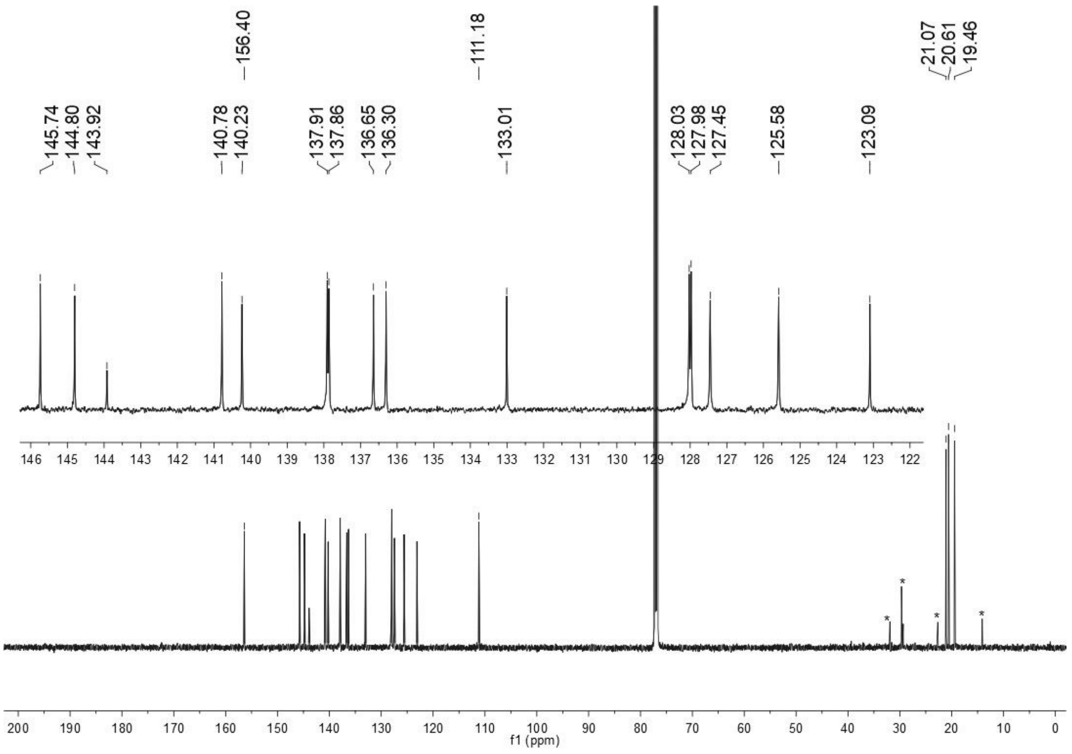

Figure S21. ^{13}C NMR spectrum of **11Pd** in $CDCl_3$. * Solvent or impurities

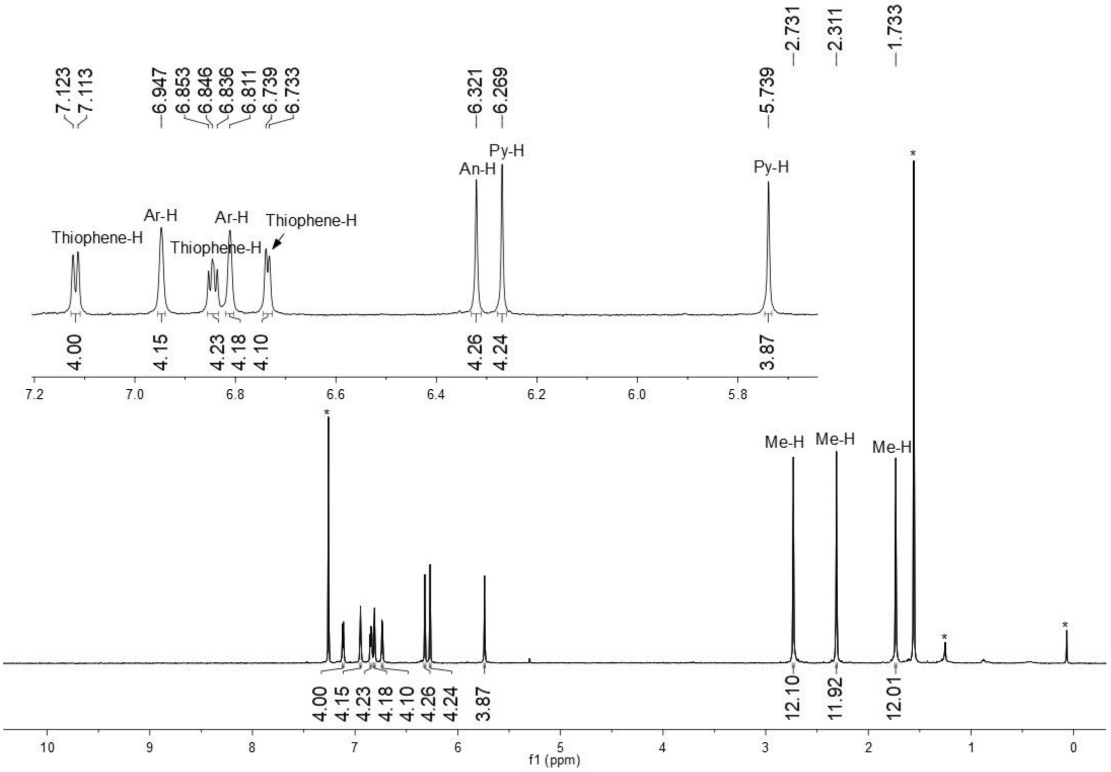

Figure S22. ^{1}H NMR spectrum of **12Pd** in $CDCl_3$. * Solvent or impurities

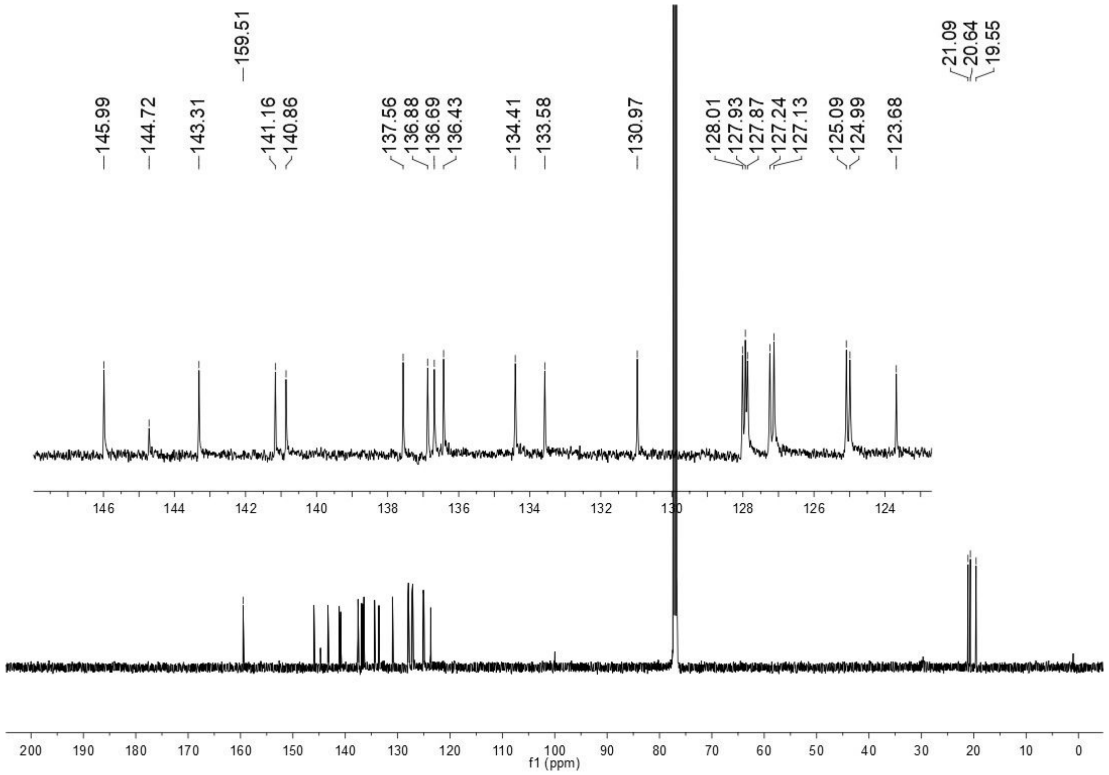

Figure S23. ^{13}C NMR spectrum of **12Pd** in $CDCl_3$.

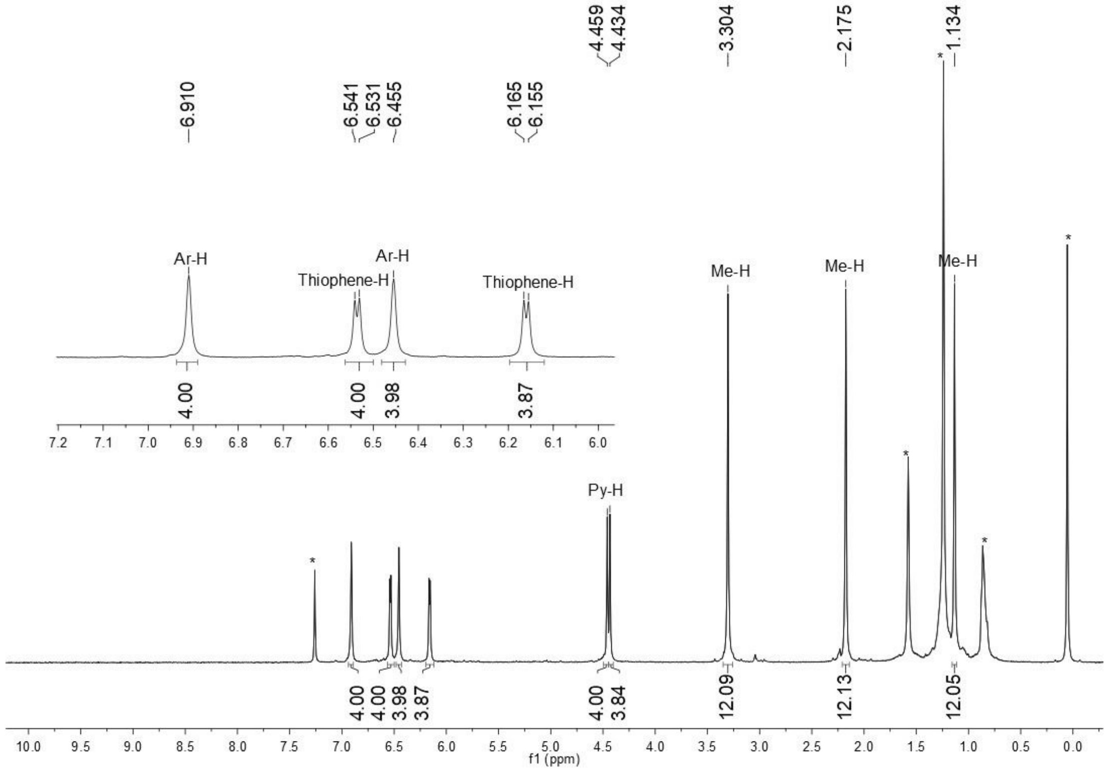

Figure S24. ^{1}H NMR spectrum of **13Pd** in $CDCl_3$. * Solvent or impurities

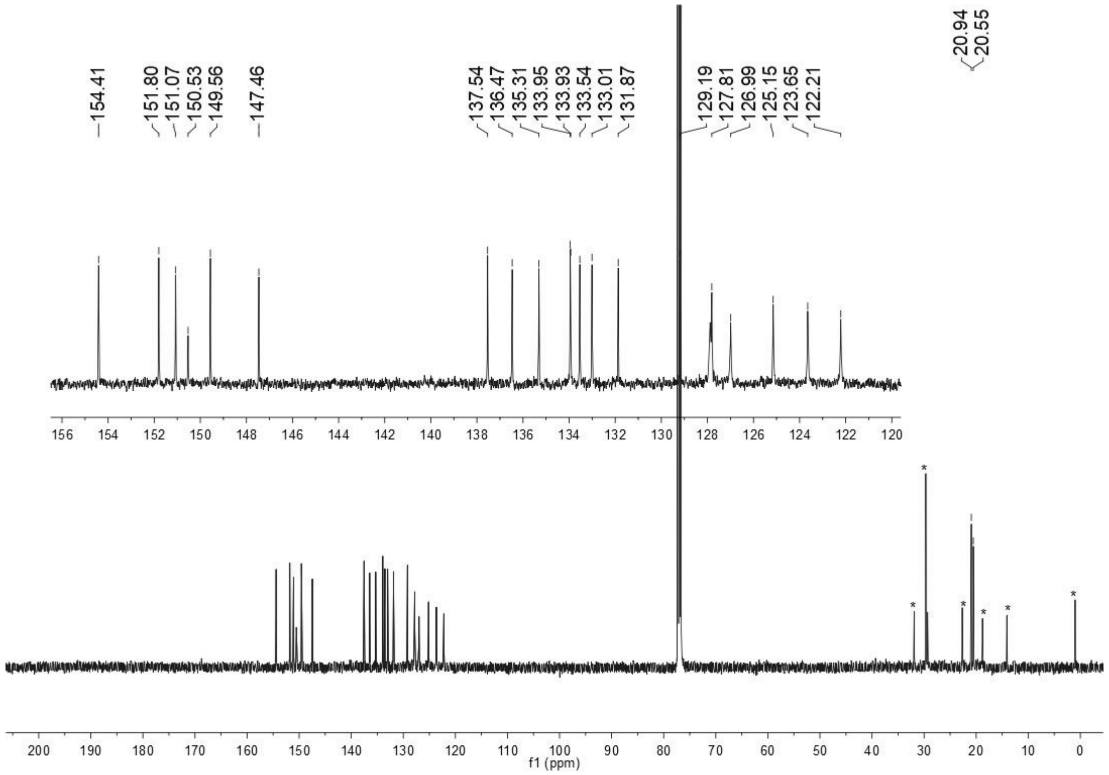

Figure S25. [1]C NMR spectrum of **13Pd** in $CDCl_3$. * Solvent or impurities

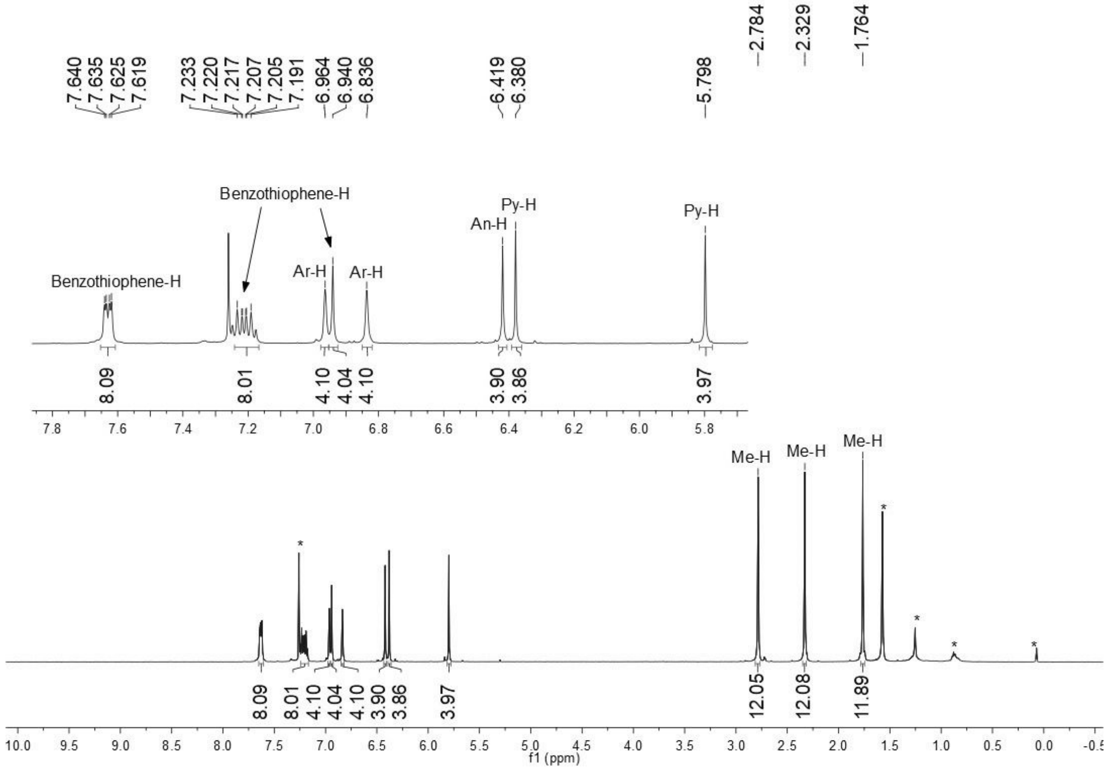

Figure S26. ^{1}H NMR spectrum of **14Pd** in $CDCl_3$. * Solvent or impurities

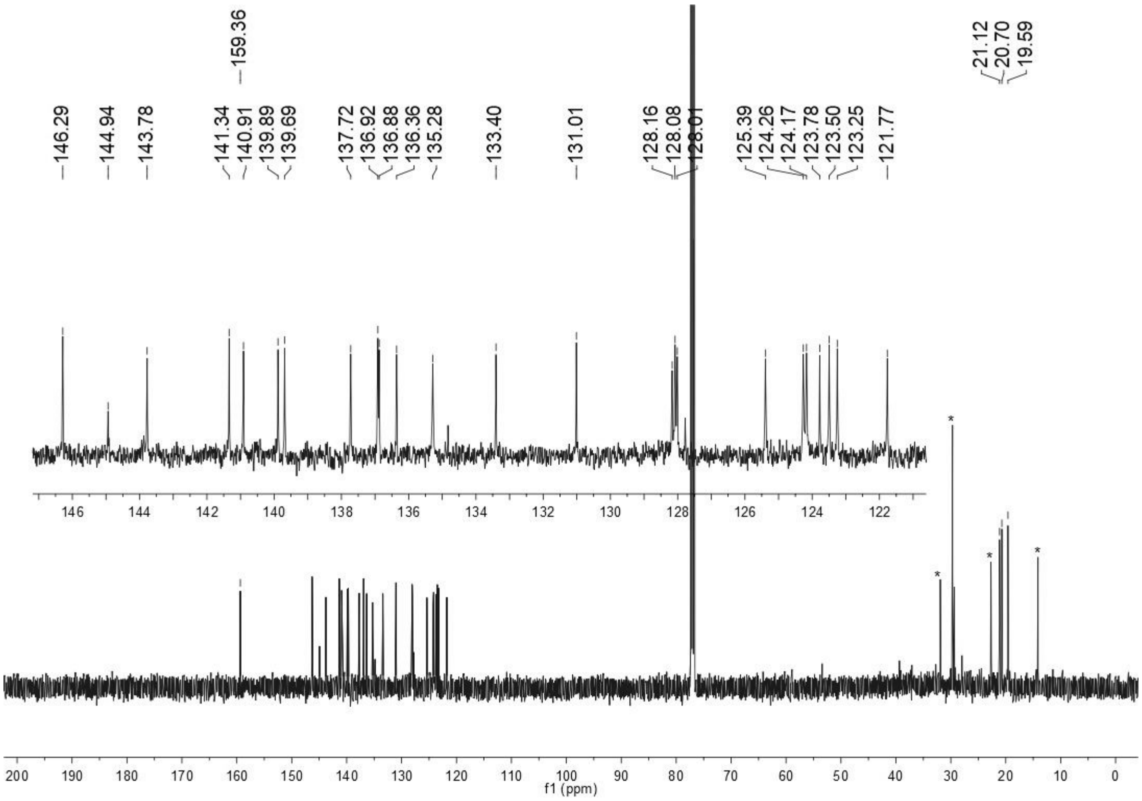

Figure S27. [1]C NMR spectrum of **14Pd** in $CDCl_3$. * Solvent or impurities

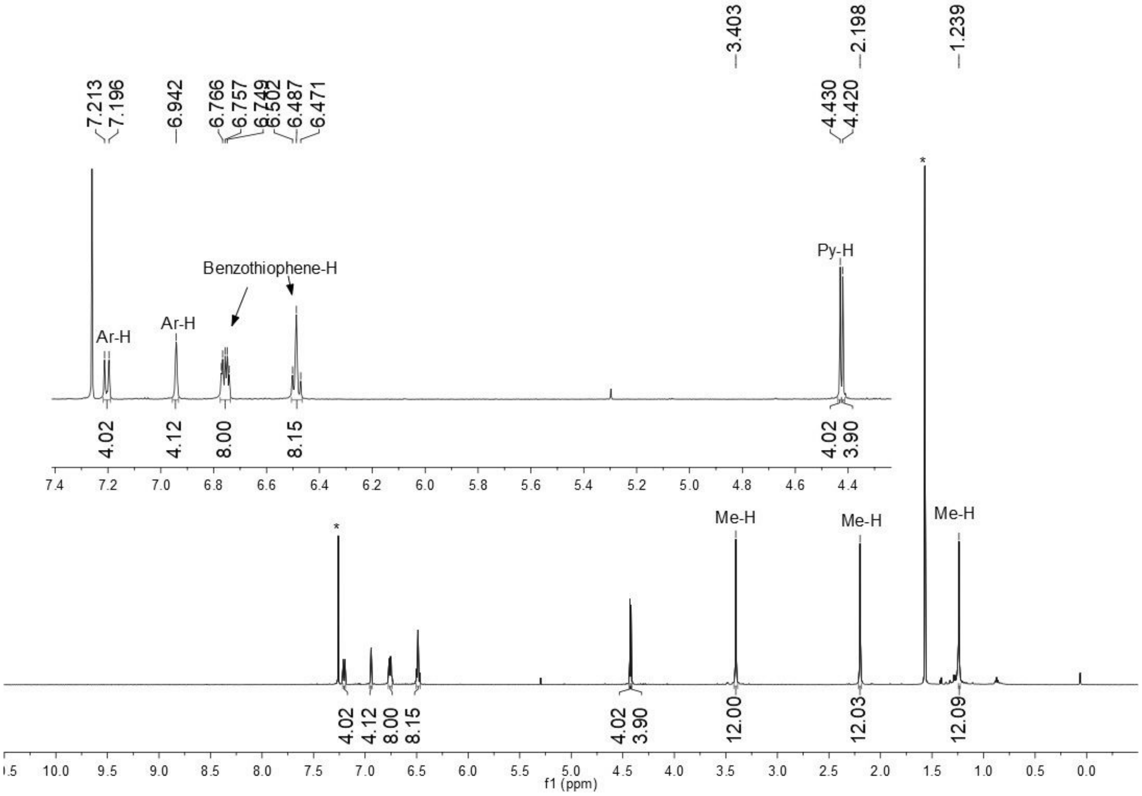

Figure S28. ^{1}H NMR spectrum of **15Pd** in $CDCl_3$. * Solvent or impurities

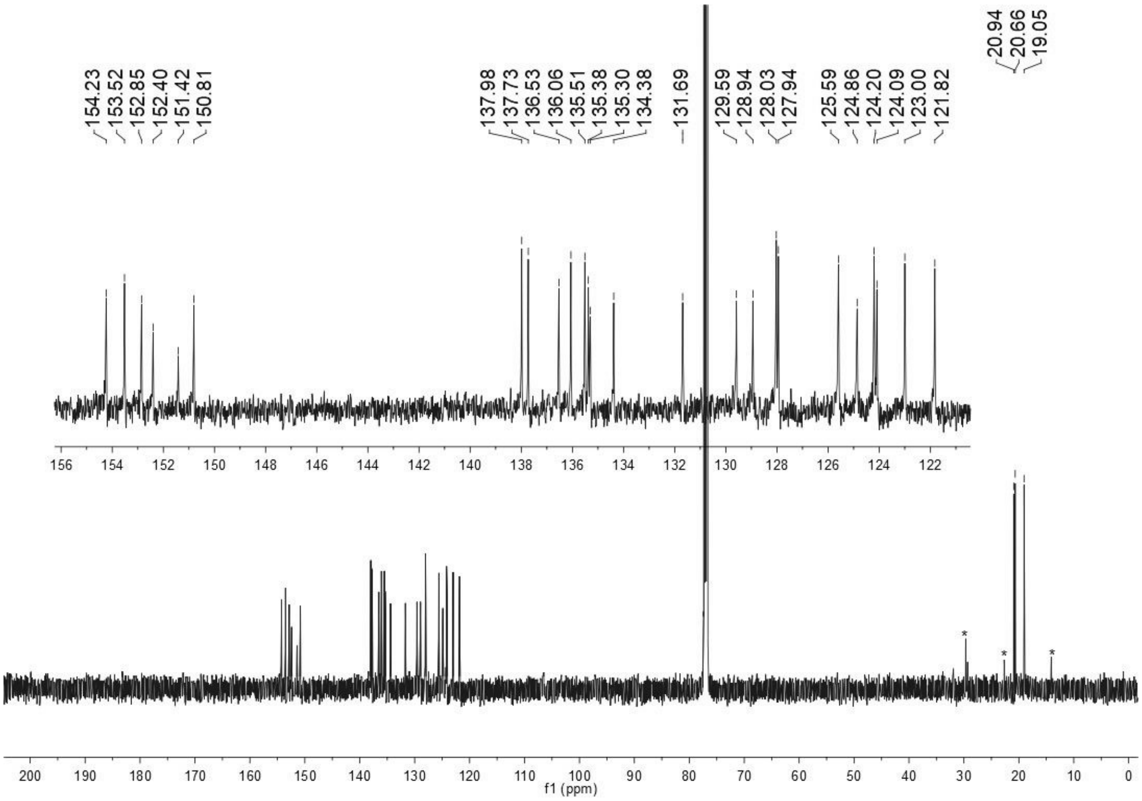

Figure S29. ^{1}C NMR spectrum of **15Pd** in $CDCl_3$. * Solvent or impurities

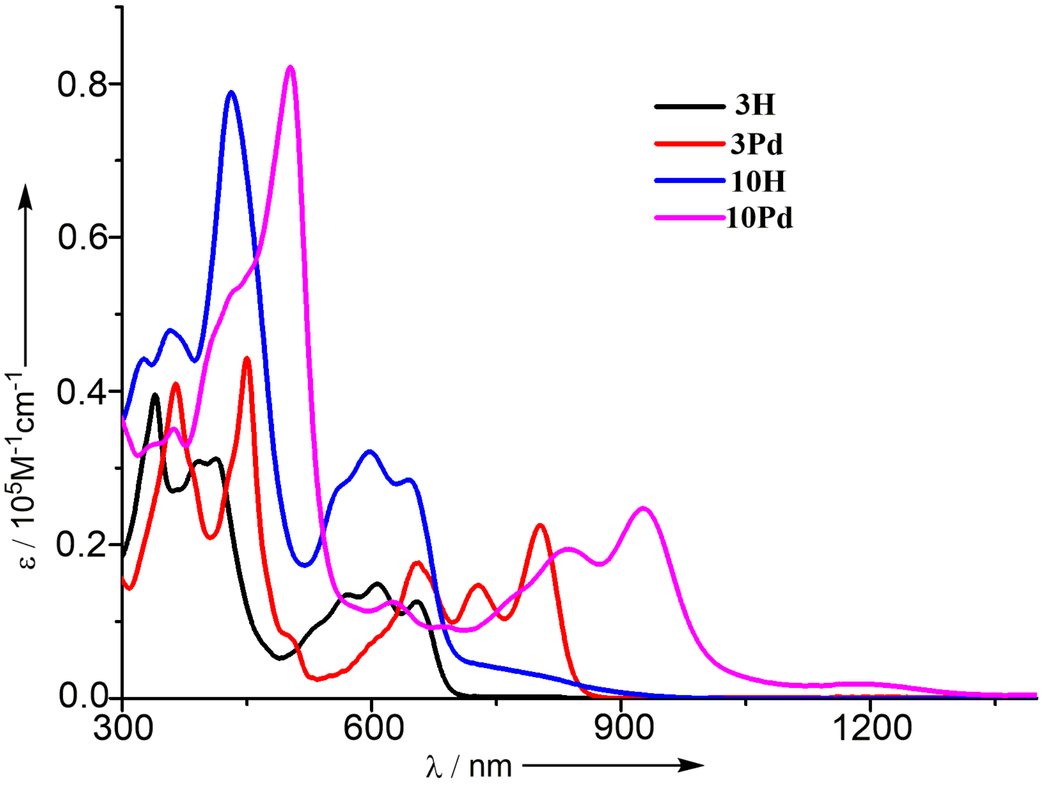

Figure S30. UV/vis absorption spectra of **3H**, **3Pd**, **10H** and **10Pd** in CH_2Cl_2.

Table S1. UV/vis absorption spectra data of **3H**, **3Pd**, **10H** and **10Pd**.

Compound	λ/ nm	ε / 10^5 M/cm^{-1}
**3H**	339, 392, 413, 570, 607, 654	0.40, 0.31, 0.31, 0.13, 0.15, 0.13
**3Pd**	364, 450, 656, 728, 803	0.41, 0.44, 0.18, 0.15, 0.23
**10H**	326, 358, 431, 598, 644	0.44, 0.48, 0.79, 0.32, 0.28
**10Pd**	362, 502, 625, 837, 926	0.35, 0.82, 0.13, 0.19, 0.25

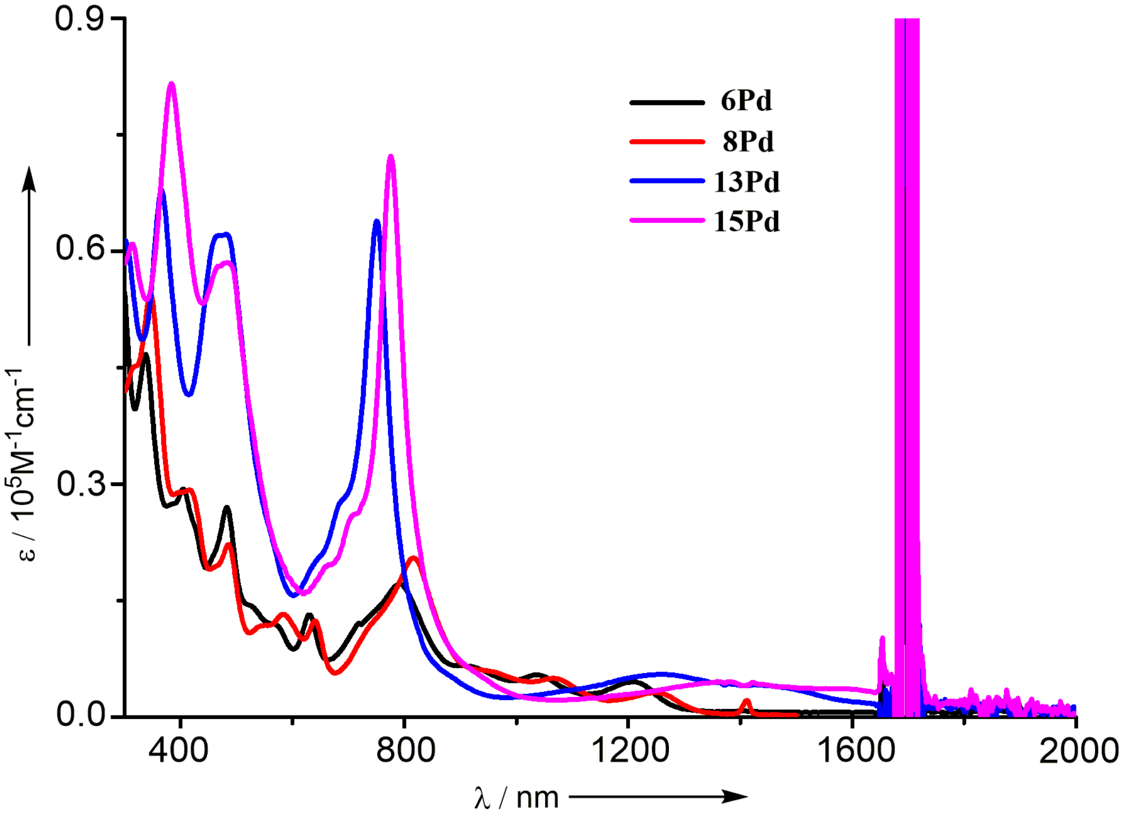

Figure S31. UV/vis absorption spectra of **6Pd**, **8Pd**, **13Pd**, and **15Pd** in CH_2Cl_2.

Table S2. UV/vis absorption spectra data of **6Pd**, **8Pd**, **13Pd**, and **15Pd**.

Compound	λ/ nm	ε / 10^5 M/cm^{-1}
**6Pd**	338, 405, 483, 630, 718, 791, 907, 1039, 1209	0.47, 0.29, 0.27, 0.13, 0.12, 0.17, 0.066, 0.054, 0.046
**8Pd**	346, 416, 485, 551, 583, 640, 816, 1067, 1249	0.55, 0.29, 0.22, 0.12, 0.13, 0.12, 0.20, 0.050, 0.031
**13Pd**	365, 481, 750, 1258	0.68, 0.62, 0.64, 0.055
**15Pd**	314, 383, 483, 781, 1378	0.66, 0.85, 0.60, 0.72, 0.051

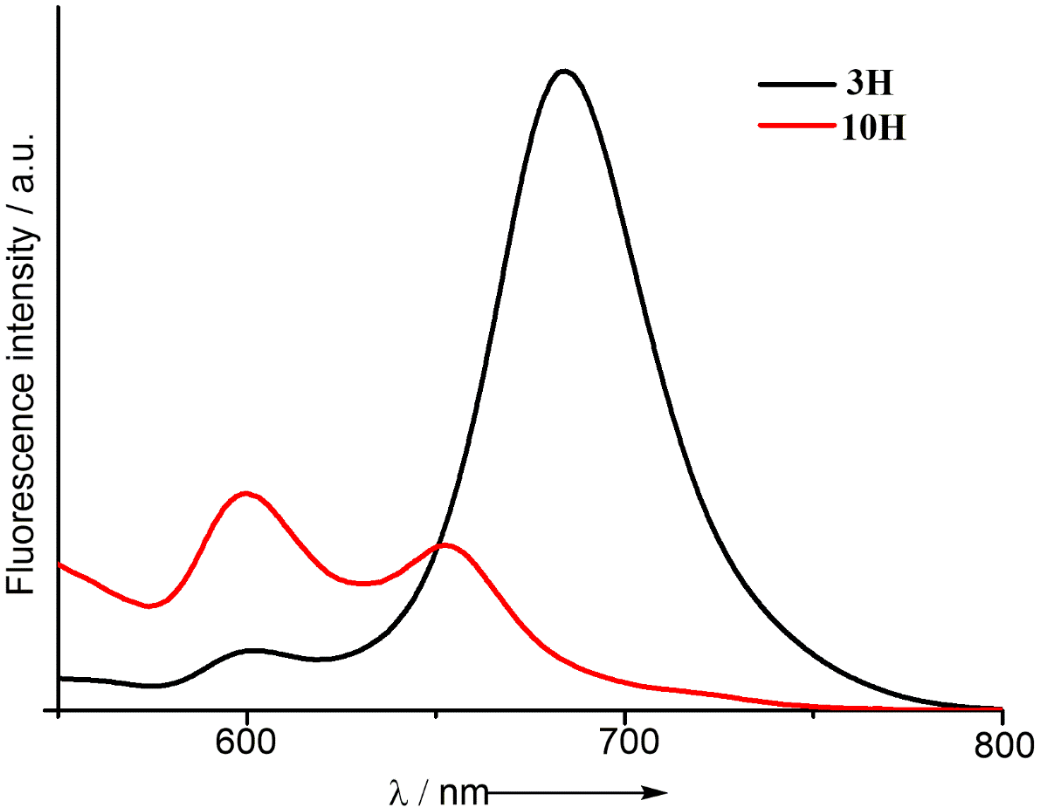

Figure S32. Fluorescence spectra of **3H** (black line), **10H** (red line) in CH_2Cl_2.

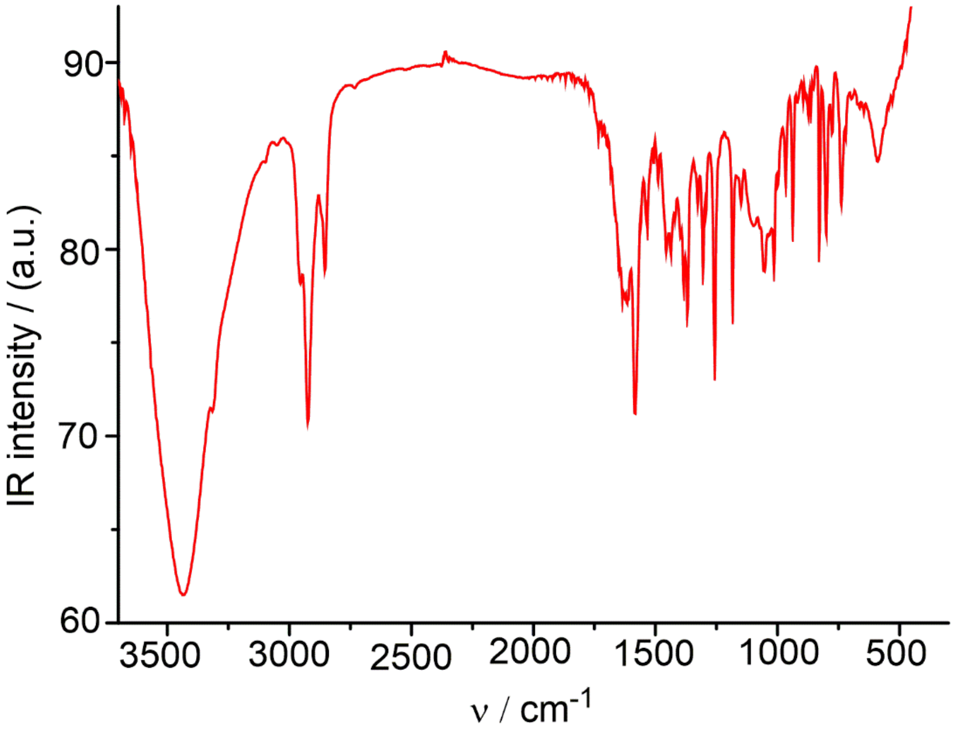

Figure S33. IR spectrum of **3H** (KBr disk).

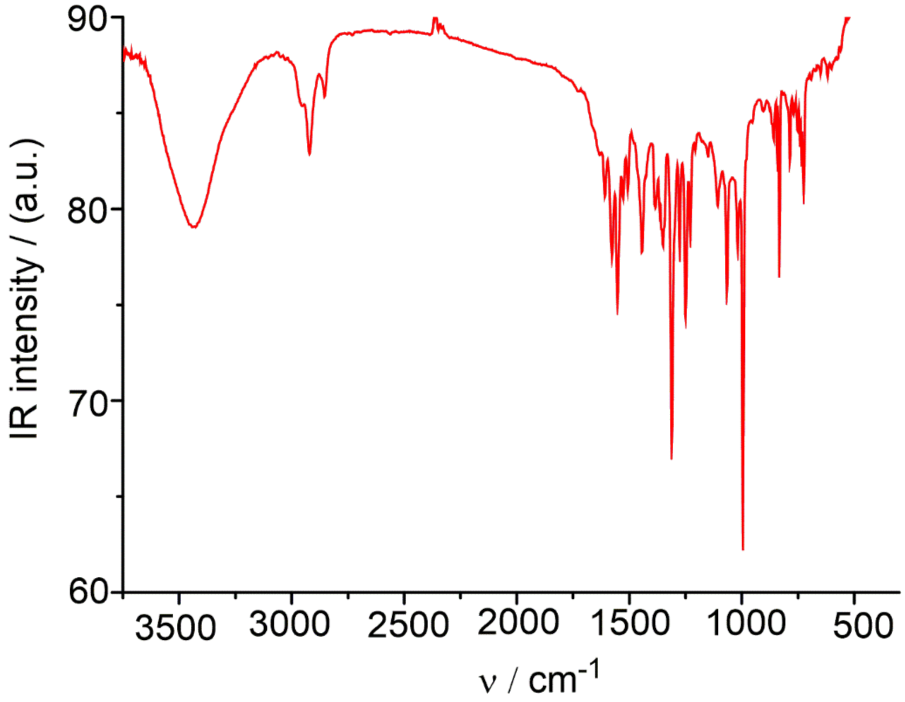

Figure S34. IR spectrum of **3Pd** (KBr disk).

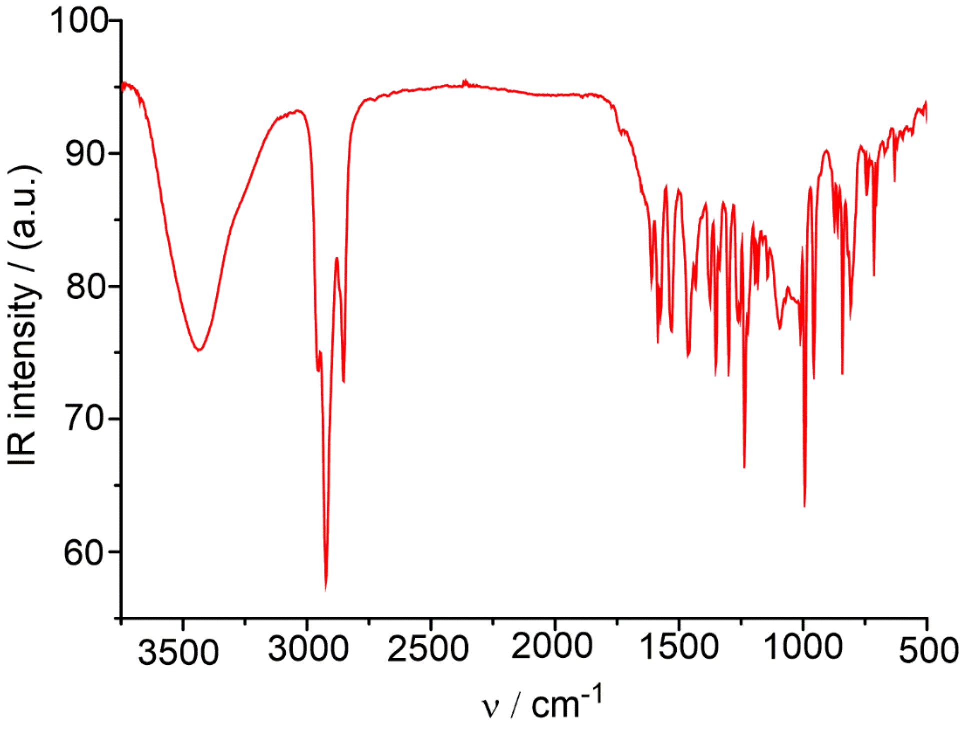

Figure S35. IR spectrum of **6Pd** (KBr disk).

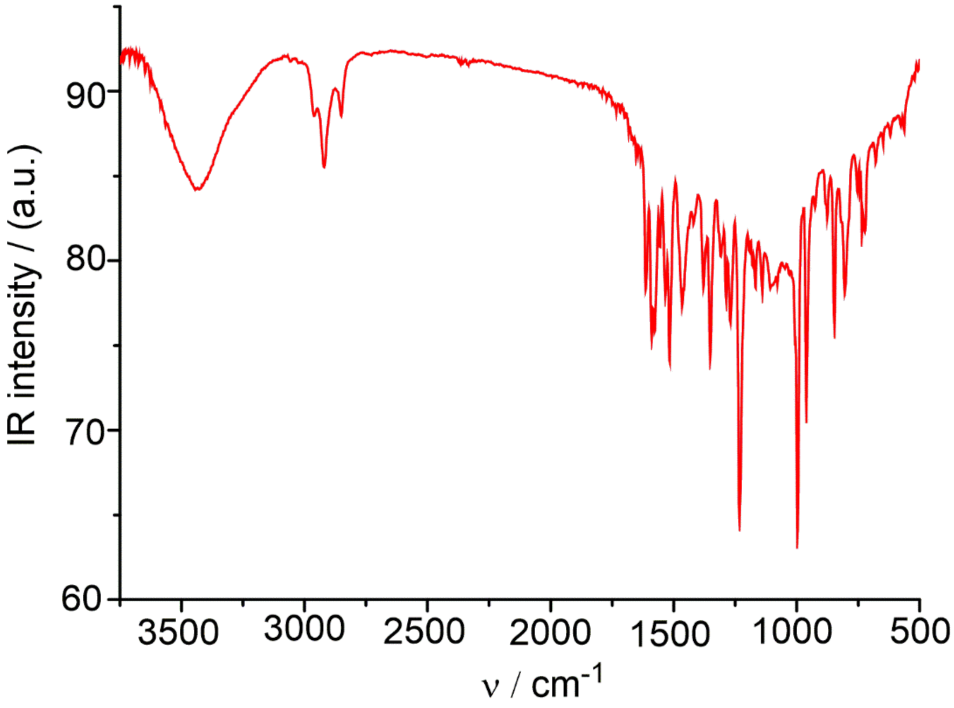

Figure S36. IR spectrum of **8Pd** (KBr disk).

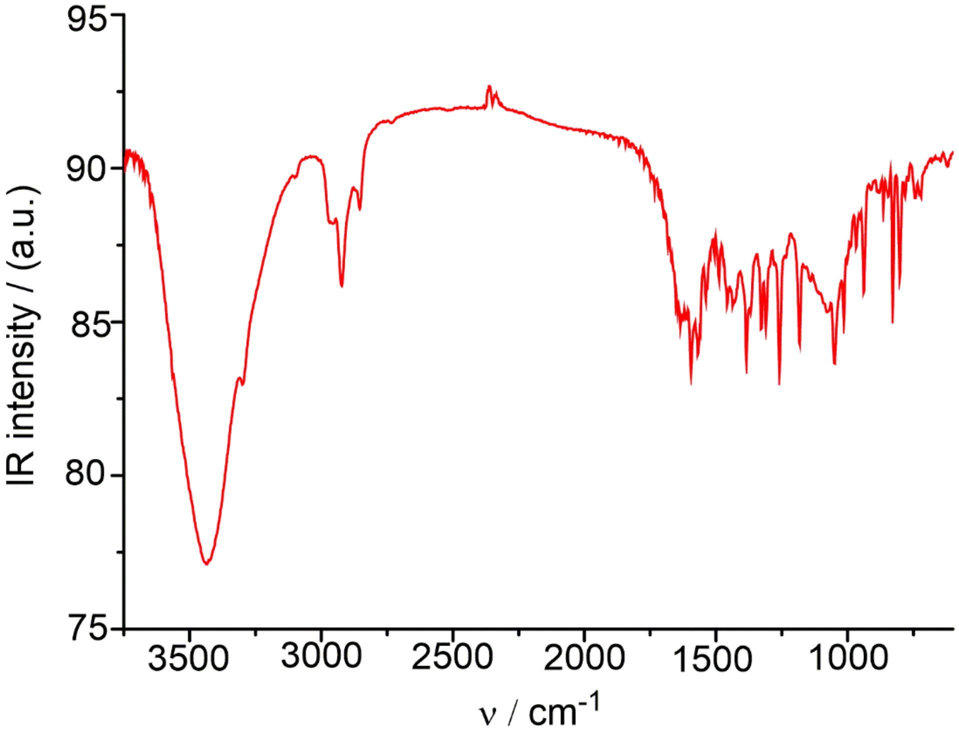

Figure S37. IR spectrum of **10H** (KBr disk).

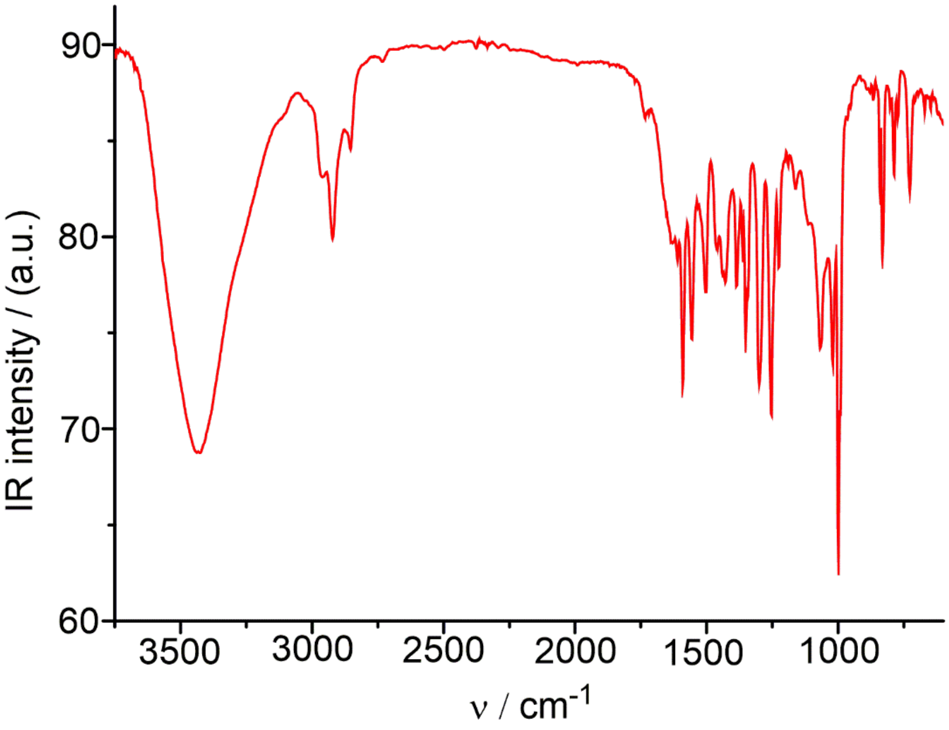

Figure S38. IR spectrum of **10Pd** (KBr disk).

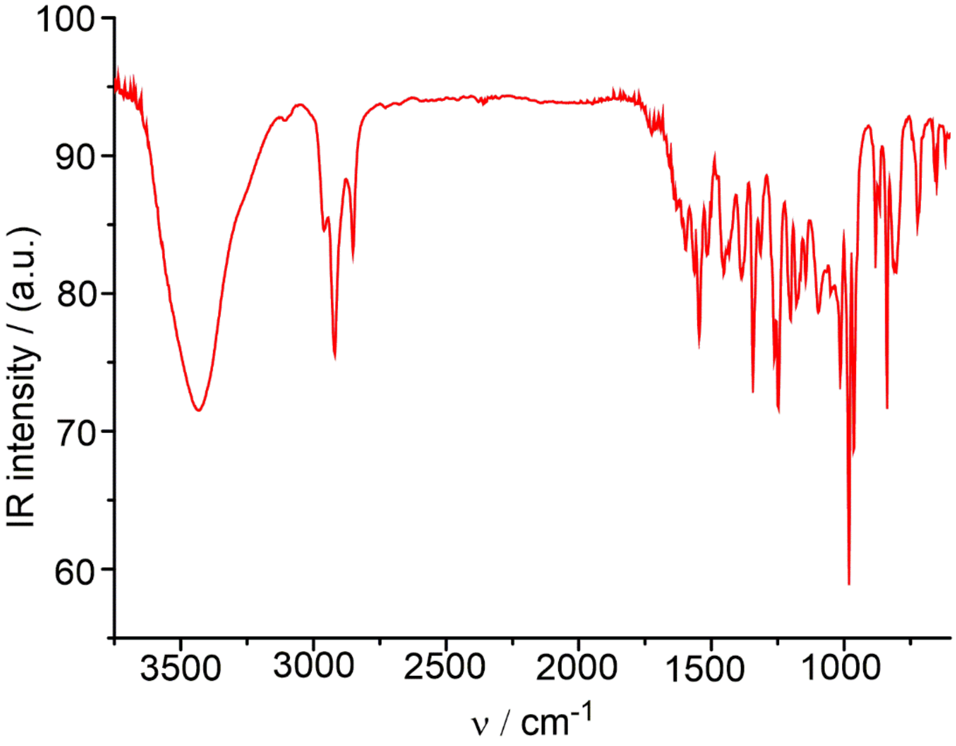

Figure S39. IR spectrum of **13Pd** (KBr disk).

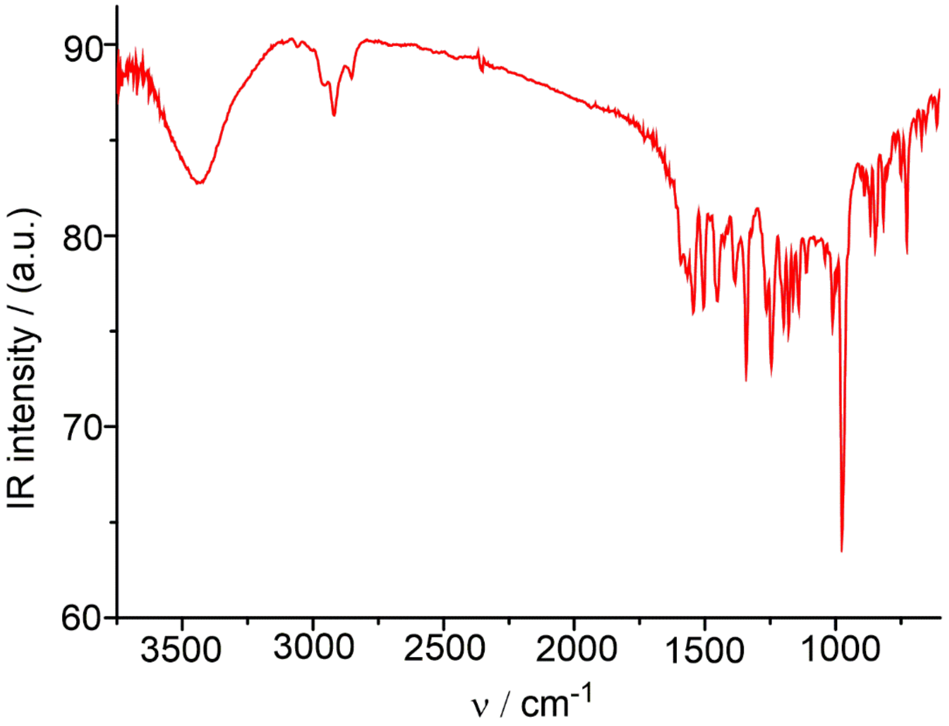

Figure S40. IR spectrum of **15Pd** (KBr disk).

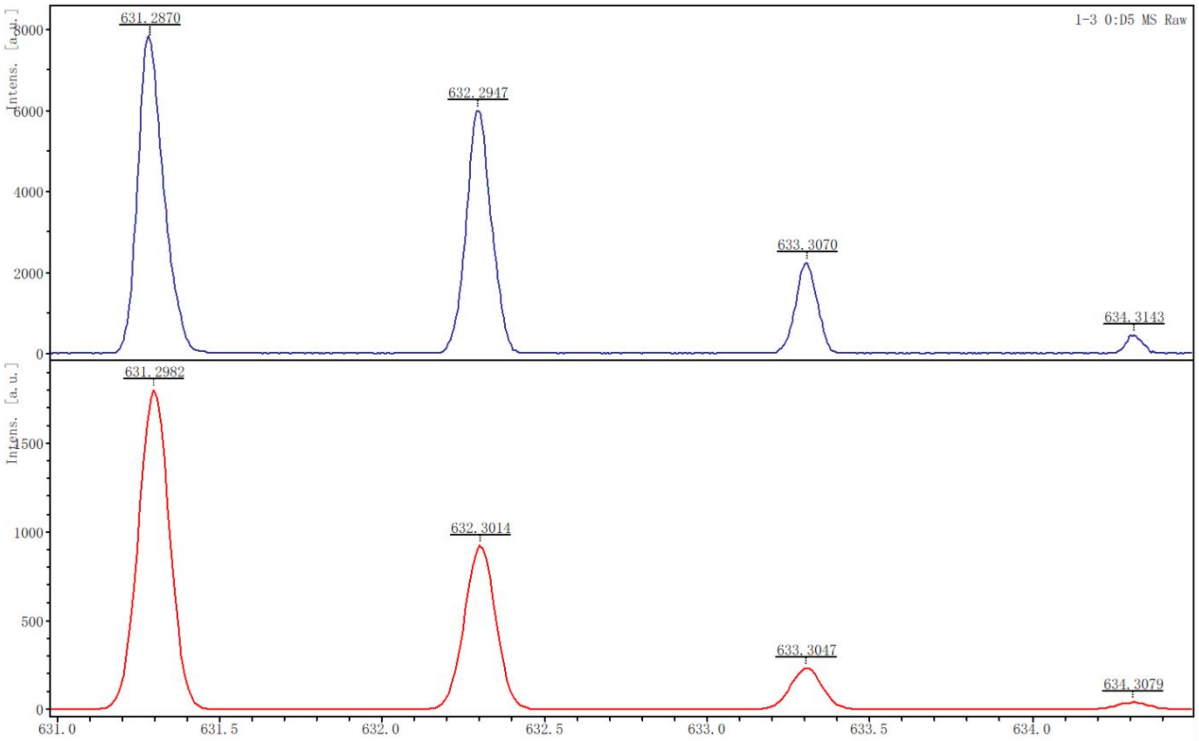

Figure S41. MALDI-TOF-MS spectrum of **3H**.

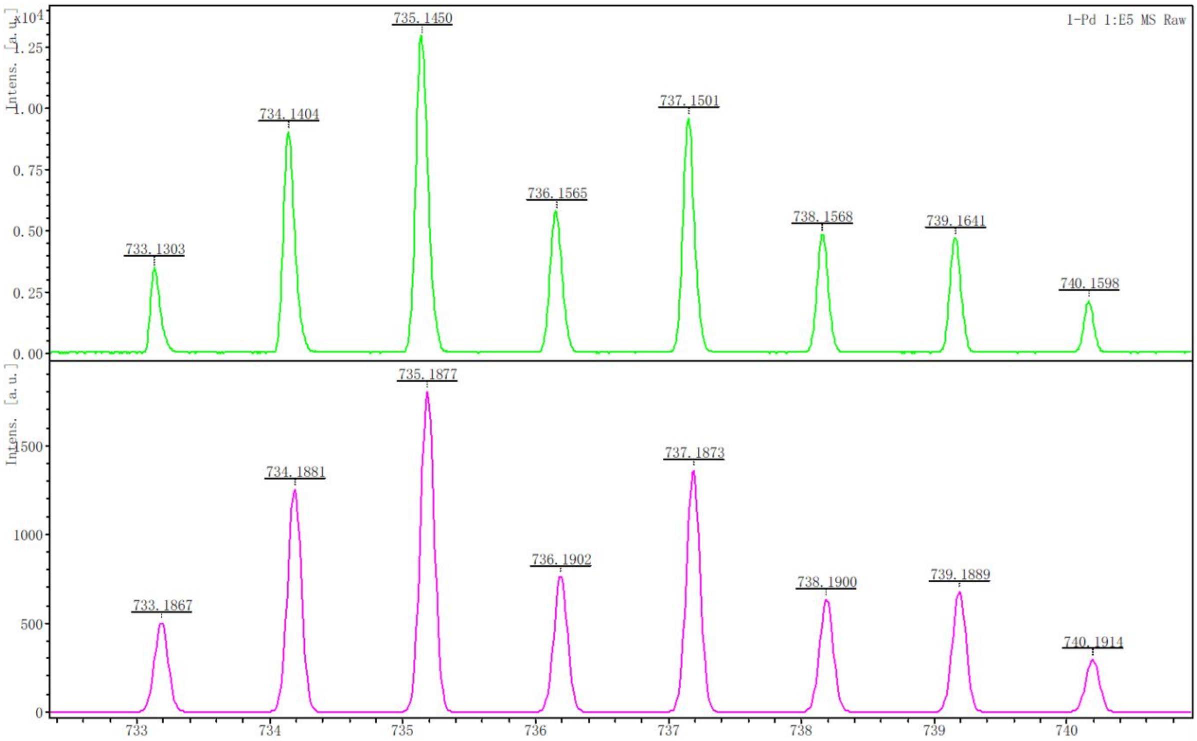

Figure S42. MALDI-TOF-MS spectrum of **3Pd**.

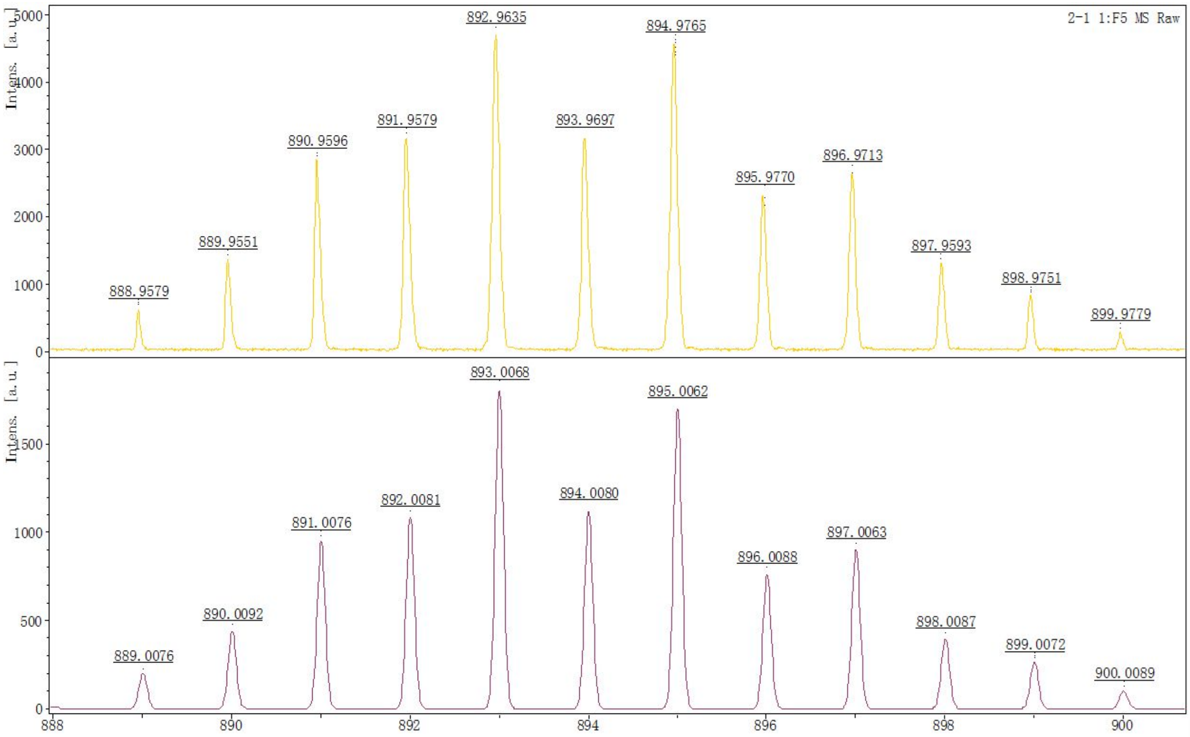

Figure S43. MALDI-TOF-MS spectrum of **4Pd**.

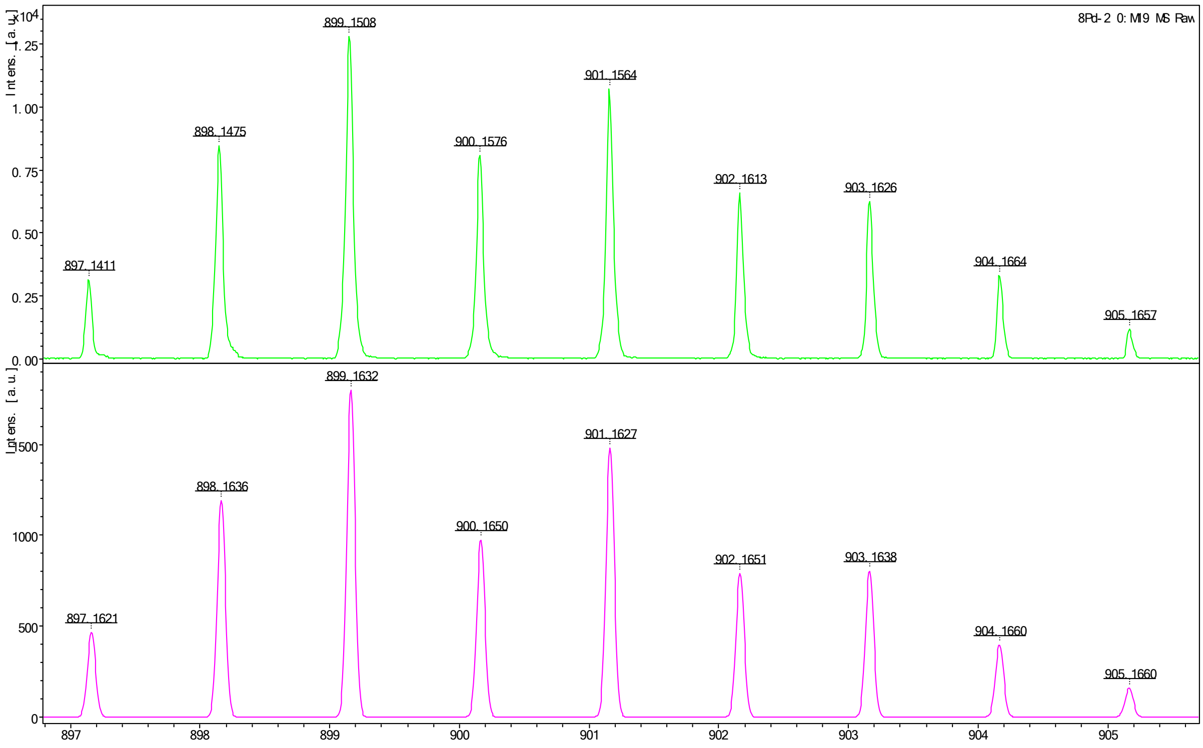

Figure S44. MALDI-TOF-MS spectrum of **5Pd**.

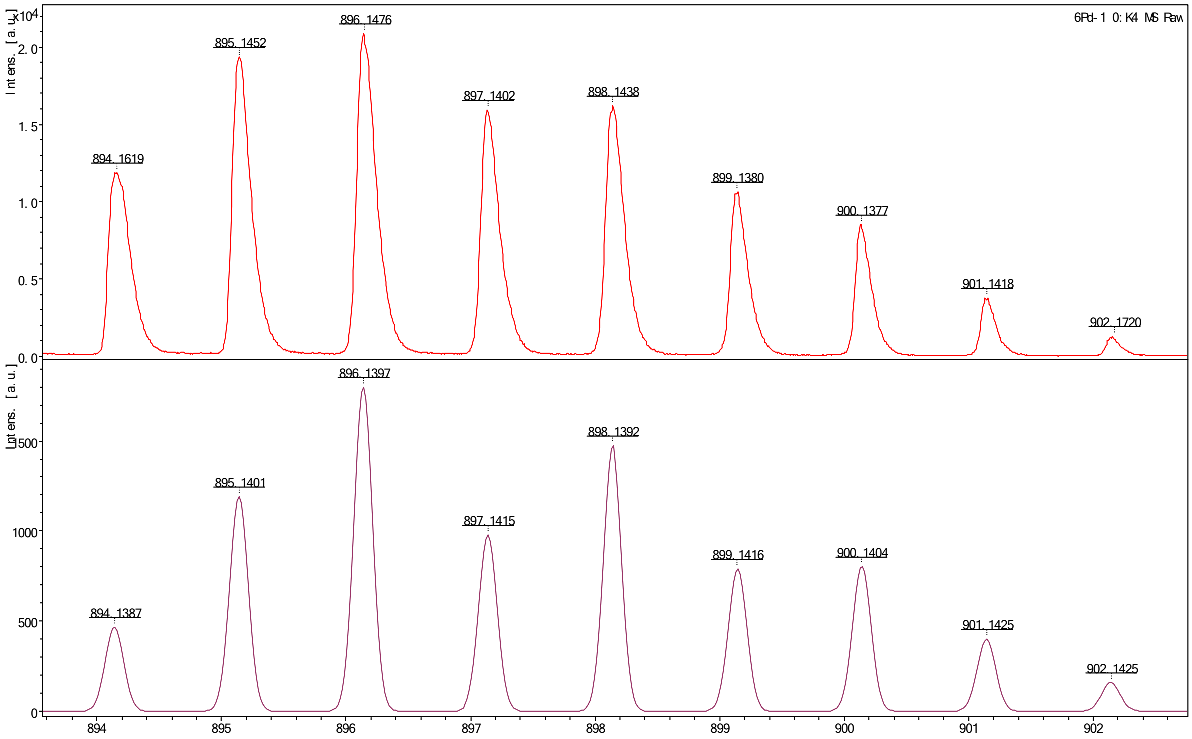

Figure S45. MALDI-TOF-MS spectrum of **6Pd**.

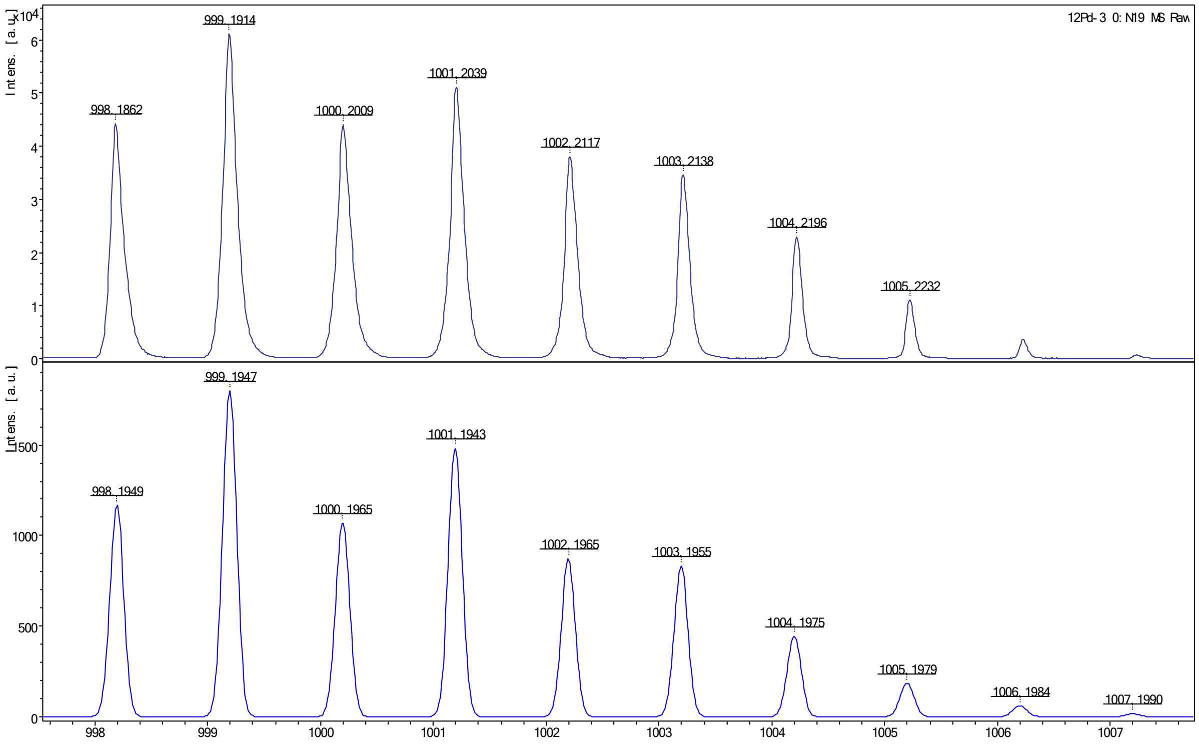

Figure S46. MALDI-TOF-MS spectrum of **7Pd**.

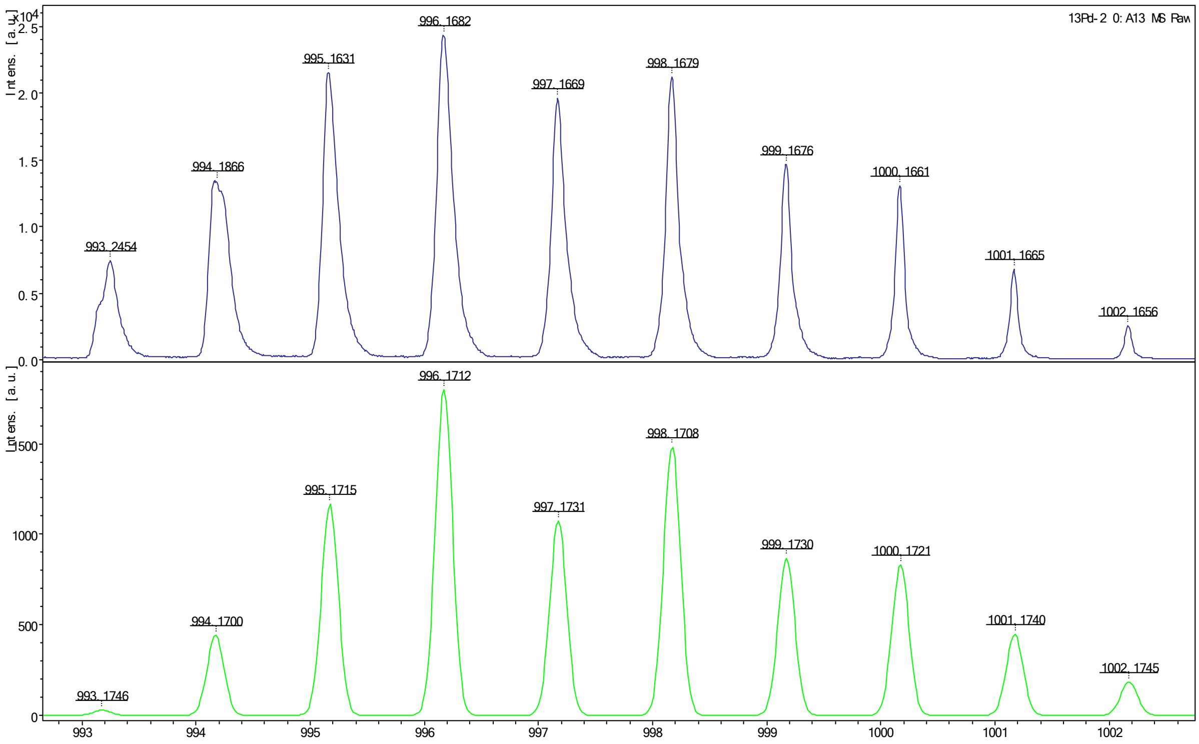

Figure S47. MALDI-TOF-MS spectrum of **8Pd**.

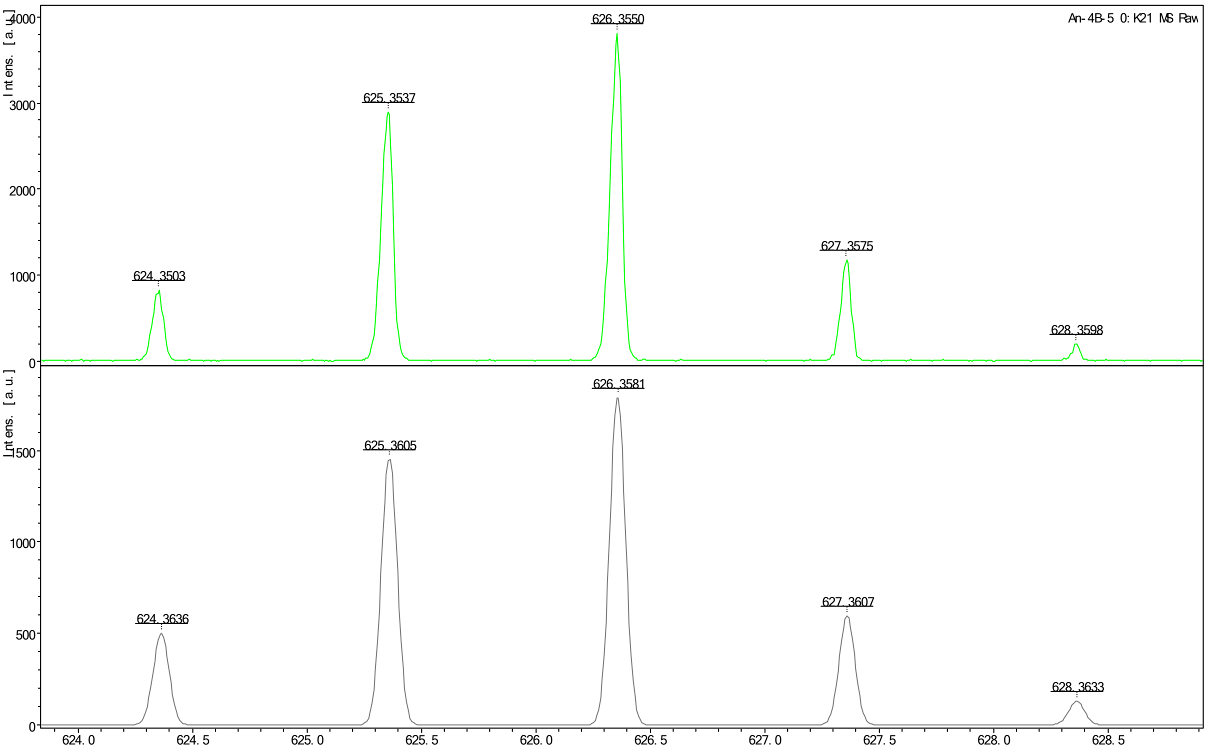

Figure S48. MALDI-TOF-MS spectrum of **1,4,5,8-tetraboryl anthracene**.

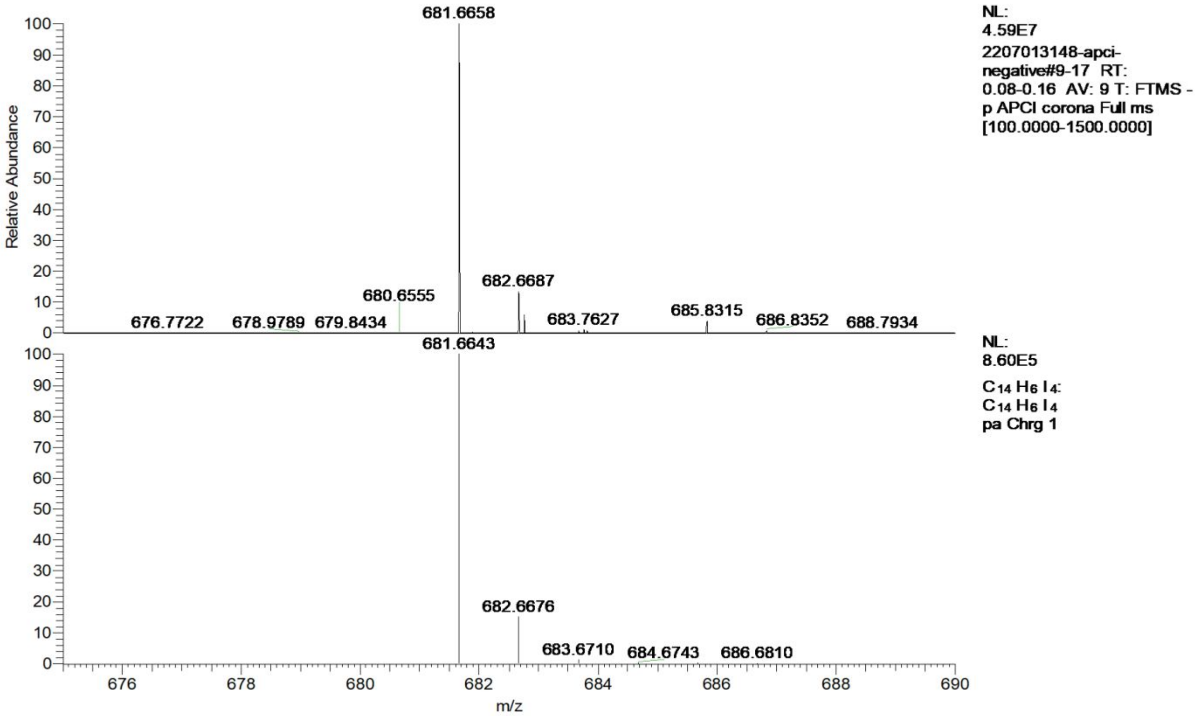

Figure S49. APCI-MS spectrum of **9**.

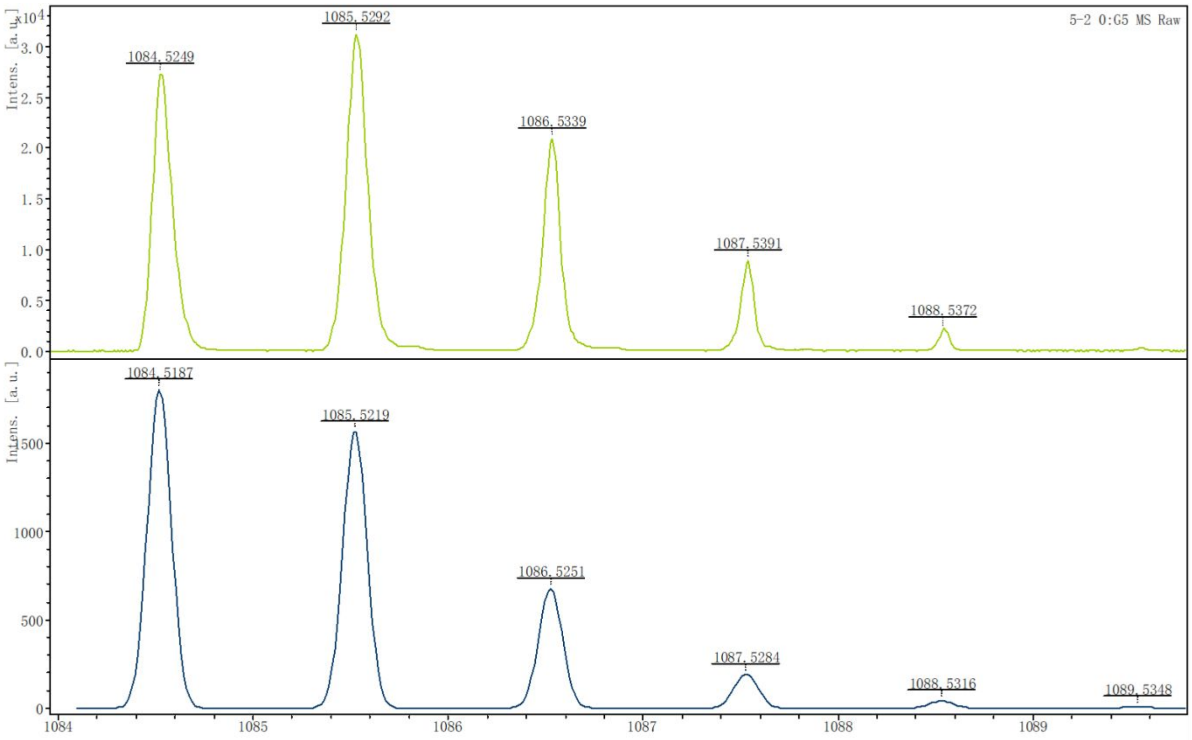

Figure S50. MALDI-TOF-MS spectrum of **10H**.

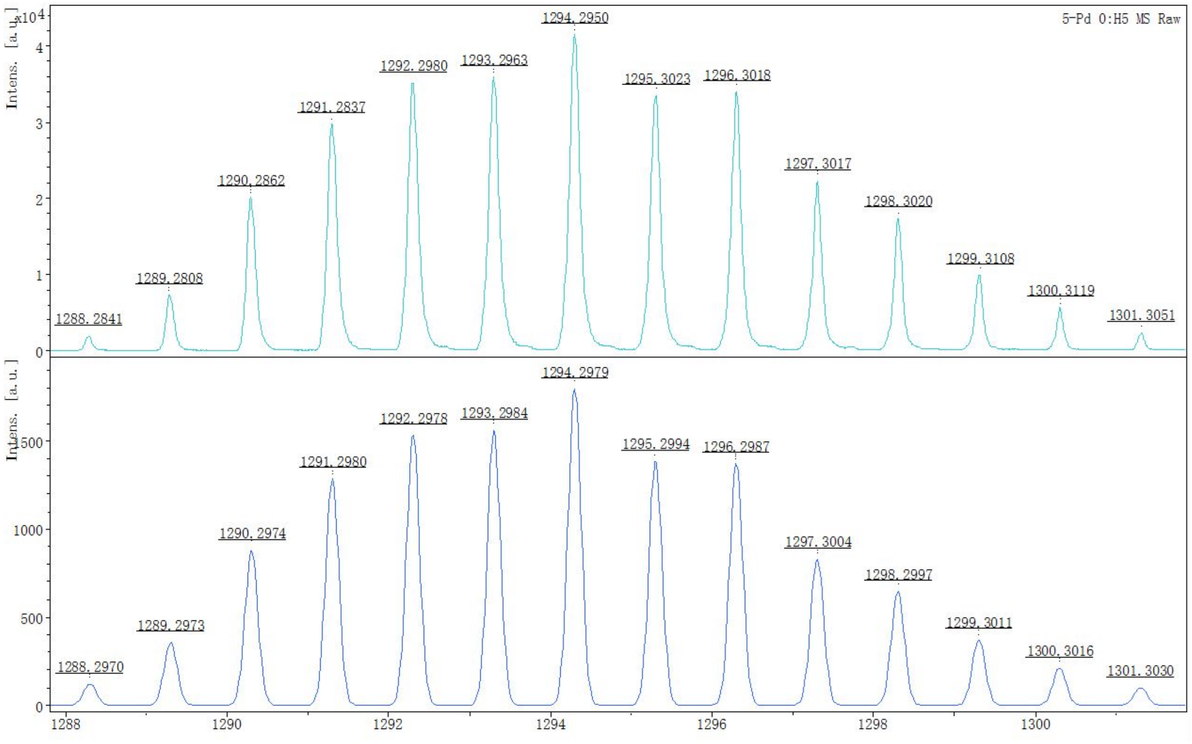

Figure S51. MALDI-TOF-MS spectrum of **10Pd**.

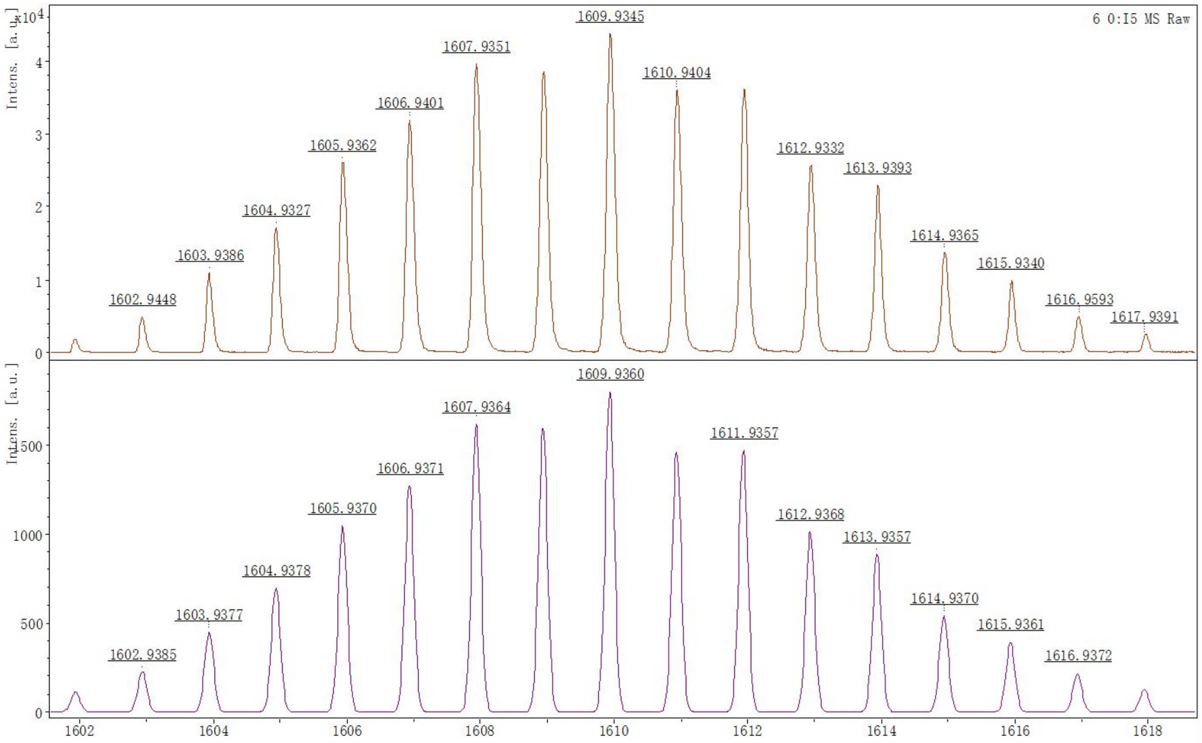

Figure S52. MALDI-TOF-MS spectrum of **11Pd**.

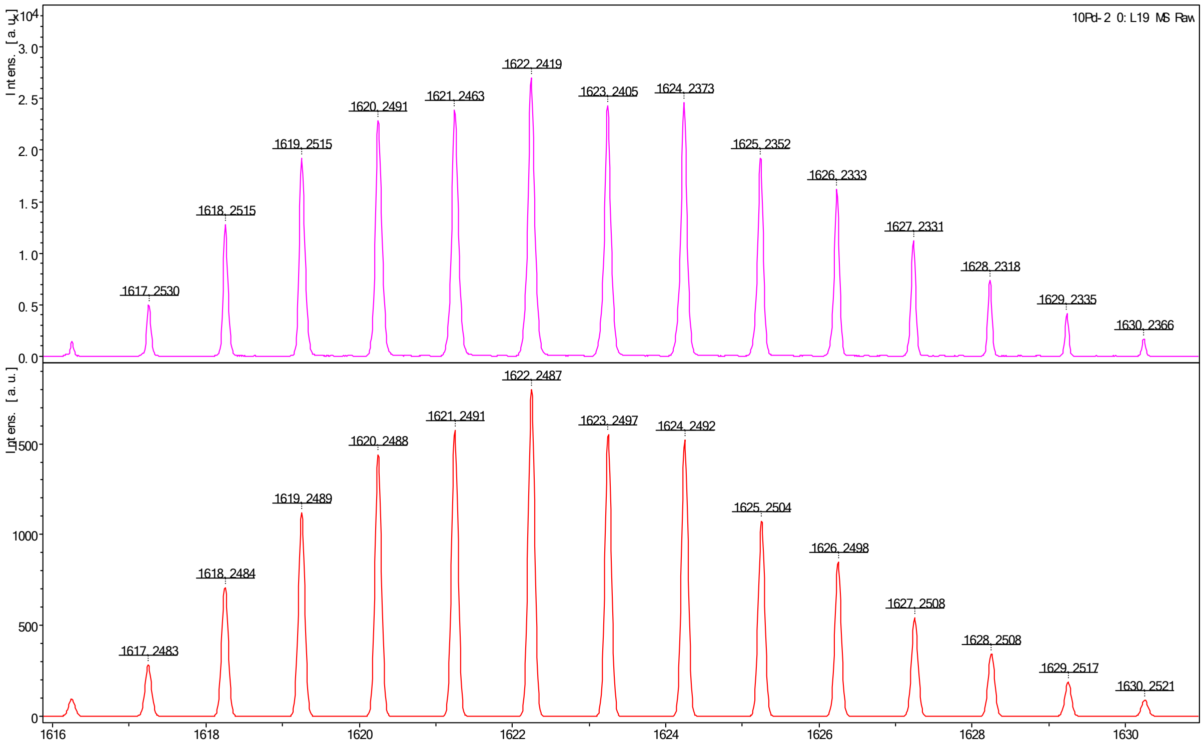

Figure S53. MALDI-TOF-MS spectrum of **12Pd**.

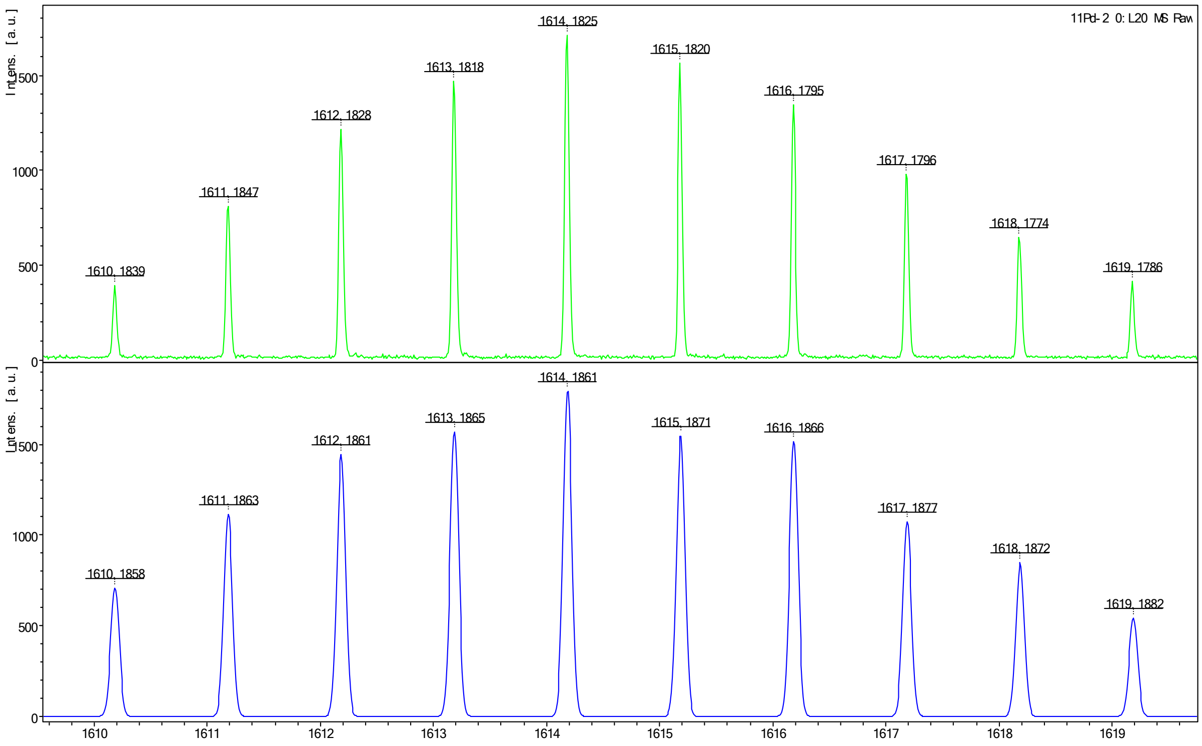

Figure S54. MALDI-TOF-MS spectrum of **13Pd**.

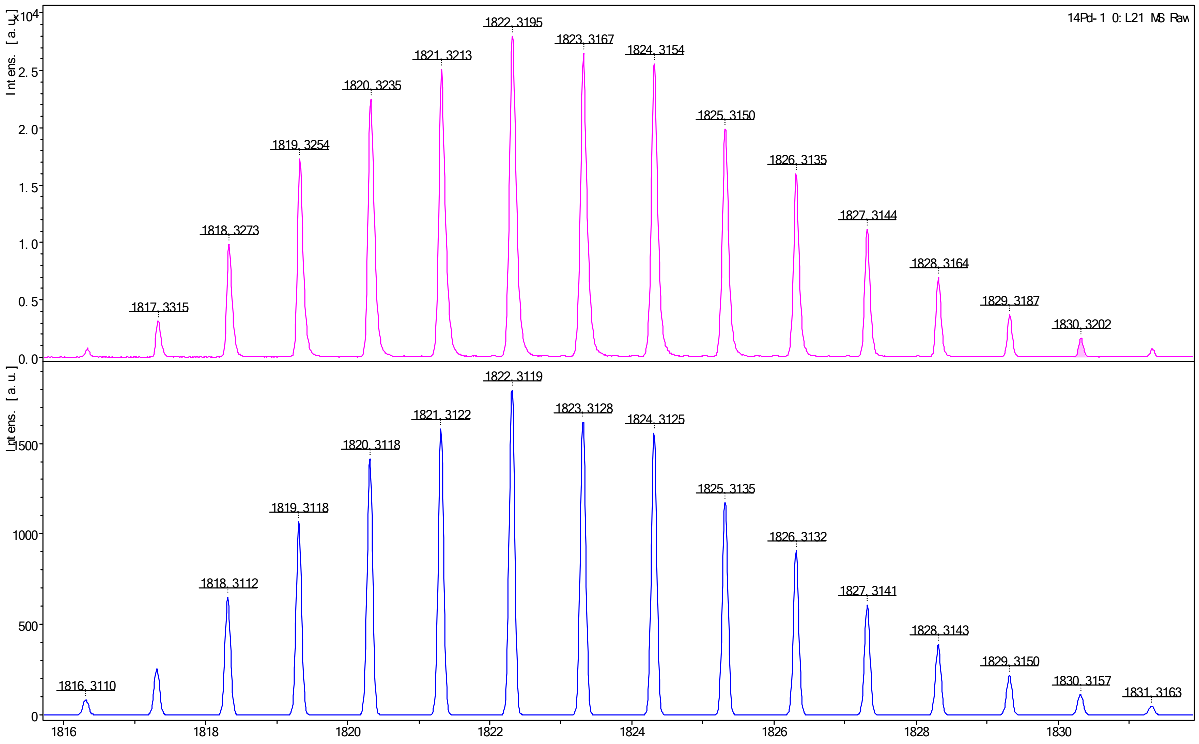

Figure S55. MALDI-TOF-MS spectrum of **14Pd**.

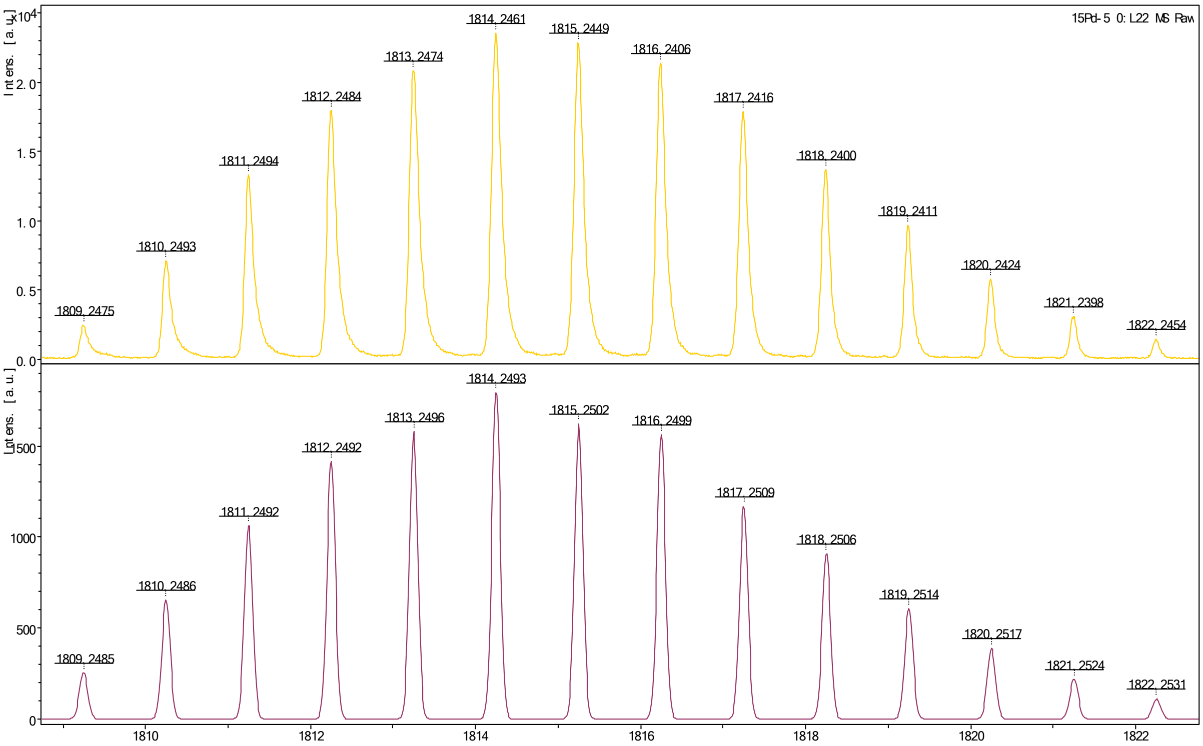

Figure S56. MALDI-TOF-MS spectrum of **15Pd**.

Electrochemical Data

Table S3. CV and DPV of **3H**, **3Pd**, **6Pd**, **8Pd**, **10H**, **10Pd**, **13Pd**, and **15Pd** in CH_2Cl_2 with 0.1 M $n$$Bu_4NPF_6$. Potentials were determined vs ferrocene/ferrocenium ion by differential pulse voltammograms. Potentials [V] vs. ferrocene/ferrocenium ion. Scan rate 0.02 Vs^{-1}. Working electrode: glassy carbon; Counter electrode: Pt wire. Reference electrode: Ag/0.01 M $AgNO_3$.

Sample	E_{Ox2} [V]	E_{Ox1} [V]	E_{Red1} [V]	E_{Red2} [V]	E_{Red3} [V]	E_{Red4} [V]	ΔE [eV] [a]
**3H**	-	0.56	-1.33	-1.68			1.89
**3Pd**	-	0.45	-1.15	-1.76			1.60
**6Pd**		0.51	-0.72	-1.34			1.23
**8Pd**		0.55	-0.65	-1.22			1.20
**10H**	0.57	0.42	-1.11	-1.24			1.53
**10Pd**	0.52	0.22	-0.75	-1.03	-2.19	-2.48	0.97
**13Pd**	0.70	0.37	-0.44	-0.79	-1.55	-1.77	0.81
**15Pd**	0.74	0.42	-0.36	-0.74	-1.56	-1.81	0.78

[a] Electrochemical HOMO–LUMO gaps (ΔE = e ($E_{ox.1}$ - $E_{red.1}$) [eV]).

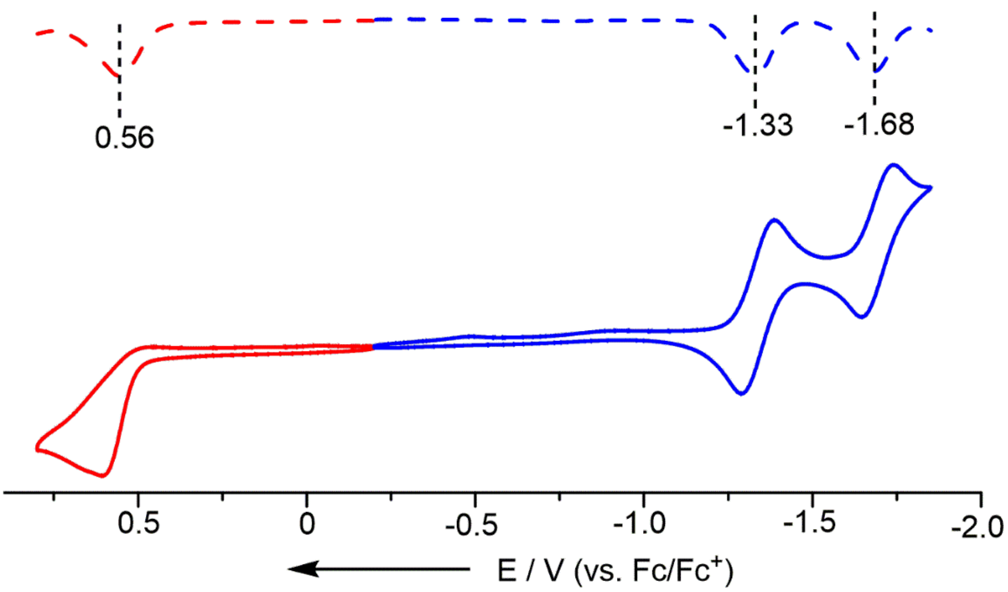

Figure S57. Cyclic voltammogram and differential pulse voltammogram of **3H**.

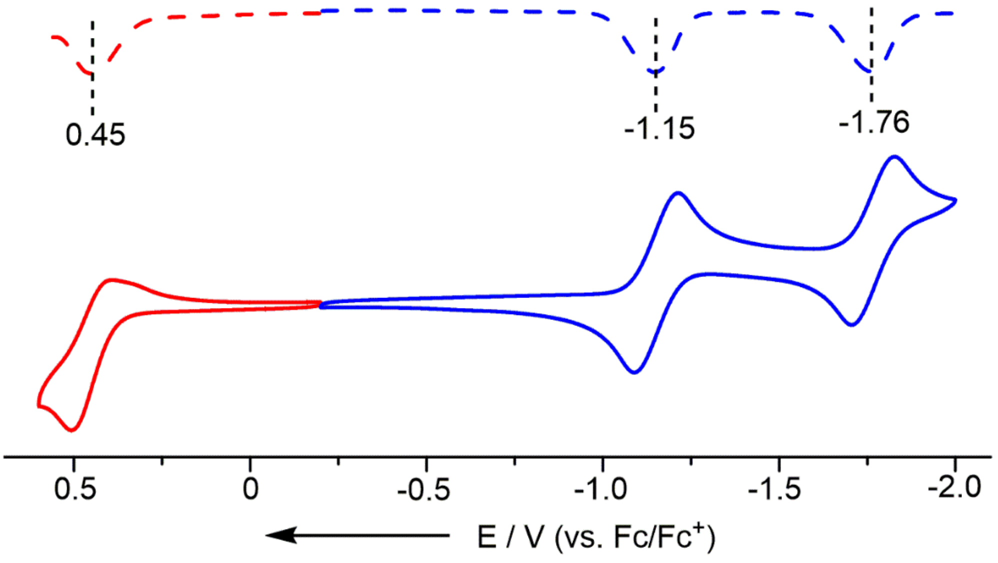

Figure S58. Cyclic voltammogram and differential pulse voltammogram of **3Pd**.

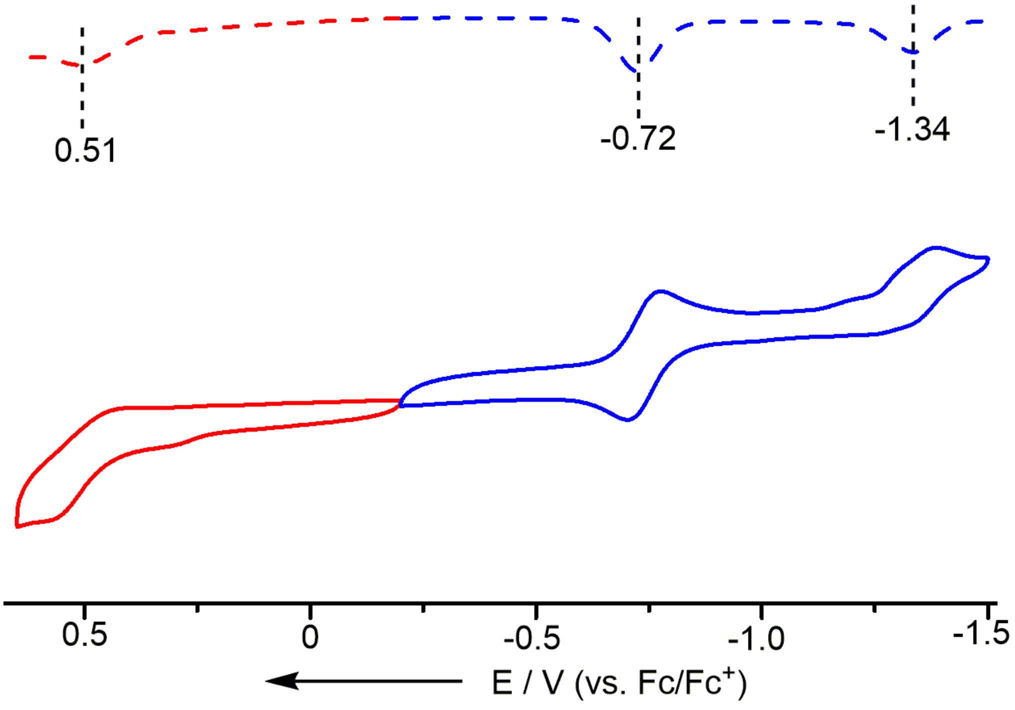

Figure S59. Cyclic voltammogram and differential pulse voltammogram of **6Pd.**

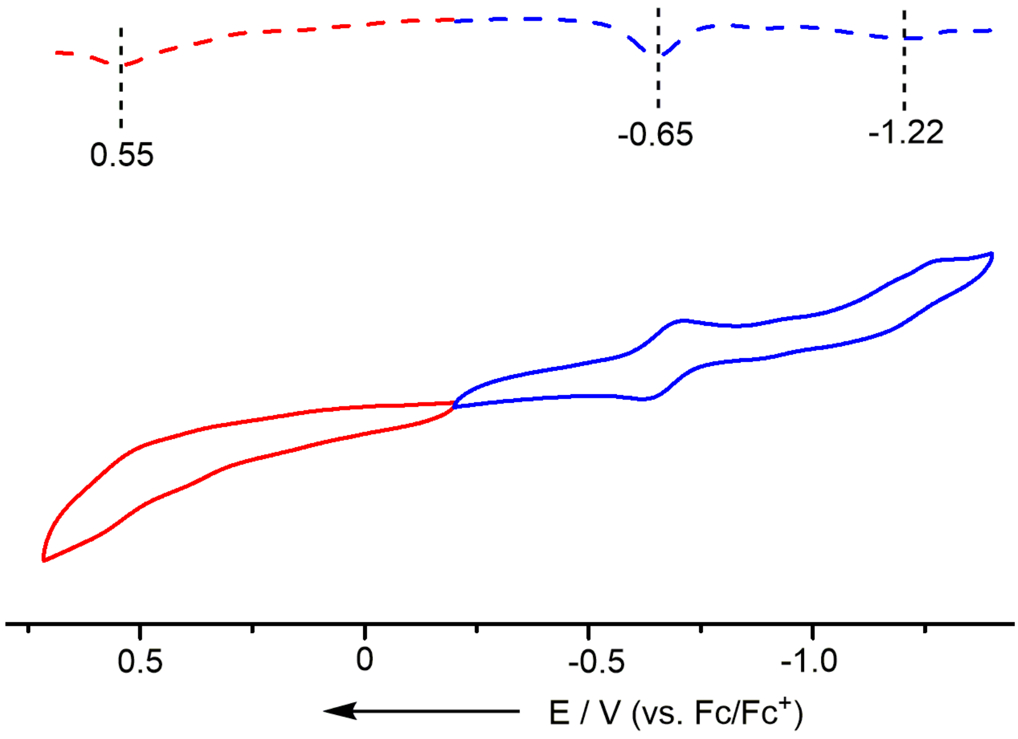

Figure S60. Cyclic voltammogram and differential pulse voltammogram of **8Pd.**

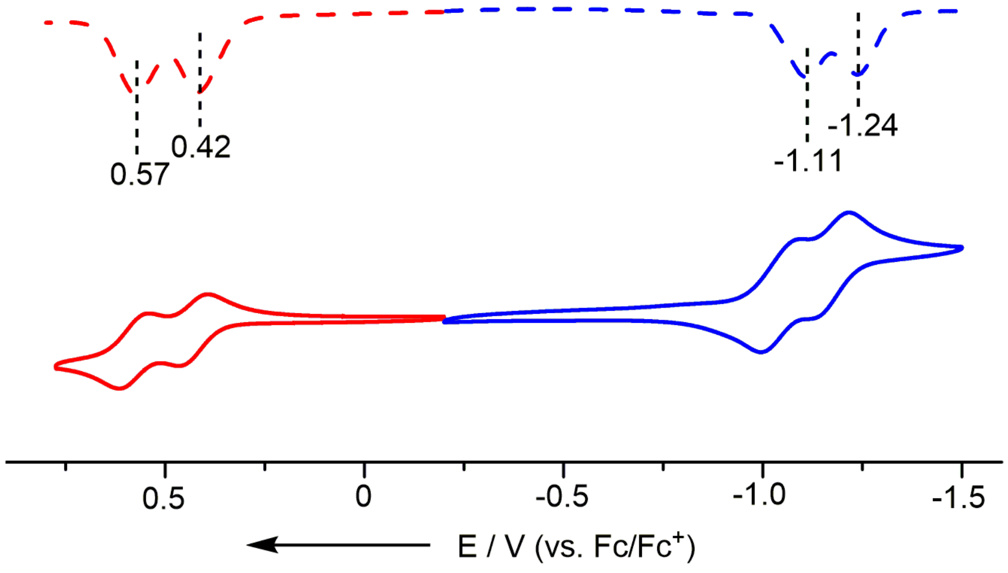

Figure S61. Cyclic voltammogram and differential pulse voltammogram of **10H**.

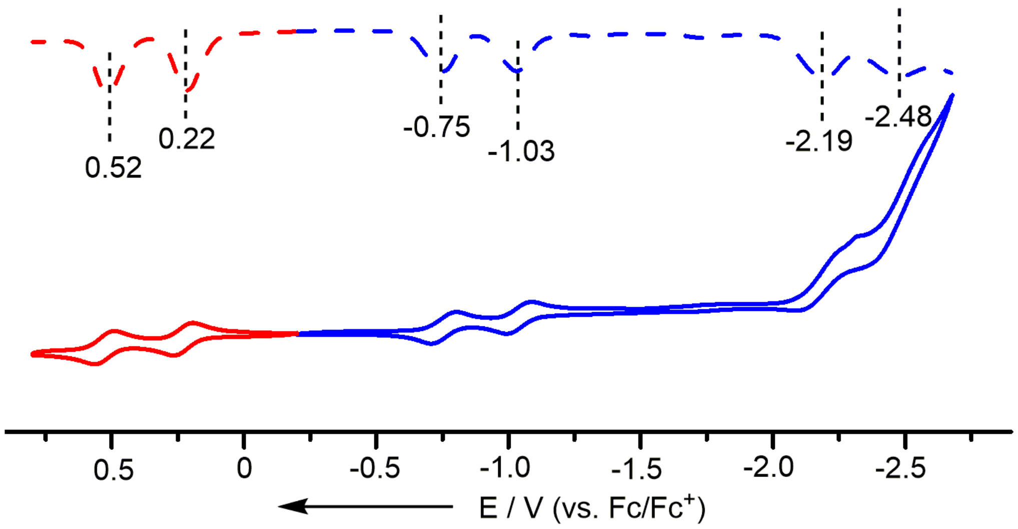

Figure S62. Cyclic voltammogram and differential pulse voltammogram of **10Pd.**

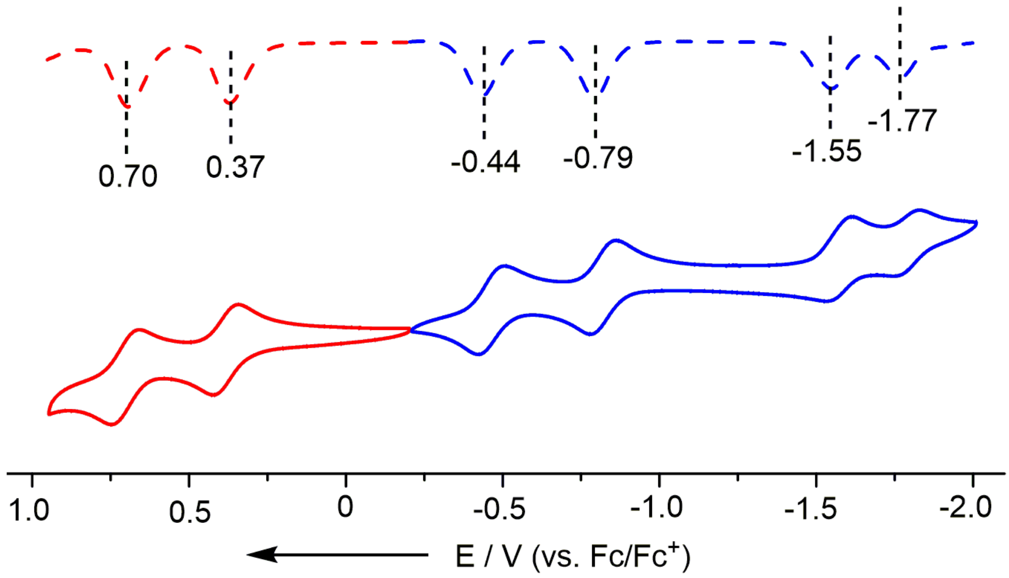

Figure S63. Cyclic voltammogram and differential pulse voltammogram of **13Pd.**

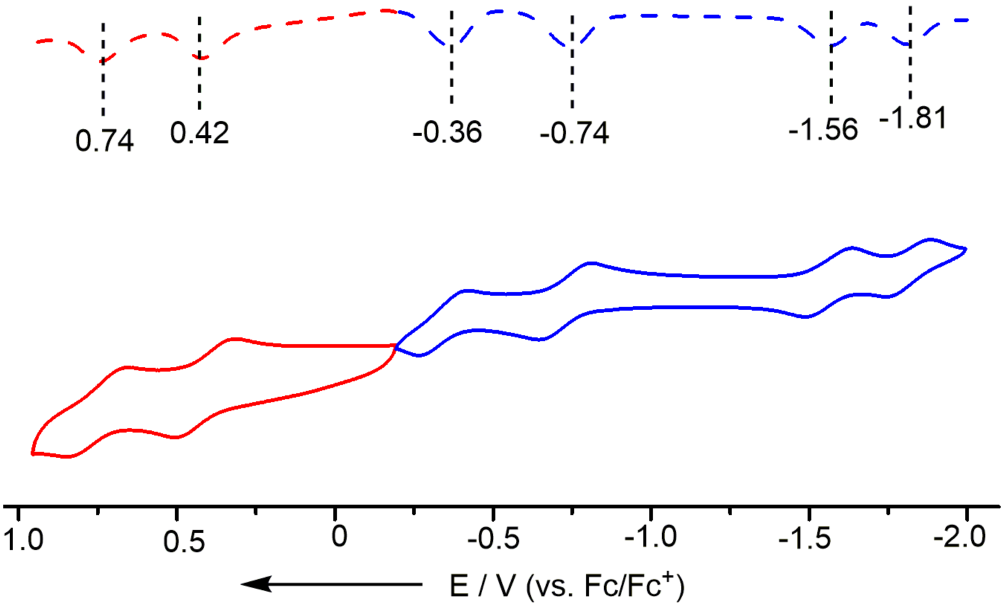

Figure S64. Cyclic voltammogram and differential pulse voltammogram of **15Pd.**

X-Ray Crystal Data

Table S4. Crystal data and structure refinement for **3H**.

Empirical formula	$C_{95}H_{77}Cl_9N_6$	
Formula weight	1621.67	
Temperature	100.01(10) K	
Wavelength	1.54184 Å	
Crystal system	Monoclinic	
Space group	*I*2/*a*	
Unit cell dimensions	a = 39.952(2) Å	
	b = 13.4722(6) Å	β = 99.183(6)°
	c = 29.766(3) Å	
Volume	15815.6(18) Å^3	
Z	8	
Density (calculated)	1.362 Mg/m^3	
Absorption coefficient	3.328 mm^{-1}	
F(000)	6736	
Crystal size	0.3 x 0.1 x 0.01 mm^3	
Theta range for data collection	3.008 to 66.598°	
Index ranges	$-43 \leq h \leq 47$, $-14 \leq k \leq 16$, $-35 \leq l \leq 30$	
Reflections collected	27142	
Independent reflections	13965 [R(int) = 0.0649]	
Completeness to theta = 66.598°	99.8%	
Absorption correction	Semi-empirical from equivalents	
Max. and min. transmission	1.00000 and 0.61733	
Refinement method	Full-matrix least-squares on F^2	
Data / restraints / parameters	13965 / 372 / 1040	
Goodness-of-fit on F^2	1.005	
Final R indices [$I > 2\sigma(I)$]	R_1 = 0.0856, wR_2 = 0.2290	
R indices (all data)	R_1 = 0.1357, wR_2 = 0.2759	
Extinction coefficient	n/a	
Largest diff. peak and hole	1.135 and –0.797 e.Å^{-3}	
CCDC number	2205920	

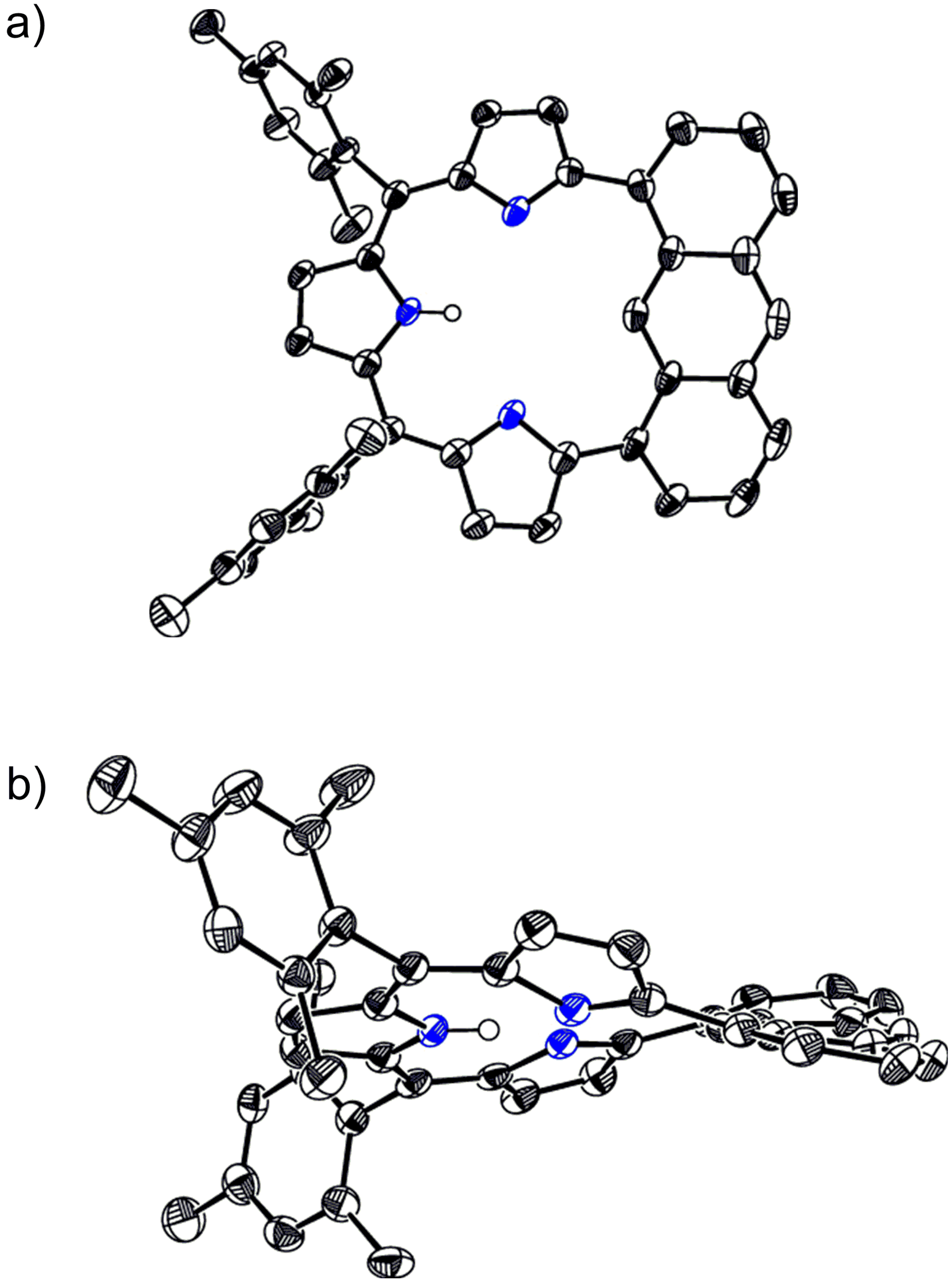

Figure S65. Conformer A of **3H** revealed by X-ray crystal analysis; (a) top view and (b) side view.

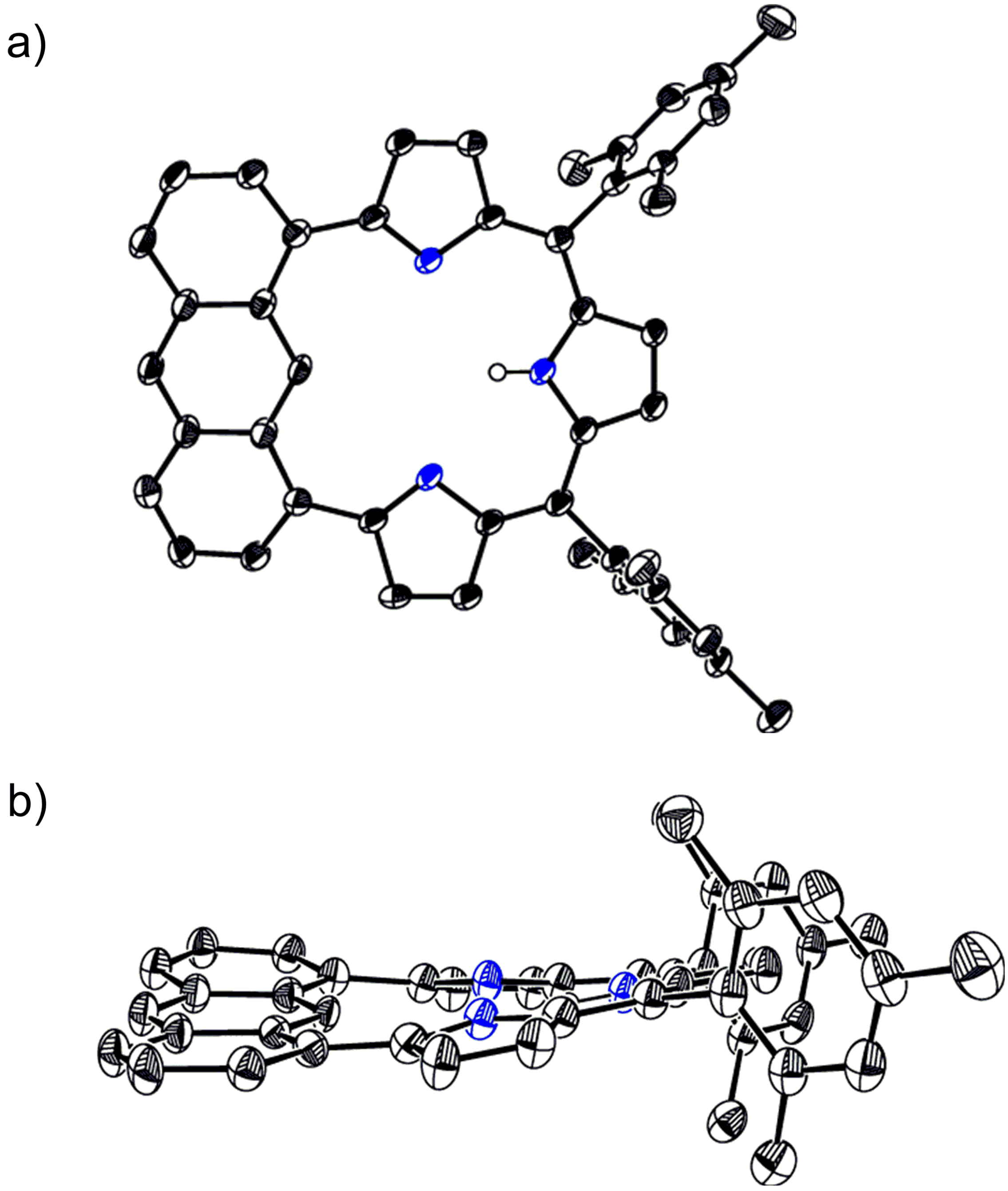

Figure S66. Conformer B of **3H** revealed by X-ray crystal analysis; (a) top view and (b) side view.

Conformer A takes an almost planar conformation with a mean-plane-deviations (MPD) of 0.235(5), the C-N distance of 4.538(6) Å, and the N-N distance of 4.080(6) Å (Figure 1a,b and S65). Conformer B takes a slightly twisted conformation with a larger MPD of 0.556(5) Å, the C-N distance of 4.455(6) Å, and the N-N distance of 4.014(6) Å (Figure S66).

Table S5. Crystal data and structure refinement for **3Pd**.

Empirical formula	$C_{46}H_{35}N_3Pd$	
Formula weight	736.17	
Temperature	100.01(10) K	
Wavelength	1.54184 Å	
Crystal system	Monoclinic	
Space group	$P2_1/c$	
Unit cell dimensions	a = 20.7148(6) Å	
	b = 7.3461(2) Å	β = 92.248(3)°
	c = 24.0336(8) Å	
Volume	3654.5(2) $Å^3$	
Z	4	
Density (calculated)	1.338 Mg/m^3	
Absorption coefficient	4.365 mm^{-1}	
$F(000)$	1512	
Crystal size	0.2 x 0.1 x 0.01 mm^3	
Theta range for data collection	3.681 to 66.591°	
Index ranges	$-24 \le h \le 22$, $-8 \le k \le 8$, $-21 \le l \le 28$	
Reflections collected	12756	
Independent reflections	6445 [R(int) = 0.0419]	
Completeness to theta = 66.591°	99.9%	
Absorption correction	Semi-empirical from equivalents	
Max. and min. transmission	1.00000 and 0.46541	
Refinement method	Full-matrix least-squares on F^2	
Data / restraints / parameters	6445 / 0 / 457	
Goodness-of-fit on F^2	1.050	
Final R indices [$I > 2\sigma(I)$]	R_1 = 0.0431, wR_2 = 0.1054	
R indices (all data)	R_1 = 0.0522, wR_2 = 0.1115	
Extinction coefficient	n/a	
Largest diff. peak and hole	0.642 and –1.121 e.$Å^{-3}$	
CCDC number	2205921	

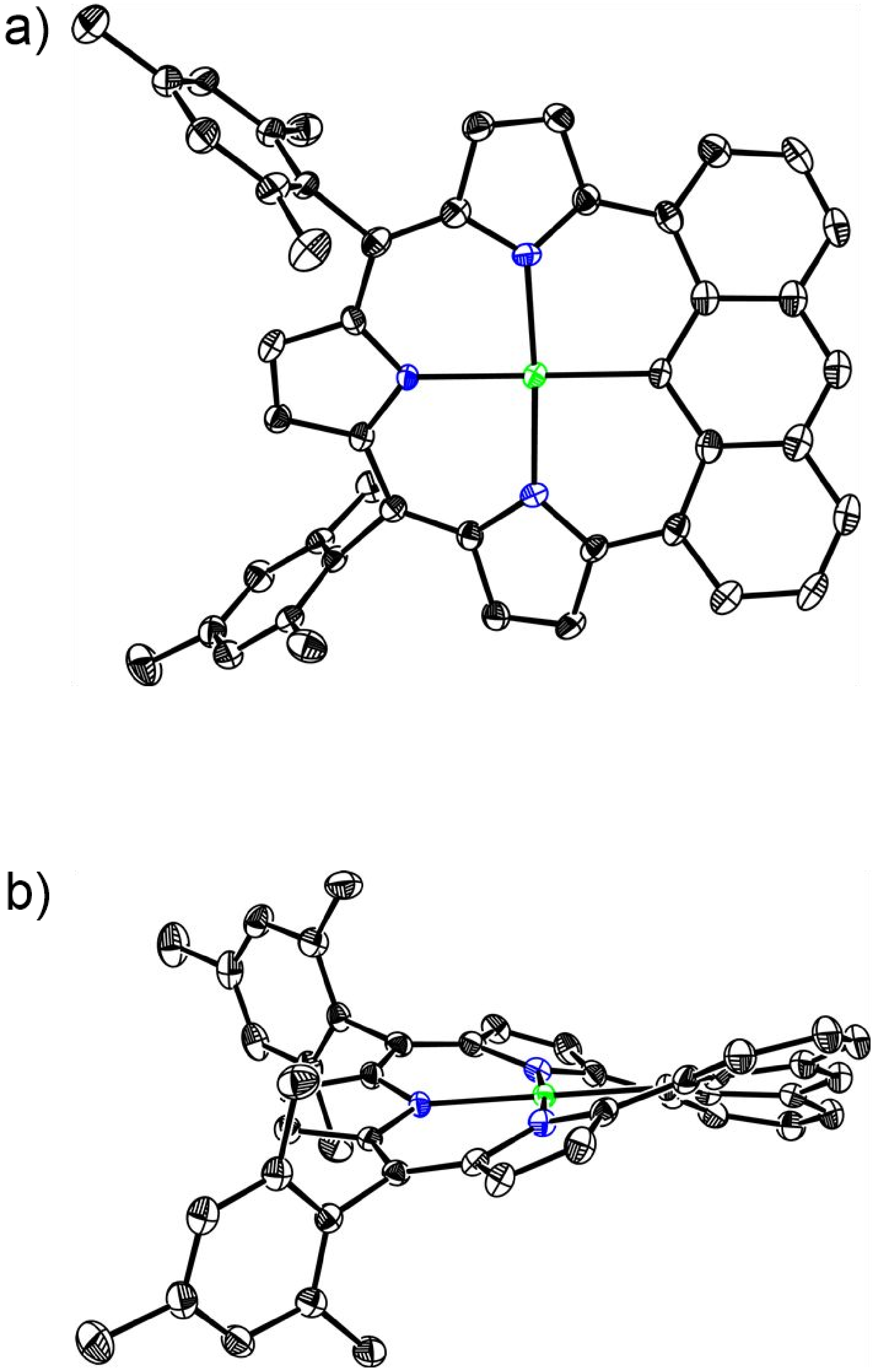

Figure S67. X-ray crystal structure of **3Pd**: (a) top view and (b) side view.

Table S6. Crystal data and structure refinement for **6Pd**.

Empirical formula	$C_{54}H_{35}N_3PdS_2$	
Formula weight	896.37	
Temperature	99.97(12) K	
Wavelength	1.54184 Å	
Crystal system	Monoclinic	
Space group	$C2/c$	
Unit cell dimensions	a = 22.6642(8) Å	
	b = 12.9627(3) Å	β = 106.319(4)°
	c = 13.3000(5) Å	
Volume	3750.0(2) $Å^3$	
Z	4	
Density (calculated)	1.588 Mg/m^3	
Absorption coefficient	5.391 mm^{-1}	
F(000)	1832	
Crystal size	0.15 x 0.02 x 0.02 mm^3	
Theta range for data collection	3.970 to 66.599°.	
Index ranges	$-26 \leq h \leq 26$, $-15 \leq k \leq 14$, $-8 \leq l \leq 15$	
Reflections collected	6287	
Independent reflections	3319 [R(int) = 0.0189]	
Completeness to theta = 66.599°	100.0%	
Absorption correction	Semi-empirical from equivalents	
Max. and min. transmission	1.00000 and 0.70004	
Refinement method	Full-matrix least-squares on F^2	
Data / restraints / parameters	3319 / 0 / 276	
Goodness-of-fit on F^2	1.064	
Final R indices [$I > 2\sigma(I)$]	R_1 = 0.0378, wR_2 = 0.0986	
R indices (all data)	R_1 = 0.0436, wR_2 = 0.1026	
Extinction coefficient	n/a	
Largest diff. peak and hole	1.273 and –0.648 e.$Å^{-3}$	
CCDC number	2205922	

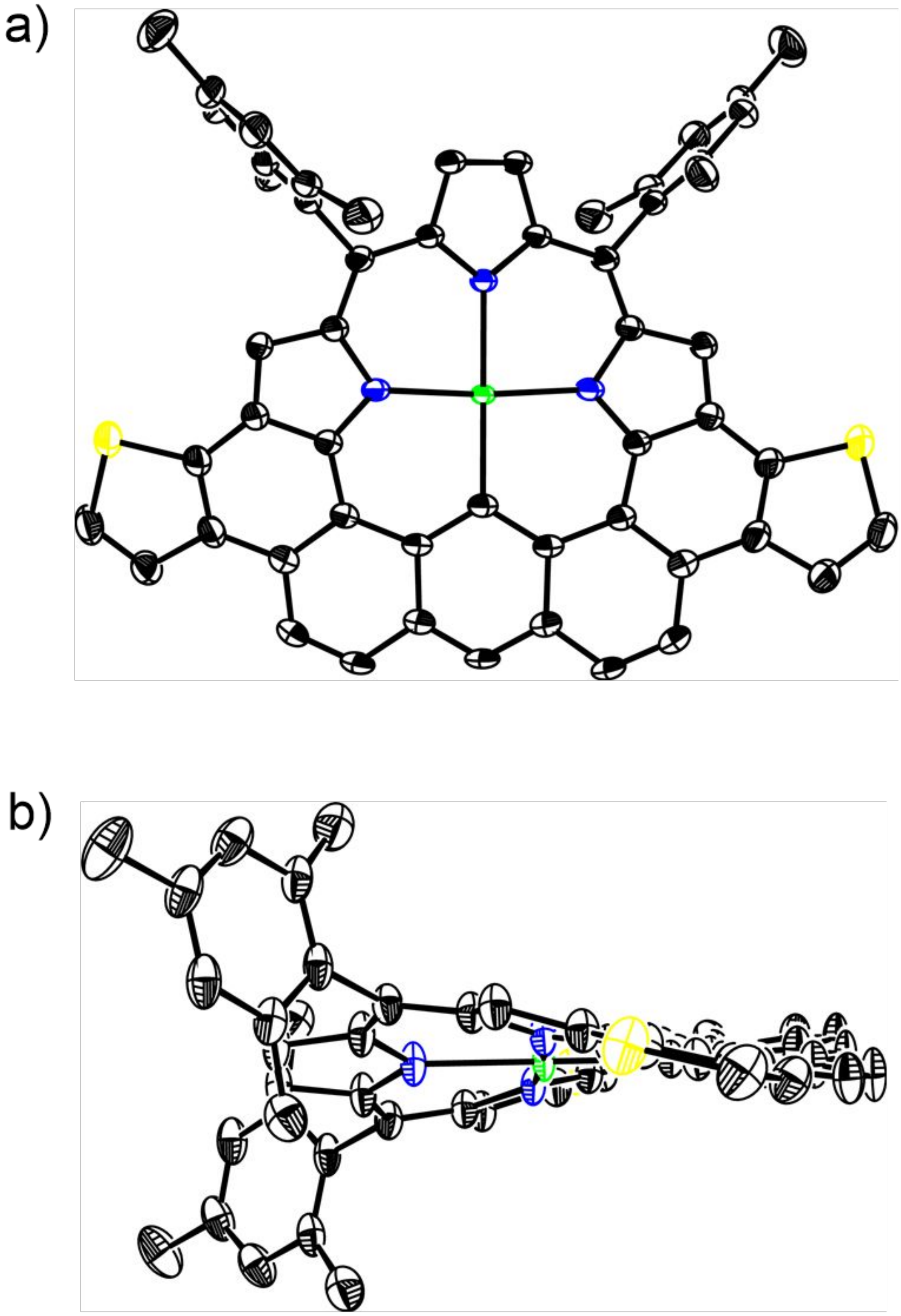

Figure S68. X-ray crystal structure of **6Pd** (a) Top view and (b) side view.

Table S7. Crystal data and structure refinement for **10Pd**.

Empirical formula	$C_{78}H_{60}N_6Pd_2$	
Formula weight	1294.12	
Temperature	100.01(10) K	
Wavelength	1.54184 Å	
Crystal system	Triclinic	
Space group	*P*-1	
Unit cell dimensions	a = 16.4578(4) Å	α = 90.341(2)°
	b = 20.3648(4) Å	β = 105.208(2)°
	c = 22.6481(6) Å	γ = 91.925(2)°
Volume	7319.9(3) $Å^3$	
Z	4	
Density (calculated)	1.174 Mg/m^3	
Absorption coefficient	4.289 mm^{-1}	
F(000)	2648	
Crystal size	0.1 x 0.02 x 0.02 mm^3	
Theta range for data collection	2.022 to 66.600°.	
Index ranges	$-19 \leq h \leq 19$, $-15 \leq k \leq 24$, $-26 \leq l \leq 26$	
Reflections collected	51203	
Independent reflections	25870 [*R*(int) = 0.0585]	
Completeness to theta = 66.600°	100.0%	
Absorption correction	Semi-empirical from equivalents	
Max. and min. transmission	1.00000 and 0.71551	
Refinement method	Full-matrix least-squares on F^2	
Data / restraints / parameters	25870 / 901 / 1745	
Goodness-of-fit on F^2	1.014	
Final *R* indices [$I > 2\sigma(I)$]	R_1 = 0.0647, wR_2 = 0.1571	
R indices (all data)	R_1 = 0.0843, wR_2 = 0.1698	
Extinction coefficient	n/a	
Largest diff. peak and hole	1.672 and −0.972 e.$Å^{-3}$	
CCDC number	2205923	

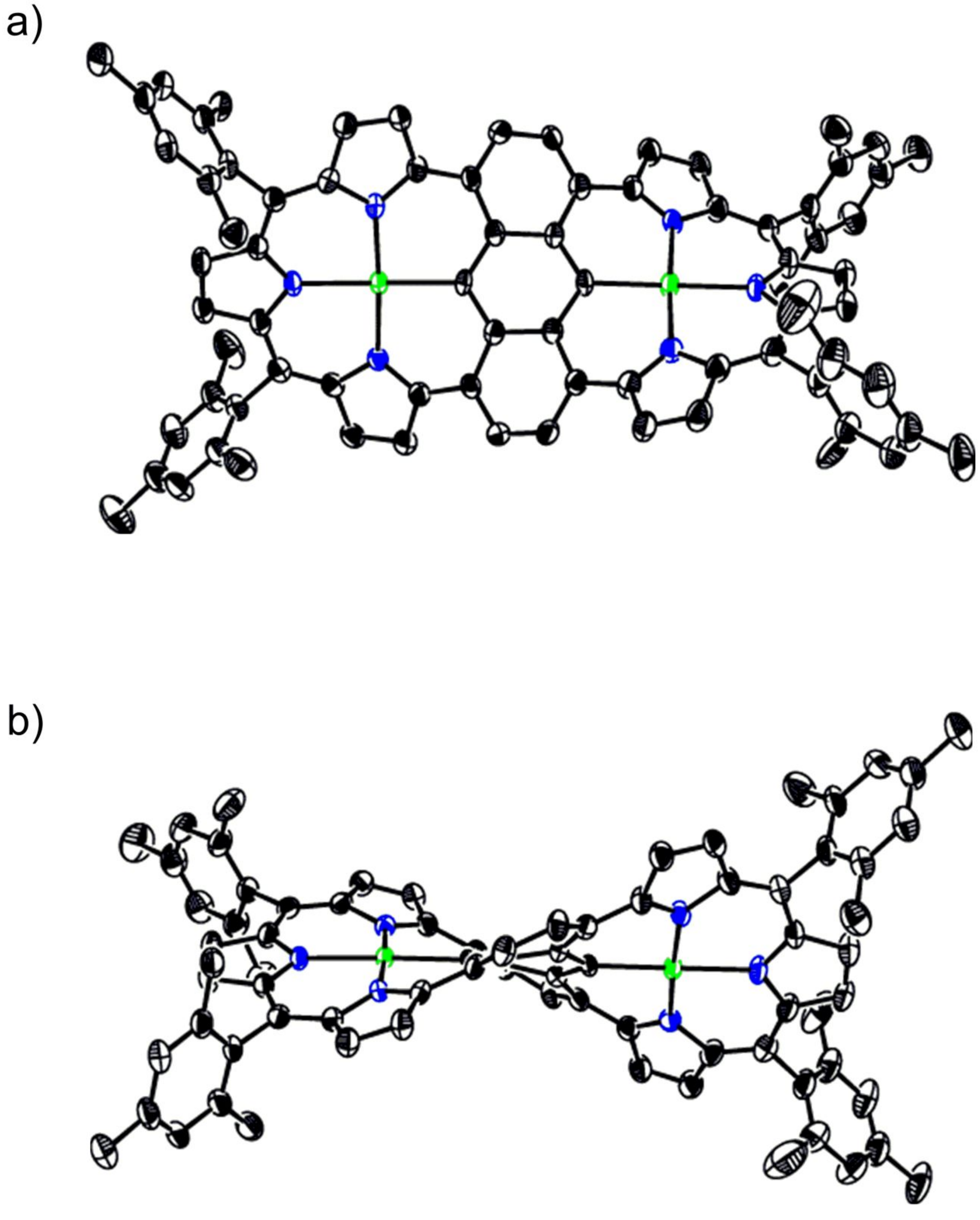

Figure S69. One conformer of **10Pd** revealed X-ray crystal analysis: (a) top view and (b) side view.

a)

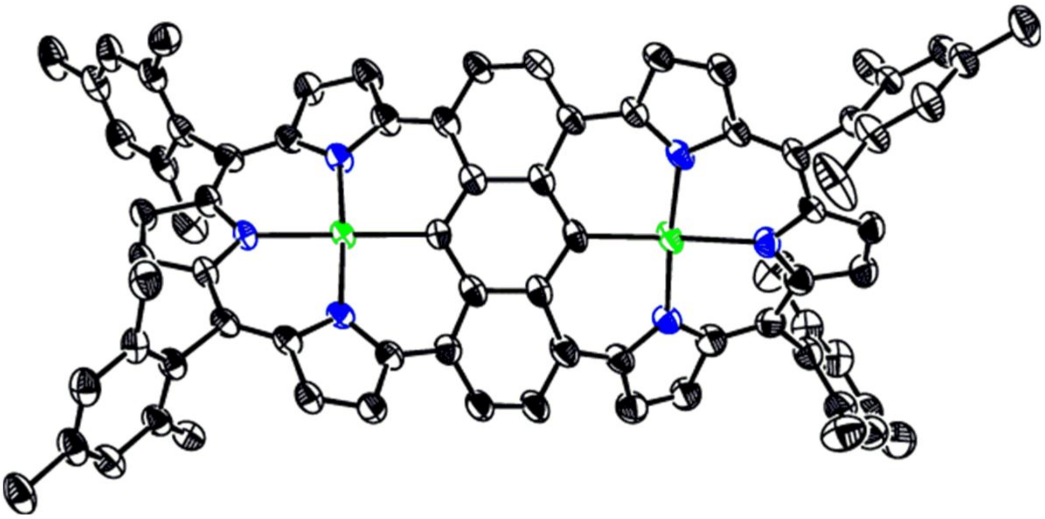

b)

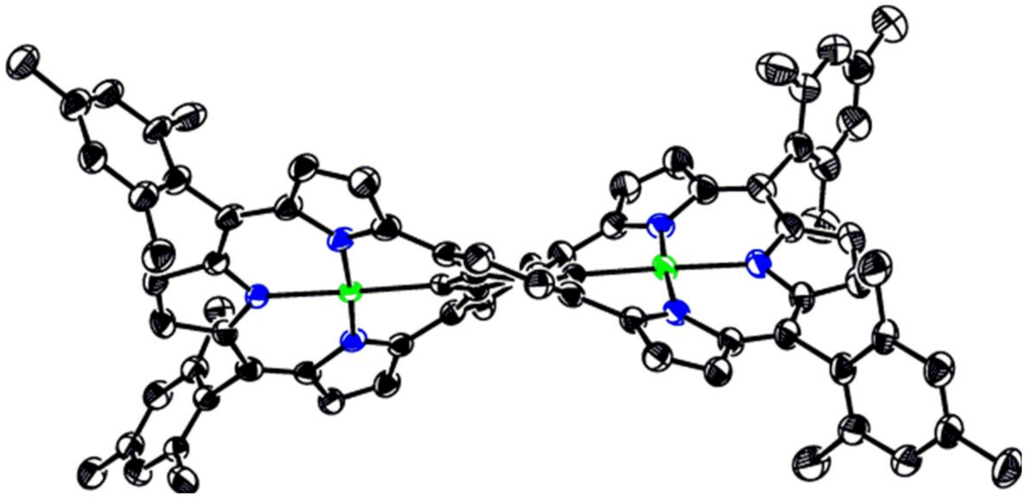

Figure S70. The other conformer of **10Pd** revealed X-ray crystal analysis: (a) top view and (b) side view.

Table S8. Crystal data and structure refinement for **13Pd**.

Empirical formula	$C_{94}H_{60}N_6Pd_2S_4$	
Formula weight	1614.52	
Temperature	100.01(10) K	
Wavelength	1.54184 Å	
Crystal system	Monoclinic	
Space group	$P2_1/c$	
Unit cell dimensions	a = 26.2609(7) Å	
	b = 22.4876(6) Å	β = 103.732(4)
	c = 38.3506(19) Å	
Volume	22000.4(14) Å^3	
Z	8	
Density (calculated)	0.975 Mg/m^3	
Absorption coefficient	3.629 mm^{-1}	
F(000)	6576	
Crystal size	0.3 x 0.3 x 0.001 mm^3	
Theta range for data collection	2.372 to 66.599°.	
Index ranges	$-31 \leq h \leq 27$, $-22 \leq k \leq 26$, $-45 \leq l \leq 42$	
Reflections collected	81679	
Independent reflections	38807 [R(int) = 0.1065]	
Completeness to theta = 66.599°	99.9 %	
Absorption correction	Semi-empirical from equivalents	
Max. and min. transmission	1.00000 and 0.45408	
Refinement method	Full-matrix least-squares on F^2	
Data / restraints / parameters	38807 / 1075 / 2067	
Goodness-of-fit on F^2	1.038	
Final R indices [$I > 2\sigma(I)$]	R_1 = 0.1000, wR_2 = 0.2575	
R indices (all data)	R_1 = 0.1559, wR_2 = 0.2949	
Extinction coefficient	n/a	
Largest diff. peak and hole	1.670 and −0.718 e.Å^{-3}	
CCDC number	2205924	

a)

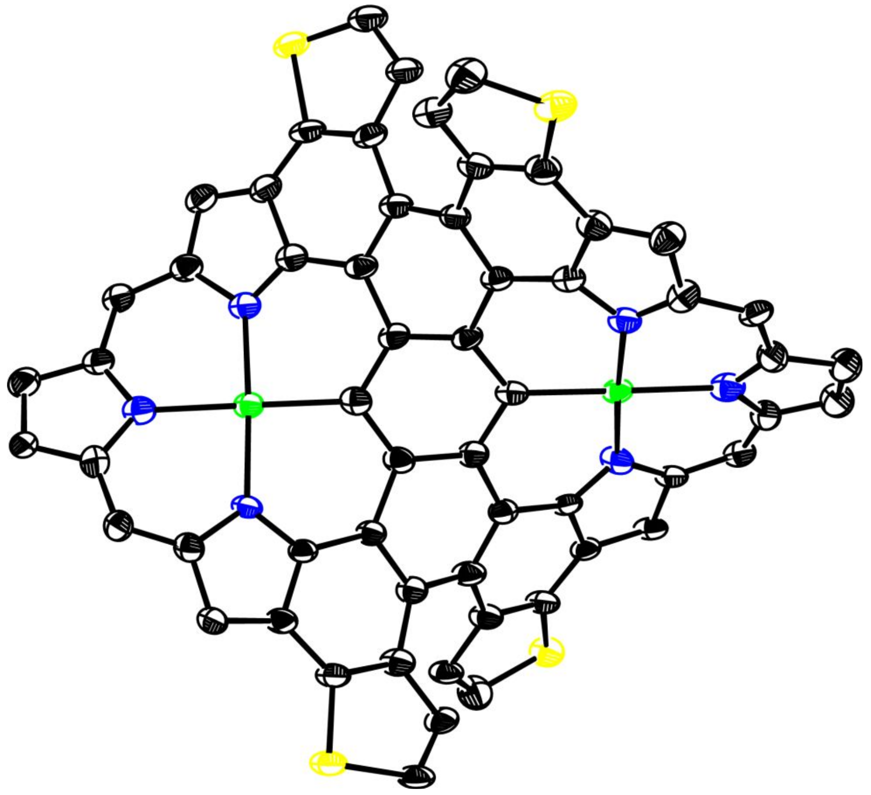

b)

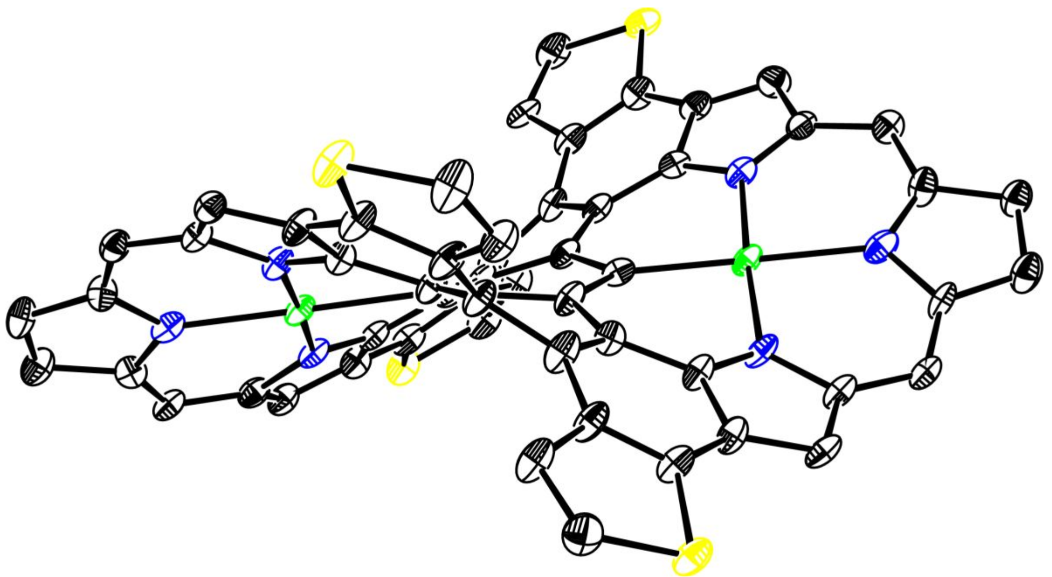

Figure S71. X-ray crystal structure of **13Pd** (a) Top view and (b) side view. (2,4,6-trimethylphenyl groups are omitted for clarity.

Table S9. Crystal data and structure refinement for **15Pd**.

Empirical formula	$C_{110}H_{68}N_6Pd_2S_4$	
Formula weight	1814.74	
Temperature	100.01(10) K	
Wavelength	1.54184 Å	
Crystal system	Monoclinic	
Space group	*P*2/*c*	
Unit cell dimensions	a = 19.1138(4) Å	
	b = 18.0359(5) Å	β = 100.049(3)°
	c = 35.5480(11) Å	
Volume	12066.6(6) $Å^3$	
Z	4	
Density (calculated)	0.999 Mg/m^3	
Absorption coefficient	3.356 mm^{-1}	
F(000)	3704	
Crystal size	0.3 x 0.02 x 0.02 mm^3	
Theta range for data collection	2.348 to 66.601°	
Index ranges	$-22 \le h \le 22$, $-21 \le k \le 21$, $-42 \le l \le 32$	
Reflections collected	45517	
Independent reflections	21321 [*R*(int) = 0.0387]	
Completeness to theta = 66.601°	99.9%	
Absorption correction	Semi-empirical from equivalents	
Max. and min. transmission	1.00000 and 0.33543	
Refinement method	Full-matrix least-squares on F^2	
Data / restraints / parameters	21321 / 0 / 1111	
Goodness-of-fit on F^2	1.031	
Final *R* indices [$I > 2\sigma(I)$]	R_1 = 0.0470, wR_2 = 0.1209	
R indices (all data)	R_1 = 0.0604, wR_2 = 0.1288	
Extinction coefficient	n/a	
Largest diff. peak and hole	0.900 and –0.775 e.$Å^{-3}$	
CCDC number	2205925	

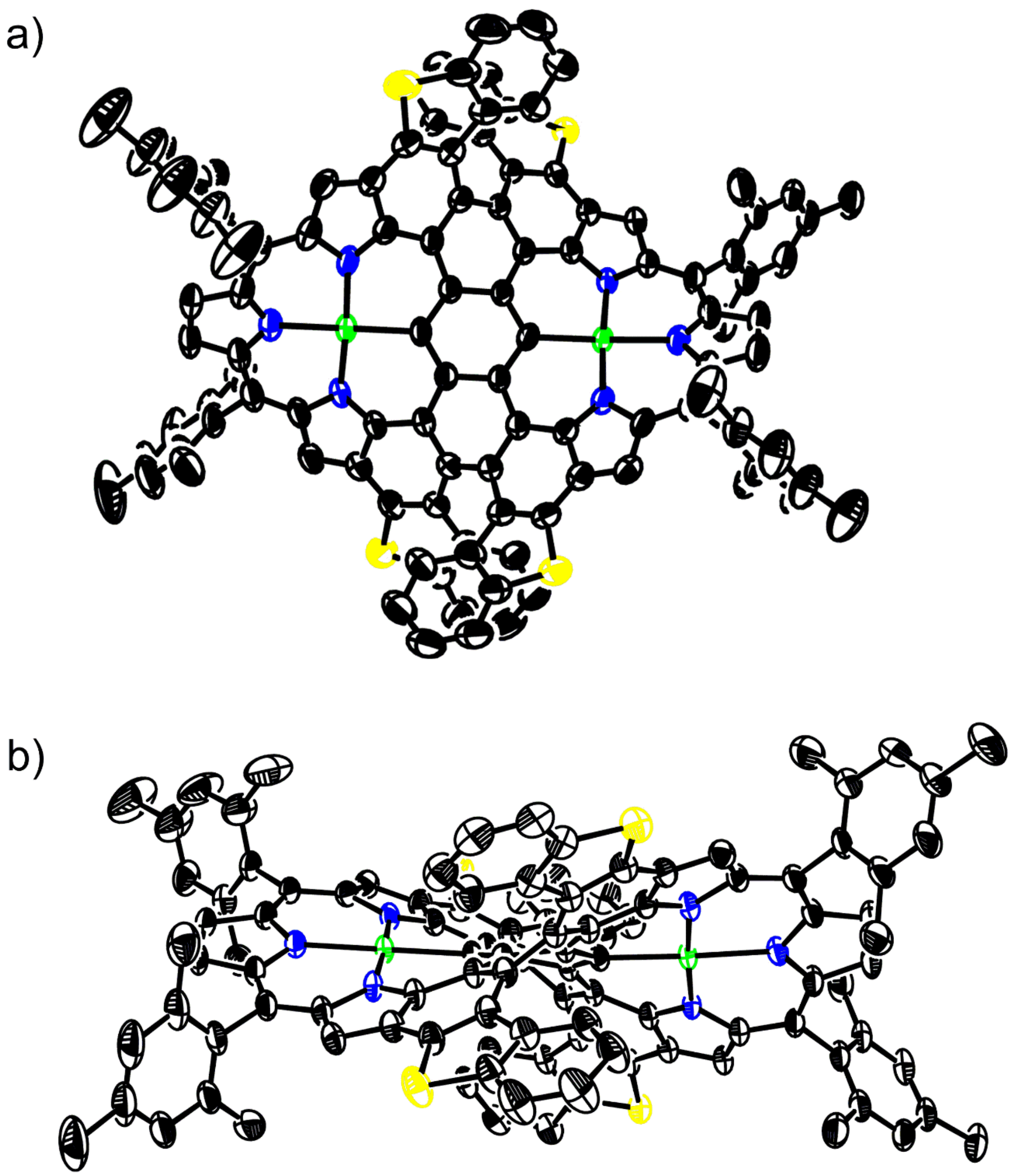

Figure S72. X-ray crystal structure of **15Pd**: (a) Top view and (b) side view.

DFT/Calculations

All calculations were carried out using the Gaussian 09 program.[1] All structures were fully optimized without any symmetry restriction. All geometries (except **8Pd and 10H**) were optimized with the crystal structures as the starting structure at the density functional theory (DFT) method with restricted B3LYP (Becke's three parameter hybrid exchange functionals and the Lee-Yang-Parr correlation functional) level,[2,3] employing 6-31G(d) basis set. Additionally, in order to quantify the impact of the geometric change on aromaticity, the nucleus independent chemical shifts (NICS) values were obtained with the GIAO method based on the final optimized structures, and results for NICS(0) were collected. Anisotropy of the induced current density (ACID) plots were obtained with the CSGT method based on the final optimized structures. Excitation energies and oscillator strengths were calculated with the TD-SCF method at the B3LYP level.

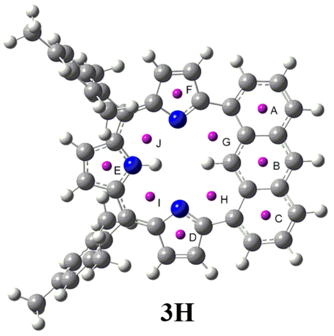

3H

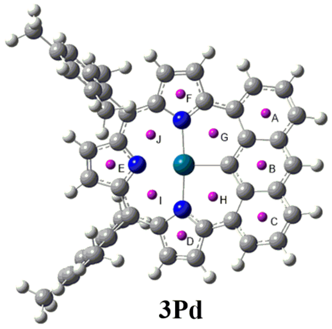

3Pd

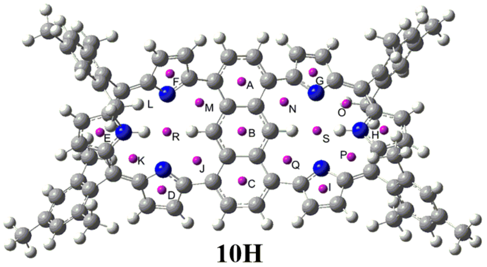

10H

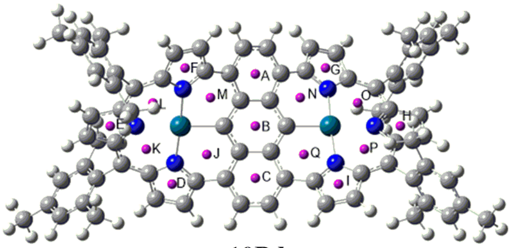

10Pd

	3H	3Pd	10H	10Pd
**A**	-8.0900	-7.1368	-3.3957	2.3486
**B**	-10.9123	-8.2155	-7.4755	0.1068
**C**	-7.6113	-7.1695	-3.3935	2.3507
**D**	-2.5084	-2.3887	-2.2644	-0.5966
**E**	-9.5056	-4.9900	-7.7988	-3.4536
**F**	-2.9318	-2.1945	-2.2764	-0.6104
**G**	2.4073	-0.9732	-2.2667	-0.5981
**H**	2.2208	-0.9486	-7.8079	-3.4628

**I**	1.4822	-0.7023	-2.2670	-0.5984
**J**	0.9187	-0.5804	6.6752	8.8137
**K**			6.1371	8.5204
**L**			6.1335	8.5196
**M**			6.6720	8.8116
**N**			6.6747	8.8140
**O**			6.1351	8.5206
**P**			6.1353	8.5212
**Q**			6.6763	8.8163
**R**			4.1587	
**S**			4.1590	

Figure S73. Calculated NICS(0) values of **3H**, **3H**, **10Pd** and **10Pd** (B3LYP/6-31G(d)).

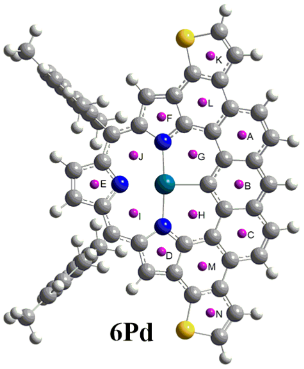

6Pd

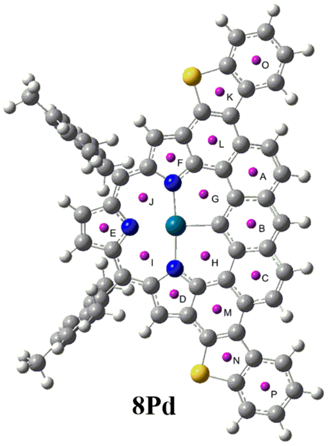

8Pd

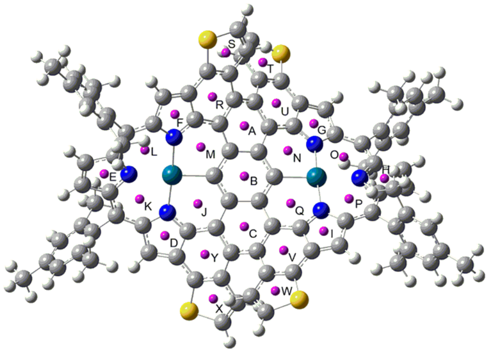

13Pd

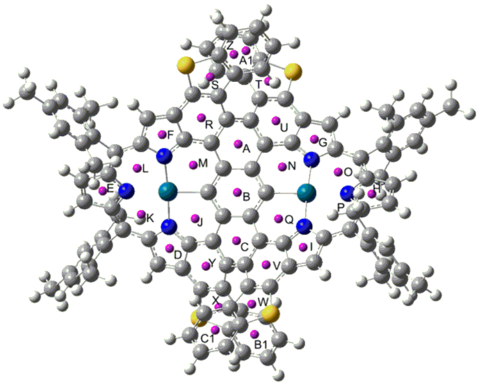

15Pd

	6Pd	8Pd	13Pd	15Pd
**A**	-2.0375	-1.6292	8.7300	9.2081
**B**	-1.8552	-0.7638	7.1298	8.2043
**C**	-2.0378	-1.4915	8.7285	9.3395
**D**	6.9204	7.8654	6.3568	6.6582
**E**	-3.0309	-2.4253	-2.1992	-1.8385
**F**	6.9196	7.6026	6.3657	6.8480
**G**	3.7881	3.4461	6.3643	6.8005
**H**	3.7883	3.8208	-2.2116	-1.7720
**I**	7.5667	7.3972	6.3681	6.6018

**J**	7.5661	7.5681	13.7592	14.5664
**K**	-8.1389	-7.1603	16.5832	17.3731
**L**	7.9234	9.1810	16.5856	17.0133
**M**	7.9244	9.1527	13.7629	14.5183
**N**	-8.1387	-7.1235	13.7645	14.3857
**O**		-9.3710	16.5866	16.9730
**P**		-9.2884	16.5848	17.0904
**Q**			13.7627	14.1146
**R**			3.5512	4.9182
**S**			-9.4408	-8.3759
**T**			-9.4422	-8.6361
**U**			3.5466	4.7366
**V**			3.5511	4.7760
**W**			-9.4434	-8.3000
**X**			-9.4473	-8.8908
**Y**			3.5470	4.8878
**Z**				-10.2921
**A1**				-10.2611
**B1**				-10.1723
**C1**				-10.3138

Figure S74. Calculated NICS(0) values of **6Pd**, **8Pd**, **13Pd** and **15Pd** (B3LYP/6-31G(d)).

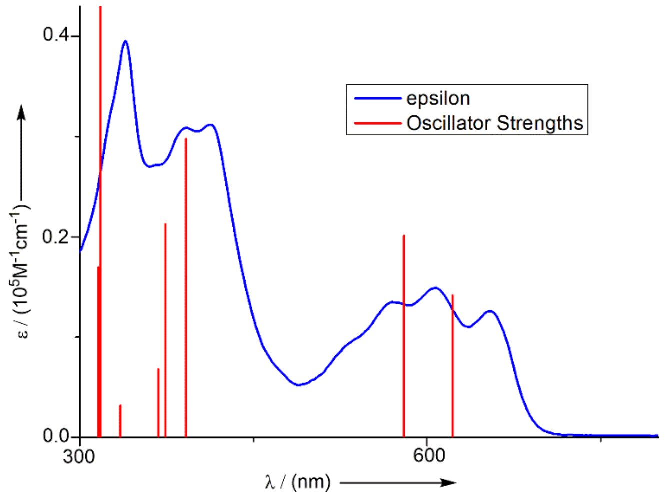

Wavelength (nm)	Oscillator Strengths (f)	Major Transitions
621.76	0.14180	HOMO → LUMO 95.2%
580.02	0.20090	HOMO-1 → LUMO 95.4%
392.09	0.29740	HOMO → LUMO+1 57.7%, HOMO-1 → LUMO+2 22.6%, HOMO-9 → LUMO 10.7%
373.5	0.21260	HOMO-1 → LUMO+1 33.2%, HOMO-8 → LUMO 24.3%, HOMO → LUMO+2 19.6%, HOMO-10 → LUMO 16.5%
367.8	0.06790	HOMO-10 → LUMO 73.3%, HOMO-12 → LUMO 7.2%, HOMO-1 → LUMO+1 5.7%, HOMO → LUMO+2 5.1%
335.3	0.03180	HOMO-13 → LUMO 41.7%, HOMO-11 → LUMO 35.7%, HOMO-9 → LUMO 19.1%
318.3	0.93650	HOMO-9 → LUMO 40.7%, HOMO-11 → LUMO 27.5%, HOMO → LUMO+1 19.6%
316.49	0.16960	HOMO-12 → LUMO 84.5%

Figure S75. Calculated vertical transitions and major transitions of **3H** calculated by TD-DFT using B3LYP

employing the 6-311G(d) basis set.

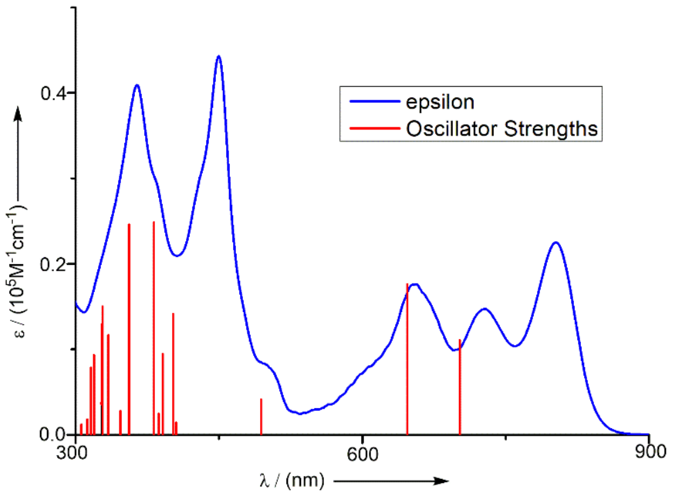

Wavelength (nm)	Oscillator Strengths (f)	Major Transitions
701.86	0.11070	HOMO → LUMO 95.2%
646.59	0.17600	HOMO-1 → LUMO 93.1%
493.67	0.04120	HOMO-4 → LUMO 93.8%
404.7	0.01440	HOMO-9 → LUMO 79.6%, HOMO → LUMO+2 8.6%
402.85	0.14140	HOMO-1 → LUMO+2 38.3%, HOMO → LUMO+1 31.2%, HOMO-10 → LUMO 25.2%
391.35	0.09460	HOMO → LUMO+1 42.1%, HOMO-1 → LUMO+2 31.5%, HOMO-11 → LUMO 20.6%
386.81	0.02460	HOMO-10 → LUMO 67.9%, HOMO-1 → LUMO+2 23.3%
382.37	0.24860	HOMO-1 → LUMO+1 41.0%, HOMO → LUMO+2 37.3%, HOMO-9 → LUMO 11.0%
355.65	0.24600	HOMO-11 → LUMO 40.3%, HOMO-14 → LUMO 25.0%, HOMO-2 → LUMO+1 13.3%, HOMO → LUMO+1 9.3%
346.58	0.02800	HOMO-2 → LUMO+1 56.2%, HOMO-14 → LUMO 13.1%, HOMO-3 → LUMO+1 9.5%
333.60	0.11650	HOMO-14 → LUMO 31.4%, HOMO-15 → LUMO 28.4%, HOMO-11 → LUMO 19.0%, HOMO-4 → LUMO+2 9.9%
328.41	0.15020	HOMO-12 → LUMO 41.1%, HOMO-1 → LUMO+3 30.2%, HOMO-1 → LUMO+4 8.1%
326.50	0.03560	HOMO-12 → LUMO 31.9%, HOMO-1 → LUMO+3 22.9%, HOMO-1 → LUMO+4 13.9%, HOMO-4 → LUMO+1 7.6%
319.10	0.09360	HOMO-3 → LUMO+3 24.1%, HOMO-3 → LUMO+4 19.0%, HOMO-2 → LUMO+3 10.6%, HOMO-14 → LUMO 8.5%, HOMO-2 → LUMO+4 7.1%, HOMO-15 → LUMO 6.8%
315.88	0.07850	HOMO-4 → LUMO+2 41.0%, HOMO-15 → LUMO 14.6%, HOMO-14 → LUMO 9.6%, HOMO-2 → LUMO+3 9.1%, HOMO-2 → LUMO+4 6.3%, HOMO-11 → LUMO 5.6%
312.02	0.01790	HOMO-2 → LUMO+3 25.5%, HOMO-2 → LUMO+4 19.6%, HOMO-3 → LUMO+3 15.0%, HOMO-3 → LUMO+4 11.5%
306.16	0.01220	HOMO-9 -> LUMO+2 36.8%, HOMO-1 -> LUMO+4 32.2%, HOMO-1 -> LUMO+3 12.6%, HOMO-16 -> LUMO 5.8%

Figure S76. Calculated vertical transitions and major transitions of **3Pd** calculated by TD-DFT using B3LYP employing the 6-311G(d) basis set.

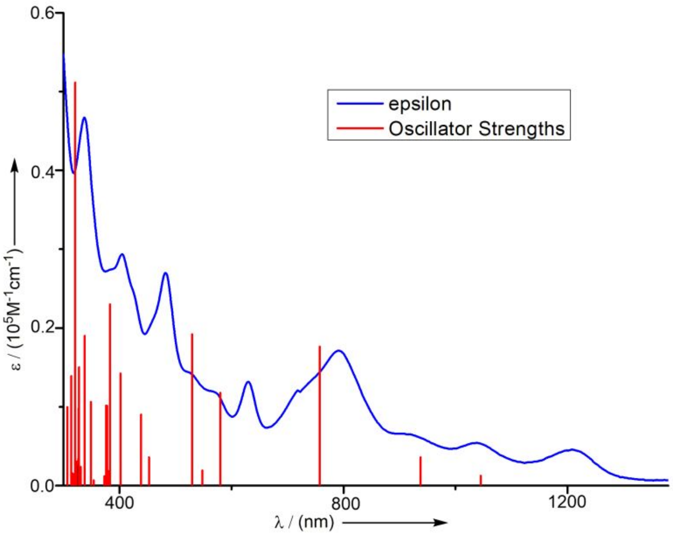

Wavelength (nm)	Oscillator Strengths (f)	Major Transitions
1045.3	0.01260	HOMO → LUMO 90.6%, HOMO-2 → LUMO 8.8%
938.3	0.03590	HOMO-1 →LUMO 94.3%
758.2	0.17640	HOMO-2 →LUMO 84.4%, HOMO →LUMO 8.1%
580.2	0.11790	HOMO-3 →LUMO 87.9%, HOMO-5 →LUMO 5.8%
548.4	0.01920	HOMO-5 → LUMO 91.9%
529.7	0.19210	HOMO-6 → LUMO 90.3%
453.4	0.03610	HOMO-1 → LUMO+1 92.0%
438.5	0.09030	HOMO → LUMO+1 62.0%, HOMO-2 → LUMO+1 22.2%, HOMO-12 → LUMO 8.2%
401.5	0.14250	HOMO-12 → LUMO 56.8%, HOMO → LUMO+1 16.4%, HOMO-2 → LUMO+1 12.9%, HOMO-13 → LUMO 8.1%
382.7	0.23000	HOMO-13 → LUMO 62.3%, HOMO → LUMO+1 9.2%, HOMO-4 → LUMO+1 7.7%, HOMO-17 → LUMO 7.4%, HOMO-2 → LUMO+1 5.2%
379.7	0.01860	HOMO-4 → LUMO+1 83.2%, HOMO-16 → LUMO 6.6%
377.9	0.10130	HOMO-15 → LUMO 36.6%, HOMO → LUMO+2 22.3%, HOMO-1 → LUMO+3 13.1%, HOMO-14 → LUMO 12.0%, HOMO-3 → LUMO+1 8.9%
375.8	0.10150	HOMO → LUMO+2 58.1%, HOMO-15 → LUMO 23.7%, HOMO-14 → LUMO 9.8%
373.3	0.01190	HOMO-1 → LIUMO+2 83.8%
353.7	0.00660	HOMO-3 → LUMO+1 31.4%, HOMO-5 →LUMO+1 26.4%, HOMO-2 → LUMO+2 20.8%
348.9	0.10630	HOMO-17 → LUMO 77.8%
338.5	0.01420	HOMO-2 → LUMO+5 32.3%, HOMO → LUMO+5 15.7%, HOMO-2 → LUMO+3 13.2%, HOMO → LUMO+3 11.7%
338.1	0.19020	HOMO-1 → LUMO+5 48.5%, HOMO-1 → LUMO+3 19.4%, HOMO → LUMO+2 5.4%
331.2	0.02360	HOMO-6 → LUMO+1 37.0%, HOMO-4 → LUMO+5 33.6%
326.1	0.02780	HOMO-4 → LUMO+2 89.4%
323.9	0.03070	HOMO-6 → LUMO+1 31.9%, HOMO → LUMO+3 22.3%, HOMO-4 → LUMO+5 17.3%, HOMO-2 → LUMO+5 8.0%
321.2	0.51140	HOMO-1 → LUMO+3 36.5%, HOMO-1 → LUMO+5 23.0%, HOMO → LUMO+2 7.8%, HOMO-6 → LUMO+2 6.8%, HOMO-4 → LUMO+2 6.8%
317.1	0.01540	HOMO-18 → LUMO 85.8%
313.6	0.13900	HOMO-2 → LUMO+3 34.4%, HOMO → LUMO+5 31.7%, HOMO-3 → LUMO+2 14.2%
306.7	0.09960	HOMO-19 → LUMO 52.9%, HOMO → LUMO+5 13.8%, HOMO-2 → LUMO+5 9.9%

Figure S77. Calculated vertical transitions and major transitions of **6Pd** calculated by TD-DFT using B3LYP employing the 6-311G(d) basis set.

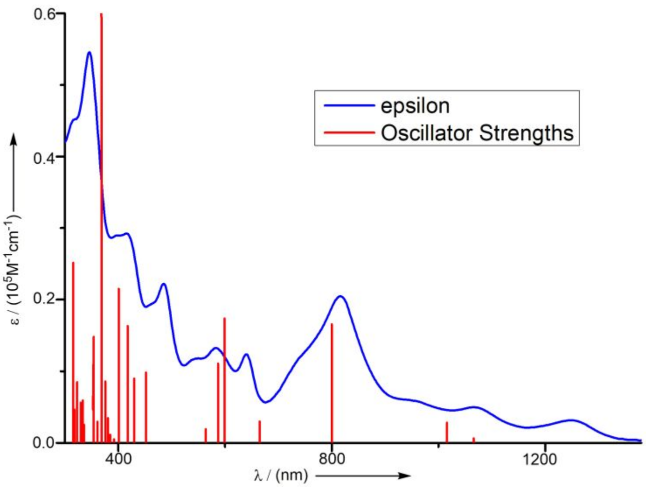

Wavelength (nm)	Oscillator Strengths (f)	Major Transitions
1065.89	0.00630	H-1 -> L 97.0%
1016.35	0.02840	H -> L 95.9%
800.47	0.1658	H-2 -> L 86.6%, H-4 -> L 8.6%
665.08	0.03010	H-5 -> L 49.8%, H-4 -> L 46.4%
599.13	0.17380	H-5 -> L 48.1%, H-4 -> L 42.9%, H-2 -> L 5.7%
587.10	0.11090	H-3 -> L 90.4%
564.21	0.01930	H-6 -> L 88.5%
452.30	0.09840	H-1 -> L+1 67.1%, H-2 -> L+1 24.3%
430.41	0.08980	H-2 -> L+1 58.0%, H-14 -> L 19.2%, H-1 -> L+1 13.9%
417.67	0.16340	H-1 -> L+2 51.6%, H -> L+3 37.9%
400.74	0.21520	H-14 -> L 57.8%, H-2 -> L+1 7.5%, H-1 -> L+1 7.4%, H-2 -> L+3 5.8%, H-16 -> L 5.5%, H -> L+2 5.0%
391.88	0.00520	H-2 -> L+2 76.9%, H-17 -> L 8.9%, H-3 -> L+1 7.1%
385.06	0.01130	H-16 -> L 56.9%, H-18 -> L 14.9%, H-19 -> L 7.1%, H-5 -> L+1 5.5%, H-4 -> L+1 5.1%
381.49	0.03470	H-4 -> L+1 41.2%, H-5 -> L+1 40.5%
375.80	0.08600	H-1 -> L+3 72.8%, H-18 -> L 7.3%, H -> L+2 5.9%
368.68	0.16760	H-2 -> L+3 71.1%
368.59	1.02220	H -> L+3 35.9%, H-1 -> L+2 34.7%, H-3 -> L+1 5.8%, H-4 -> L+2 5.8%, H-2 -> L+3 5.2%
361.09	0.02970	H-6 -> L+1 47.7%, H-3 -> L+1 17.2%, H-15 -> L 7.6%, H-17 -> L 7.2%
353.86	0.14830	H-3 -> L+1 41.0%, H-6 -> L+1 37.6%
353.79	0.04660	H-18 -> L 28.4%, H-16 -> L 14.8%, H-5 -> L+1 14.4%, H-4 -> L+1 13.4%, H-19 -> L 9.7%
335.75	0.02510	H-4 -> L+2 54.5%, H-5 -> L+2 34.1%
333.82	0.05930	H-19 -> L 61.2%, H-18 -> L 10.2%, H-3 -> L+2 8.1%, H-4 -> L+3 5.0%
330.01	0.05630	H-4 -> L+3 30.9%, H-5 -> L+3 20.6%, H-5 -> L+8 8.3%, H -> L+4 7.8%, H-19 -> L 7.2%
322.86	0.08480	H-3 -> L+2 41.6%, H-20 -> L 19.7%, H-5 -> L+3 14.1%
317.86	0.04660	H-3 -> L+3 35.5%, H -> L+8 28.5%, H -> L+6 11.8%, H -> L+5 11.0%
316.29	0.25170	H -> L+4 54.2%, H-1 -> L+5 13.9%, H-4 -> L+3 10.6%
314.92	0.07950	H-21 -> L 77.7%
304.61	0.38120	H-3 -> L+3 18.0%, H-4 -> L+2 13.3%, H -> L+5 12.1%, H-22 -> L 8.8%, H-5 -> L+2 7.4%, H-2 -> L+4 6.6%, H-21 -> L 5.0%
300.83	0.01350	H-5 -> L+3 13.2%, H-1 -> L+8 12.9%, H-2 -> L+5 11.7%, H-9 -> L+1 9.8%, H-10 -> L+1 9.7%, H-13 -> L+1 9.4%, H-4 -> L+3 6.6%, H-1 -> L+6 6.3%

Figure S78. Calculated vertical transitions and major transitions of **8Pd** calculated by TD-DFT using

B3LYP employing the 6-311G(d) basis set.

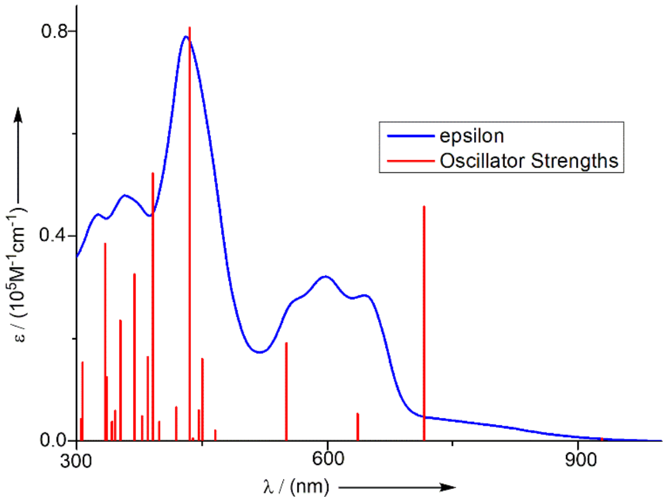

Wavelength (nm)	Oscillator Strengths (f)	Major Transitions
929.28	0.00530	H -> L 99.3%
716.3	0.45710	H-1 -> L 97.3%
635.65	0.05350	H-2 -> L 92.5%, H -> L+1 5.7%
550.55	0.19080	H -> L+1 86.9%, H-6 -> L 5.8%
465.6	0.02060	H-2 -> L+1 73.5%, H-1 -> L+2 24.8%
450.15	0.15970	H-7 -> L 48.5%, H -> L+2 28.7%, H-8 -> L 15.0%
446.1	0.06030	H-6 -> L 84.5%, H-15 -> L 6.0%
438.7	0.00520	H-4 -> L 46.7%, H-5 -> L 42.5%, H-5 -> L+1 5.1%
434.82	0.80640	H -> L+2 59.7%, H-7 -> L 20.2%, H-16 -> L 8.2%, H-8 -> L 6.2%
419.13	0.06640	H-15 -> L 86.3%
399.40	0.03700	H-18 -> L 50.5%, H -> L+3 24.0%, H-1 -> L+2 14.2%
392.02	0.08500	H-18 -> L 39.2%, H -> L+3 24.7%, H-1 -> L+2 13.4%, H-22 -> L 8.9%, H-2 -> L+1 6.2%
391.40	0.52240	H-16 -> L 48.7%, H-1 -> L+3 38.5%
384.86	0.16430	H-1 -> L+3 54.9%, H-16 -> L 23.5%, H-24 -> L 6.9%
378.08	0.04870	H-1 -> L+4 83.3%, H-2 -> L+3 12.8%
369.00	0.32560	H-20 -> L 36.3%, H-24 -> L 31.8%, H-16 -> L 12.4%, H-2 -> L+4 6.6%
352.38	0.23480	H-2 -> L+3 85.4%, H-1 -> L+4 11.6%
346.04	0.05880	H-14 -> L+1 81.2%, H-5 -> L+1 5.7%
342.2	0.03780	H-15 -> L+1 80.9%, H-9 -> L+1 7.1%
336.47	0.12500	H-22 -> L 82.4%
334.09	0.38460	H-2 -> L+4 73.2%, H-17 -> L+1 10.3%, H-20 -> L 7.3%
307.11	0.15310	H-3 -> L+2 70.7%, H-17 -> L+1 18.1%
304.52	0.04230	H-17 -> L+1 30.5%, H-19 -> L+1 27.1%, H-3 -> L+2 19.9%, H-23 -> L+1 8.3%

Figure S79. Calculated vertical transitions and major transitions of **10H** calculated by TD-DFT using B3LYP employing the 6-311G(d) basis set.

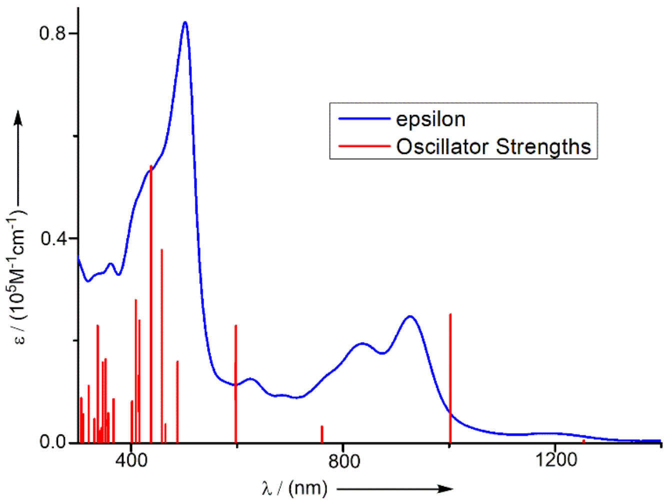

Wavelength (nm)	Oscillator Strengths (f)	Major Transitions
1254.14	0.00530	HOMO→ LUMO 99.6%
1002.54	0.25150	HOMO-1→ LUMO 95.5%
760.41	0.03220	HOMO-2→ LUMO 93.9%
598.12	0.22930	HOMO-4→ LUMO 93.2%
597.05	0.08620	HOMO→ LUMO+1 57.3%, HOMO-7→ LUMO 22.9%, HOMO-9→ LUMO 15.6%
488.15	0.15880	HOMO-9→ LUMO 77.1%, HOMO→ LUMO+1 9.4%
465.28	0.03650	HOMO-1→ LUMO+2 41.7%, HOMO-2→ LUMO+1 37.3%, HOMO→ LUMO+3 9.5%
458.23	0.37740	HOMO→ LUMO+2 59.5%, HOMO-18→ LUMO 34.2%
437.63	0.54120	HOMO-18→ LUMO 49.4%, HOMO→ LUMO+2 28.5%, HOMO-21→ LUMO 10.3%
415.87	0.23950	HOMO-1→ LUMO+3 74.6%, HOMO-21→ LUMO 10.6%, HOMO-18→ LUMO 8.2%
415.61	0.06210	HOMO-21→ LUMO 71.1%, HOMO-1→ LUMO+3 14.6%
409.05	0.27940	HOMO-21→ LUMO 71.1%, HOMO-1→ LUMO+3 14.6%
401.79	0.08120	HOMO-1→ LUMO+4 91.6%
367.28	0.08550	HOMO-23→ LUMO 59.5%, HOMO-4→ LUMO+2 34.0%
356.64	0.05830	HOMO-3→ LUMO+2 44.4%, HOMO-6→ LUMO+2 23.0%, HOMO-27→ LUMO 12.5%
355.61	0.03320	HOMO-25→ LUMO 66.8%, HOMO-27→ LUMO 15.7%, HOMO-2→ LUMO+4 6.2%
352.52	0.16400	HOMO-4→ LUMO+2 52.9%, HOMO-23→ LUMO 26.2%, HOMO-8→ LUMO+4 7.0%
347.17	0.15790	HOMO-2→ LUMO+3 47.1%, HOMO-1→ LUMO+5 21.9%, HOMO-8→ LUMO+2 11.9%
344.09	0.02910	HOMO-27→ LUMO 62.1%, HOMO-25→ LUMO 10.6%, HOMO-3→ LUMO+2 10.3%, HOMO-2→ LUMO+4 6.8%, HOMO-6→ LUMO+2 5.2%
341.69	0.02360	HOMO-8→ LUMO+2 52.5%, HOMO-4→ LUMO+4 18.0%, HOMO-1→ LUMO+5 12.6%, HOMO-5→ LUMO+3 6.7%
337.01	0.22960	HOMO-2→ LUMO+4 71.1%, HOMO-25→ LUMO 9.6%, HOMO-4→ LUMO+3 6.0%
330.91	0.04660	HOMO-5→ LUMO+3 61.6%, HOMO-20→ LUMO+1 9.8%, HOMO-28→ LUMO 8.7%, HOMO-29→ LUMO 6.2%
319.57	0.11180	HOMO-4→ LUMO+3 51.7%, HOMO-5→ LUMO+4 8.8%, HOMO-5→ LUMO+5 5.9%
308.93	0.05640	HOMO-22→ LUMO+1 17.8%, HOMO-7→ LUMO+5 14.9%, HOMO-2→ LUMO+5 13.8%, HOMO-5→ LUMO+4 10.0%, HOMO-6→ LUMO+6 8.6%, HOMO-3→ LUMO+6 6.2%
305.86	0.08800	HOMO-22→ LUMO+1 31.3%, HOMO-7→ LUMO+4 30.4%, HOMO-2→ LUMO+5 7.4%

Figure S80. Calculated vertical transitions and major transitions of **10Pd** calculated by TD-DFT using B3LYP employing the 6-311G(d) basis set.

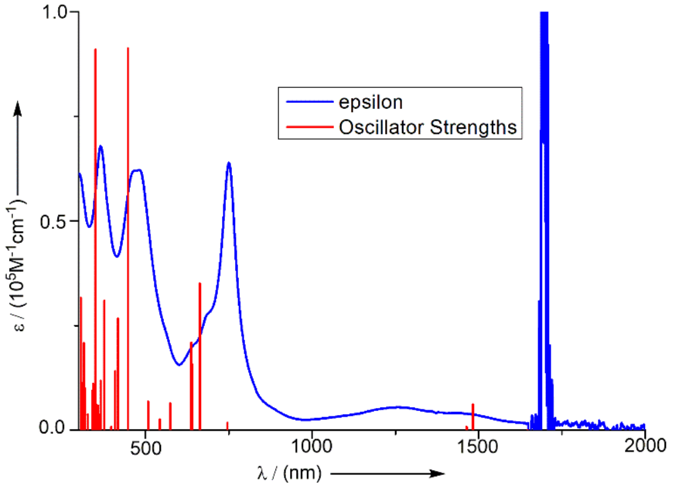

Wavelength (nm)	Oscillator Strengths (f)	Major Transitions
1647.41	0.00020	HOMO → LUMO 98.6%
1482.71	0.06170	HOMO-1 → LUMO 97.6%
1464.15	0.00820	HOMO-2 → LUMO 99.1%
745.50	0.01770	HOMO-6 → LUMO 43.9%, HOMO-2 → LUMO+1 26.9%, HOMO-10 → LUMO 22.4%
706.78	0.02110	HOMO-10 → LUMO 47.6%, HOMO-6 → LUMO 18.4%, HOMO-5 → LUMO 15.7%, HOMO-3 → LUMO+1 13.9%
662.73	0.35050	HOMO-5 → LUMO 45.0%, HOMO-2 → LUMO+1 23.2%, HOMO-10 → LUMO 20.2%, HOMO-3 → LUMO+1 6.2%
640.15	0.15750	HOMO-3 → LUMO+1 71.4%, HOMO-6 → LUMO 11.7%, HOMO-2 → LUMO+1 7.4%
636.50	0.20910	HOMO-9 → LUMO 54.5%, HOMO-8 → LUMO 40.8%
574.21	0.06380	HOMO-4 → LUMO+1 94.7%
542.96	0.02560	HOMO-5 → LUMO+1 52.3%, HOMO-6 → LUMO+1 29.2%, HOMO-1 → LUMO+2 10.1%
508.40	0.06770	HOMO-1 → LUMO+2 38.0%, HOMO-5 → LUMO+1 35.8%, HOMO-6 → LUMO+1 13.8%
448.28	0.91340	HOMO-23 → LUMO 31.2%, HOMO-2 → LUMO+2 22.9%, HOMO → LUMO+3 18.5%, HOMO-11 → LUMO+1 10.9%
417.91	0.2667	HOMO-12 → LUMO+1 41.2%, HOMO-3 → LUMO+2 40.1%, HOMO-26 → LUMO 5.4%
408.37	0.14080	HOMO-26 → LUMO 58.6%, HOMO-31 → LUMO 26.9%
377.33	0.30940	HOMO-31 → LUMO 55.5%, HOMO-26 → LUMO 20.1%, HOMO → LUMO+4 11.5%
365.52	0.11800	HOMO-1 → LUMO+5 34.9%, HOMO-5 → LUMO+3 34.7%, HOMO → LUMO+4 14.3%, HOMO-6 → LUMO+3 5.6%
362.55	0.03790	HOMO-8 → LUMO+3 62.1%, HOMO-11 → LUMO+2 18.0%
358.46	0.05830	HOMO-24 → LUMO+1 40.5%, HOMO-34 → LUMO 28.7%, HOMO-25 → LUMO+1 17.3%
356.90	0.03200	HOMO-5 → LUMO+3 29.8%, HOMO-32 → LUMO 20.9%, HOMO → LUMO+4 15.5%, HOMO-6 → LUMO+3 12.7%, HOMO-1 → LUMO+5 7.8%
353.21	0.03480	HOMO-6 → LUMO+3 70.8%, HOMO-1 → LUMO+5 9.6%, HOMO → LUMO+4 6.7%, HOMO-7 → LUMO+2 6.3%
353.04	0.06110	HOMO-25 → LUMO+1 56.1%, HOMO-34 → LUMO 32.2%
350.49	0.90920	HOMO → LUMO+5 39.3%, HOMO-1 → LUMO+4 17.7%, HOMO-9 → LUMO+3 10.9%, HOMO-34 → LUMO 9.7%
343.25	0.11080	HOMO-9 → LUMO+3 74.8%, HOMO-8 → LUMO+3 5.8%
341.77	0.09400	HOMO-33 → LUMO 79.7%
327.51	0.03690	HOMO-3 → LUMO+5 80.4%, HOMO-28 → LUMO+1 8.1%
326.39	0.03770	HOMO-27 → LUMO+1 43.3%, HOMO-29 → LUMO+1 15.3%, HOMO-36 → LUMO 14.2%
317.62	0.09980	HOMO-36 → LUMO 56.0%, HOMO-29 → LUMO+1 22.0%
315.36	0.20820	HOMO-1 → LUMO+15 64.4%

312.88	0.09460	HOMO-30 → LUMO+1 56.5%, HOMO-15 → LUMO+2 16.6%, HOMO-16 → LUMO+3 7.3%
307.98	0.02120	HOMO-37 → LUMO 35.9%, HOMO-2 → LUMO+6 16.9%, HOMO → LUMO+8 14.7%, HOMO-5 → LUMO+5 14.5%, HOMO-39 → LUMO 10.6%
307.95	0.02750	HOMO-4 → LUMO+4 45.1%, HOMO-12 → LUMO+3 24.4%, HOMO-38 → LUMO 9.0%, HOMO → LUMO+6 7.9%, HOMO-34 → LUMO+1 5.3%
307.09	0.04820	HOMO-38 → LUMO 54.3%, HOMO-4 → LUMO+4 17.6%, HOMO → LUMO+6 6.0%
306.56	0.31630	HOMO-1 → LUMO+8 53.9%, HOMO-5 → LUMO+4 21.7%, HOMO → LUMO+15 6.4%, HOMO-2 → LUMO+7 6.3%

Figure S81. Calculated vertical transitions and major transitions of **13Pd** calculated by TD-DFT using B3LYP employing the 6-311G(d) basis set.

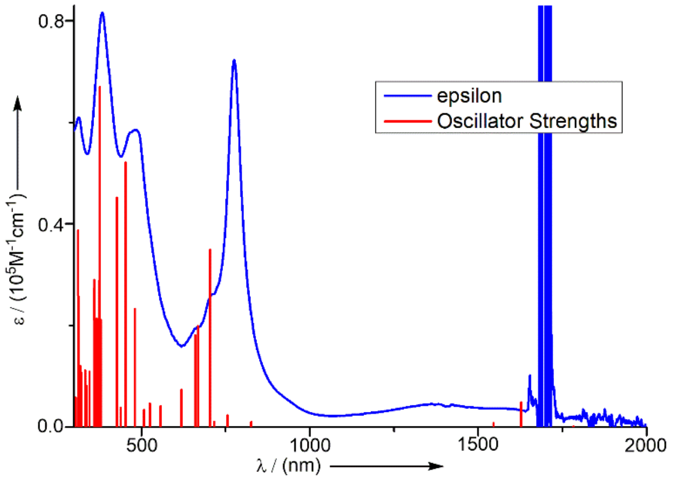

Wavelength (nm)	Oscillator Strengths (f)	Major Transitions
1782.92	0.00150	HOMO-1 → LUMO 98.3%
1627.94	0.04840	HOMO → LUMO 97.8%
1545.55	0.00790	HOMO-2 → LUMO 99.2%
826.34	0.01030	HOMO-2 → LUMO+1 53.7%, HOMO-5 → LUMO 27.3%, HOMO-8 → LUMO 9.8%, HOMO-3 → LUMO+1 6.7%
756.32	0.02300	HOMO-8 → LUMO 44.1%, HOMO-10 → LUMO 29.8%, HOMO-2 → LUMO+1 14.8%
716.26	0.01040	HOMO-10 → LUMO 38.2%, HOMO-3 → LUMO+1 25.4%, HOMO-8 → LUMO 17.9%, HOMO-5 → LUMO 13.4%
704.10	0.34940	HOMO-5 → LUMO 51.5%, HOMO-2 → LUMO+1 20.6%, HOMO-10 → LUMO 17.9%
667.98	0.19930	HOMO-3 → LUMO+1 59.9%, HOMO-8 → LUMO 19.6%, HOMO-2 → LUMO+1 6.8%
660.30	0.18110	HOMO-7 → LUMO 82.4%, HOMO-6 → LUMO 11.1%
619.02	0.07310	HOMO-4 → LUMO+1 93.8%
556.98	0.04140	HOMO-5 → LUMO+1 70.8%, HOMO → LUMO+2 11.6%, HOMO-8 → LUMO+1 10.8%
524.53	0.04640	HOMO → LUMO+2 32.8%, HOMO-10 → LUMO+1 26.2%, HOMO-5 → LUMO+1 17.6%, HOMO-8 → LUMO+1 7.4%, HOMO-18 → LUMO 7.4%
507.74	0.03400	HOMO-1 → LUMO+3 79.9%, HOMO-2 → LUMO+2 15.6%
481.27	0.23270	HOMO-2 → LUMO+2 55.0%, HOMO-26 → LUMO 24.0%, HOMO-1 → LUMO+3 7.9%, HOMO-12 → LUMO+1 6.1%
452.66	0.52100	HOMO-12 → LUMO+1 21.2%, HOMO-26 → LUMO 20.1%, HOMO-3 → LUMO+2 18.2%, HOMO-2 → LUMO+2 14.3%, HOMO-14 → LUMO+1 11.9%, HOMO-1 → LUMO+3 7.6%
437.60	0.03860	HOMO-14 → LUMO+1 70.6%, HOMO-26 → LUMO 24.5%
426.81	0.45150	HOMO-3 → LUMO+2 72.8%, HOMO-2 → LUMO+2 7.4%, HOMO-14 → LUMO+1 5.9%, HOMO-26 → LUMO 5.3%
379.84	0.21070	HOMO-1 → LUMO+5 53.3%, HOMO → LUMO+4 25.9%, HOMO-5 → LUMO+3 6.1%
375.56	0.66970	HOMO-1 → LUMO+4 28.3%, HOMO → LUMO+5 21.9%, HOMO-7 → LUMO+3 14.9%, HOMO-34 → LUMO 9.8%

374.07	0.28770	HOMO-1 → LUMO+4 28.3%, HOMO → LUMO+5 21.9%, HOMO-7 → LUMO+3 14.9%, HOMO-34 → LUMO 9.8%
367.38	0.21370	HOMO-34 → LUMO 57.8%, HOMO-28 → LUMO+1 18.7%, HOMO → LUMO+5 7.0%
364.25	0.05060	HOMO-36 → LUMO 60.9%, HOMO-35 → LUMO 11.8%, HOMO-5 → LUMO+3 11.3%
359.54	0.09480	HOMO → LUMO+6 49.4%, HOMO-7 → LUMO+3 10.8%, HOMO-7 → LUMO+2 7.1%, HOMO-6 → LUMO+3 6.1%
359.51	0.28970	HOMO-7 → LUMO+3 36.1%, HOMO-6 → LUMO+3 20.3%, HOMO → LUMO+6 14.6%, HOMO-1 → LUMO+4 6.2%
358.68	0.21120	HOMO-35 → LUMO 37.0%, HOMO-36 → LUMO 24.3%, HOMO-31 → LUMO 12.3%, HOMO-5 → LUMO+3 6.1%
346.18	0.10930	HOMO-2 → LUMO+6 82.8%
338.05	0.07250	HOMO-1 → LUMO+7 72.5%, HOMO-12 → LUMO+2 8.4%, HOMO-29 → LUMO+1 6.1%
337.26	0.08110	HOMO → LUMO+7 78.2%, HOMO-11 → LUMO+2 9.7%
333.02	0.11170	HOMO-30 → LUMO+1 41.4%, HOMO-33 → LUMO+1 18.3%, HOMO-32 → LUMO+1 6.7%, HOMO-35 → LUMO 5.5%
321.40	0.05720	HOMO-13 → LUMO+3 52.1%, HOMO → LUMO+15 20.6%
320.98	0.10740	HOMO → LUMO+15 39.5%, HOMO-13 → LUMO+3 25.9%, HOMO-14 → LUMO+2 5.6%
318.02	0.12060	HOMO-33 → LUMO+1 41.3%, HOMO-30 → LUMO+1 9.8%, HOMO-34 → LUMO+1 6.7%, HOMO-40 → LUMO 6.5%
312.98	0.06670	HOMO-42 → LUMO 56.3%, HOMO-1 → LUMO+8 15.5%, HOMO-14 → LUMO+3 8.9%
312.59	0.09820	HOMO-5 → LUMO+4 43.6%, HOMO-2 → LUMO+8 33.0%, HOMO → LUMO+10 5.6%
312.33	0.38710	HOMO-3 → LUMO+6 43.8%, HOMO-6 → LUMO+4 29.1%
305.21	0.05770	HOMO-6 → LUMO+4 43.9%, HOMO-3 → LUMO+6 23.2%, HOMO-7 → LUMO+4 12.4%

Figure S82. Calculated vertical transitions and major transitions of **15Pd** calculated by TD-DFT using B3LYP employing the 6-311G(d) basis set.

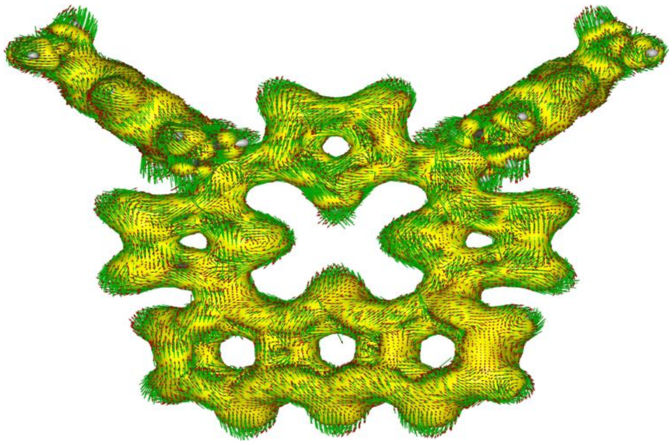

Figure S83. AICD plot of **3H** at isosurface value of 0.035. The external magnetic field was applied in the direction from the back of the paper to the surface.

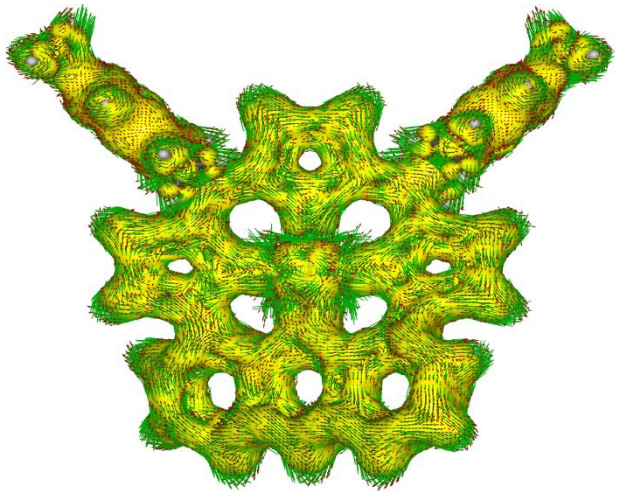

Figure S84. AICD plot of **3Pd** at isosurface value of 0.035. The external magnetic field was applied in the direction from the back of the paper to the surface.

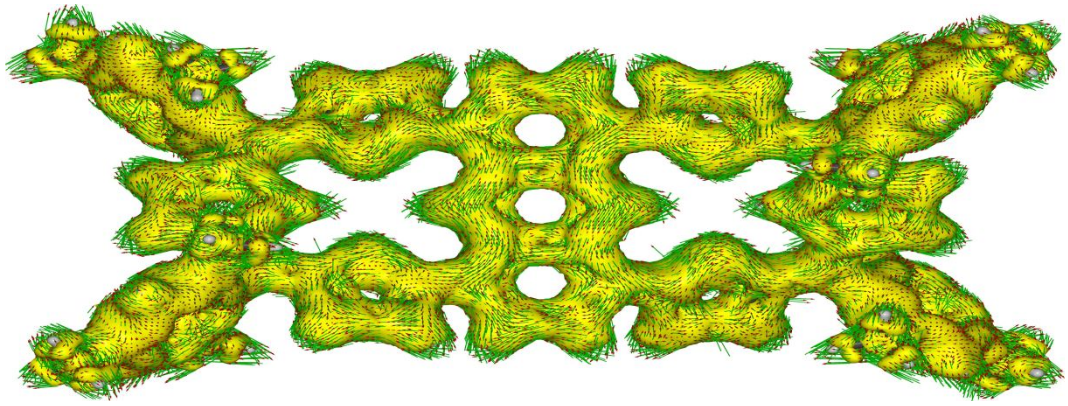

Figure S85. AICD plot of **10H** at isosurface value of 0.035. The external magnetic field was applied in the direction from the back of the paper to the surface.

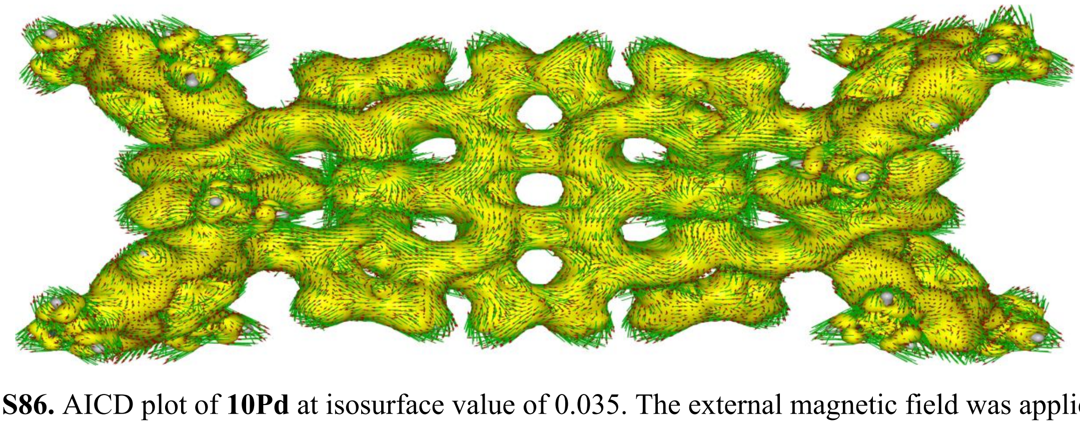

Figure S86. AICD plot of **10Pd** at isosurface value of 0.035. The external magnetic field was applied in the direction from the back of the paper to the surface.

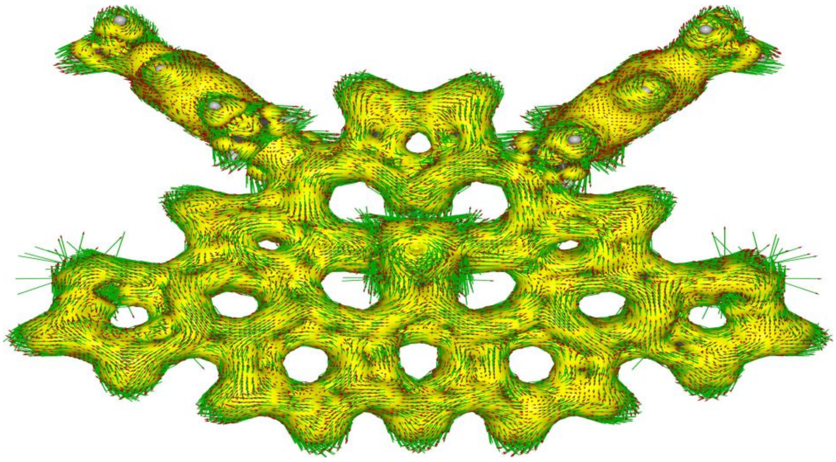

Figure S87. AICD plot of **6Pd** at isosurface value of 0.035. The external magnetic field was applied in the direction from the back of the paper to the surface.

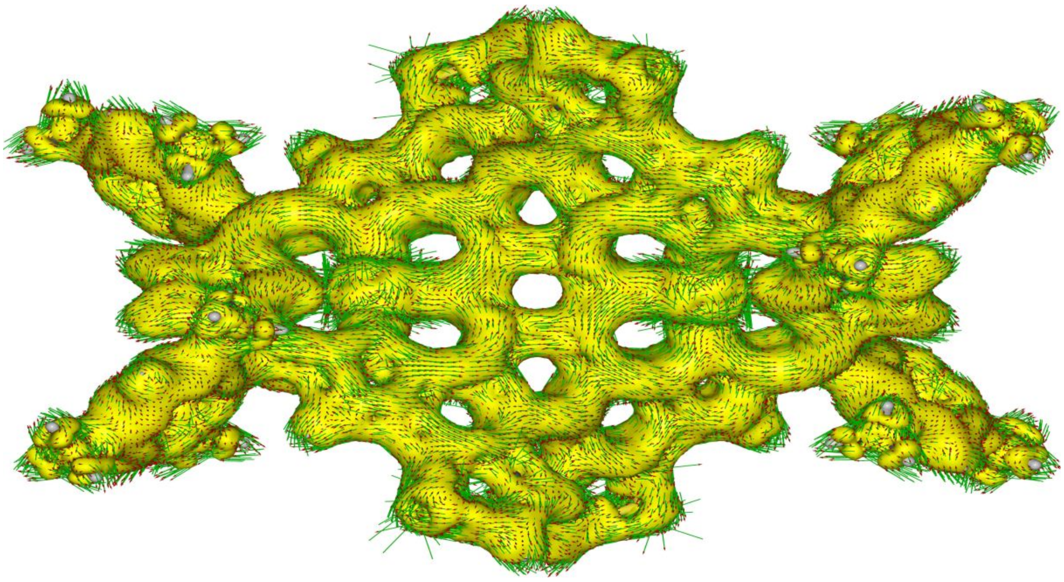

Figure S88. AICD plot of **13Pd** at isosurface value of 0.035. The external magnetic field was applied in the direction from the back of the paper to the surface.

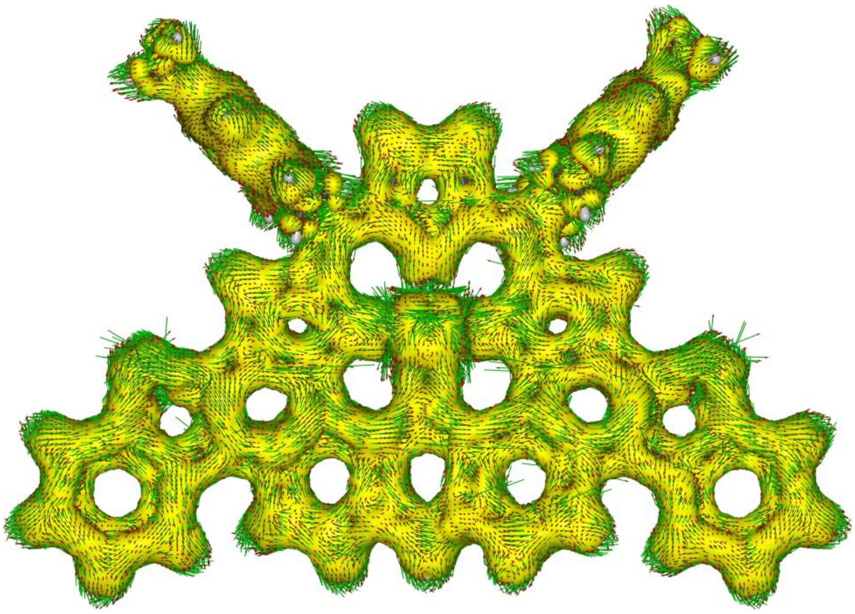

Figure S89. AICD plot of **8Pd** at isosurface value of 0.035. The external magnetic field was applied in the direction from the back of the paper to the surface.

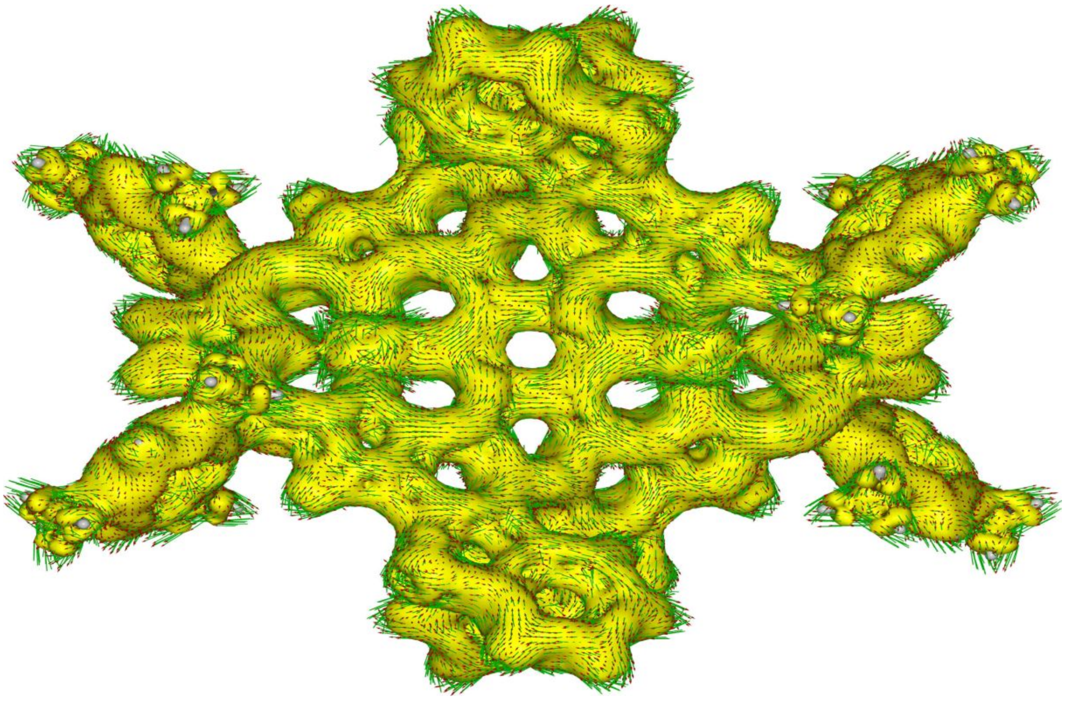

Figure S90. AICD plot of **15Pd** at isosurface value of 0.035. The external magnetic field was applied in the direction from the back of the paper to the surface.

Chiral Resolution and Racemization Experiments

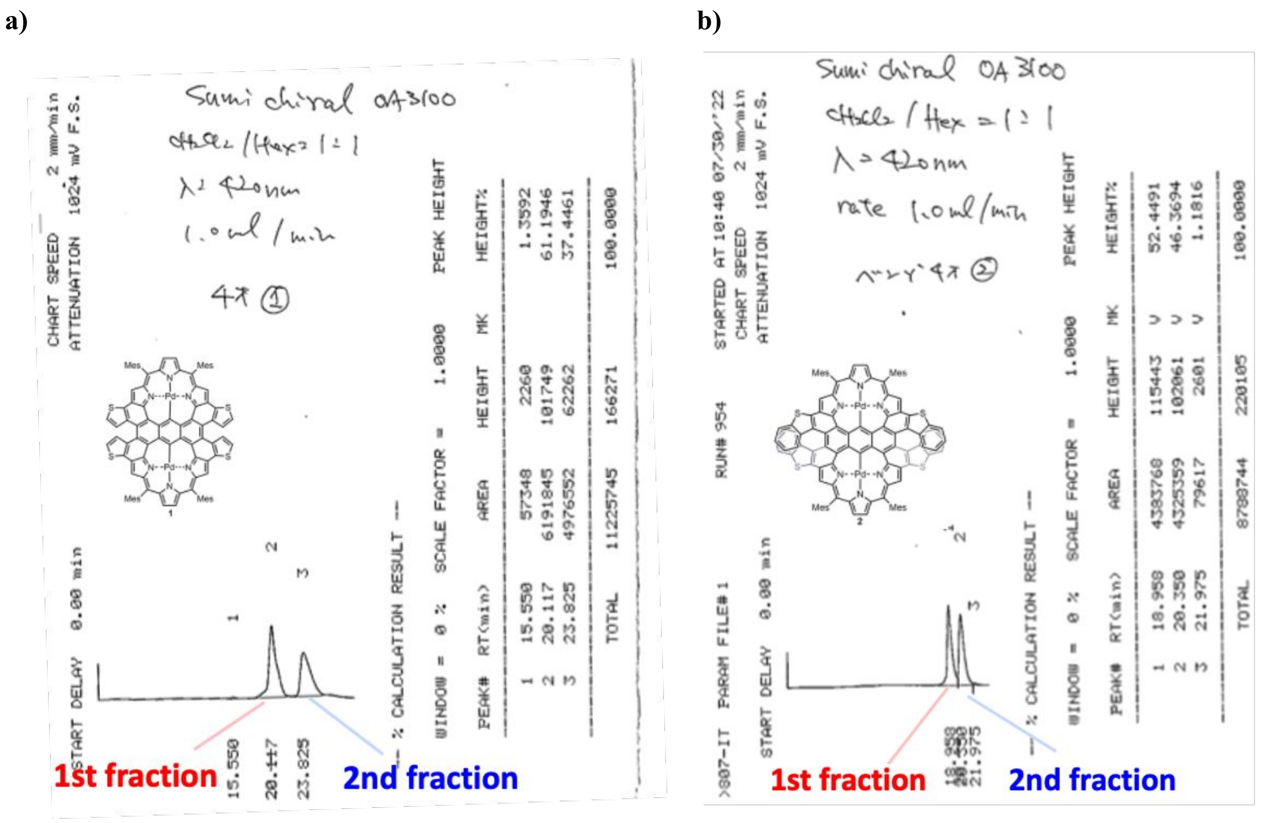

Figure S91. Preparative HPLC profile of a) **13Pd** and b) **15Pd** monitored by a UV-vis detector at 420 nm. A solution of **13Pd/15Pd** was subjected on a series of two ϕ10 x 250 mm SUMICHIRAL OA-3100 column with hexane/CH_2Cl_2 (1/1, *v*/*v*) as eluent at a flow rate of 1.0 mL min^{-1}.

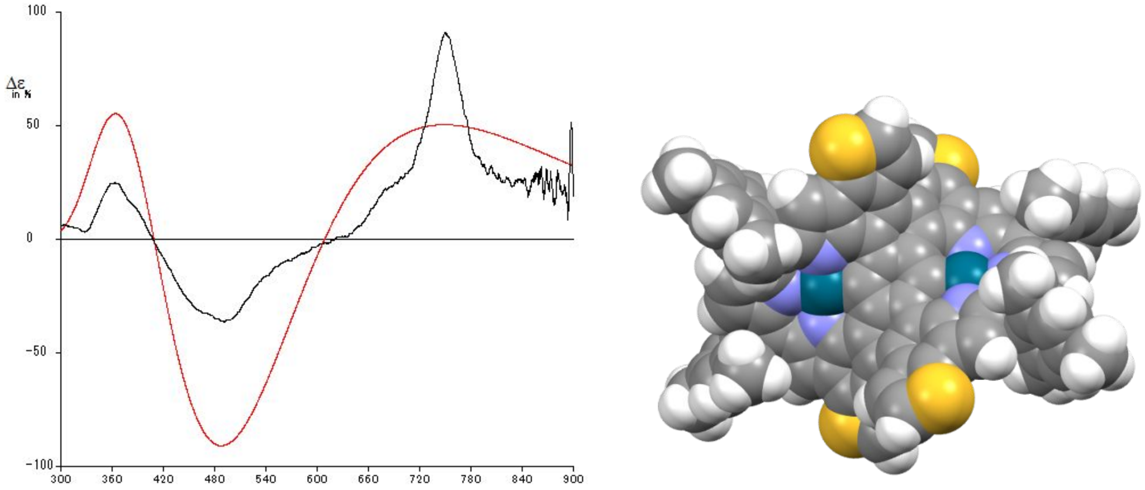

Figure S92. The CD spectrum of the second peak of **13Pd** (black) in hexane/CH_2Cl_2 (1:1) and the simulated CD spectrum of the enantiomer calculated from the structure shown above based on the TD-DFT method and using the SpecDis software package (red). From the obtained result, we assigned the absolute structure of **13Pd2** as (*M*,*M*)-dimer with a similarity factor of 0.91.

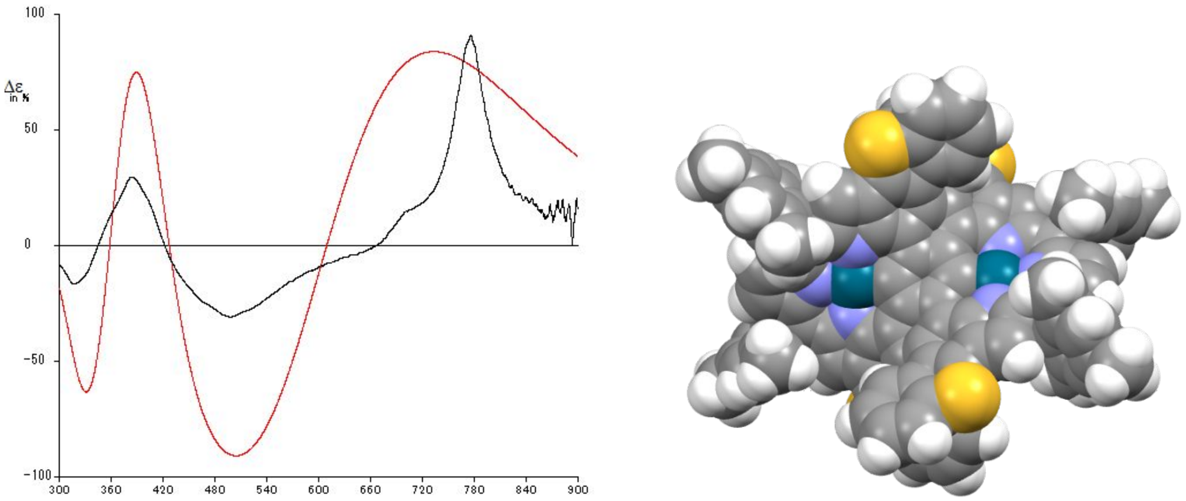

Figure S93. The CD spectrum of the second peak of **15Pd** (black) in hexane/CH_2Cl_2 (1:1) and the simulated CD spectrum of the enantiomer calculated from the structure shown above based on the TD-DFT method and using the SpecDis software package (red). From the obtained result, we assigned the absolute structure of **15Pd2** as (*M*,*M*)-dimer with a similarity factor of 0.87.

Table 10. Racemization rate constants of **13Pd**.

	80°C	90°C	100°C
k / s^{-1}	3.75 x 10^{-6}	4.86 x 10^{-6}	1.05 x 10^{-5}

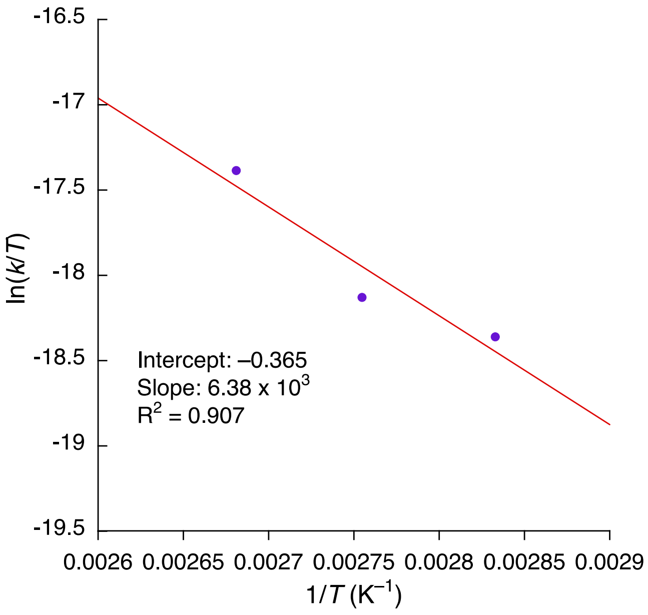

Figure S94. Eyring plot from of rate constant k and temperature of racemization process of **13Pd**.

Supporting References

[1] Gaussian 09, M. J. Frisch, G. W. Trucks, H. B. Schlegel, G. E. Scuseria, M. A. Robb, J. R. Cheeseman, G. Scalmani, V. Barone, B. Mennucci, G. A. Petersson, H. Nakatsuji, M. Caricato, X. Li, H. P Hratchian, A. F. Izmaylov, J. Bloino, G. Zheng, J. L. Sonnenberg, M. Hada, M. Ehara, K. Toyota, R. Fukuda, J. Hasegawa, M. Ishida, T. Nakajima, Y. Honda, O. Kitao, H. Nakai, T. Vreven, J. A. Montgomery, Jr., J. E. Peralta, F. Ogliaro, M. Bearpark, J. J. Heyd, E. Brothers, K. N. Kudin, V. N. Staroverov, R. Kobayashi, J. Normand, K. Raghavachari, A. Rendell, J. C. Burant, S. S. Iyengar, J. Tomasi, M. Cossi, N. Rega, J. M. Millam, M. Klene, J. E. Knox, J. B. Cross, V. Bakken, C. Adamo, J. Jaramillo, R. Gomperts, R. E. Stratmann, O. Yazyev, A. J. Austin, R. Cammi, C. Pomelli, J. W. Ochterski, R. L. Martin, K. Morokuma, V. G. Zakrzewski, G. A. Voth, P. Salvador, J. J. Dannenberg, S. Dapprich, A. D. Daniels, O. Farkas, J. B. Foresman, J. V. Ortiz, J. Cioslowski, and D. J. Fox Gaussian, Inc., Wallingford CT, 2009.
[2] A. D. Becke, *J. Chem. Phys.* **1993**, *98*, 1372-1377.
[3] C. Lee, W. Yang and R. G. Parr, *Phys. Rev. B* **1998**, *37*, 785-789.
